# Physical Activity Measurement Methodologies: A Systematic Review in the Association of South East Asian Nations (ASEAN)

**DOI:** 10.3390/sports9050069

**Published:** 2021-05-20

**Authors:** Yi-Shin Lee, John Komar, Michael Yong Hwa Chia

**Affiliations:** National Institute of Education, Nanyang Technological University, Singapore 637616, Singapore; lysleeyishin@gmail.com (Y.-S.L.); john.komar@nie.edu.sg (J.K.)

**Keywords:** physical activity, methodology, ASEAN

## Abstract

Non-communicable diseases (NCDs) are a preventable threat to livelihood and longevity in the Association of South East Asian Nations (ASEAN) and insufficient physical activity (PA) is a primary cause of NCDs. A PRISMA-based systematic review of measurement methodologies used to assess PA was conducted. 564 studies published between 1978 and 2020 were reviewed. The majority of the PA measurement employed subjective methodologies and were observational and cross-sectional, with disproportionately fewer studies conducted in economically-challenged member nations, except for Brunei. PA research in Brunei, Cambodia, Laos and Myanmar constituted 0.4–1.1% while Malaysia, Singapore, Thailand and Indonesia contributed 12–37% of all PA research within ASEAN. A decision matrix can be used to determine the measurement methodology of choice to assess PA. Joint research across ASEAN using a common assessment or measurement template that is co-curated by ASEAN researchers that incorporates multi-level and whole-of-society criteria in terms of PA enablers is a recommendation. This could be co-led by more experienced and better-resourced countries so as to produce a unified and universal ‘report card’ for PA measurement within ASEAN.

## 1. Introduction

In recent years, physical activity (PA) has increasingly come to the forefront of population-based research as regular, sufficient PA has been demonstrated to be a key factor in the prevention and management of noncommunicable diseases (NCDs) [1,2]. Specifically, sufficient PA has been previously defined by the World Health Organisation as at least 150–300 min of moderate intensity PA per week, at least 75–150 min of vigorous intensity PA per week, or an equivalent combination in healthy, adult populations, with slightly differing values for other populations [3].

PA refers to any bodily movement produced by skeletal muscles that results in energy expenditure above the resting metabolic rate, which may be unstructured and everyday life activity, exercise that includes pre-arranged deliberate and repetitive activity, and grassroots sports and competitive sports [4]. Insufficient PA is not to be confused with sedentary behaviour, defined as any waking behaviour characterised by an energy expenditure of ≤1.5 METs while in a sitting or reclining posture [5]. Thus, within the context of this discussion, samples of populations are described as physically inactive, or not having sufficient PA, if they have an insufficient PA level to meet present PA recommendations [3].

Similarly, PA is of particular importance within the Southeast Asian region as cardiovascular disease, hypertension and obesity are some of the leading health issues that have been identified in Asia [6], with global NCD-related deaths set to reach 52 million annually by 2030 [7] and the Southeast Asian region contributing to approximately a quarter of that [8], the region is set to account for approximately 13 million deaths by 2030. As of 2016, Southeast Asia reported a higher prevalence of insufficient PA levels compared to the global average [9], highlighting a pressing need for the region to implement measures to arrest further increases in the prevalence of insufficient PA levels. As a comparison, 28% of all adults aged 18 years and above were not sufficiently physically active in 2016, and while the Association of South East Asian Nations (ASEAN) average was 24.7%, 3 of the 10 ASEAN nations were above the 28% global average. 

It is important to understand how the ASEAN collective is conducting PA measurements as this determines how accurately PA levels are captured, which can affect the way future PA interventions and policies are established, as well as how accurately changes in PA levels due to the introduction of said interventions/policies can be tracked. Indeed, considerable thought is required when deciding on the appropriate methodology to use when measuring PA. It is often the case that the strength of outcomes derived from randomised controlled trials are given more credence than observational studies [10], and objective measures are valued over subjective measures [11]. However, this is not necessarily always the case, and the use of a particular methodology over another is determined by a multitude of factors, such as feasibility of the research design and the variables of interest. Therefore, the purpose of this paper was to conduct a systematic review of the current methodologies used to measure PA across studies conducted in the nations within the ASEAN group, identifying the current methodologies by which PA levels are measured and defined in ASEAN countries, and any emerging trends in the research. The papers included did not necessarily measure PA as a primary outcome, instead all papers that measured PA at all, regardless if they were measured as a dependent variable, a predictor variable, an outcome variable or a confounding variable, were included within the systematic review as our primary concern is the consolidation of the types of PA measurement methodologies being used in research produced by countries within the ASEAN collective.

## 2. Search and Selection Process

Studies examined in this systematic review were gathered according to the Preferred Reporting Items for Systematic reviews and Meta-Analyses (PRISMA) guidelines by searching through three electronic databases in May 2020, namely, i.e., PubMed, Scopus and Web of Science, to identify articles containing the following keywords and combinations: (a) Physical Activity; AND (b) Indonesia OR Philippines OR Malaysia OR Singapore OR Laos OR Brunei OR Myanmar OR Burma OR Thailand OR Cambodia OR Vietnam OR ASEAN OR Southeast Asia; AND (c) Measurement OR Method OR Survey OR Consensus.

**Figure 1:**
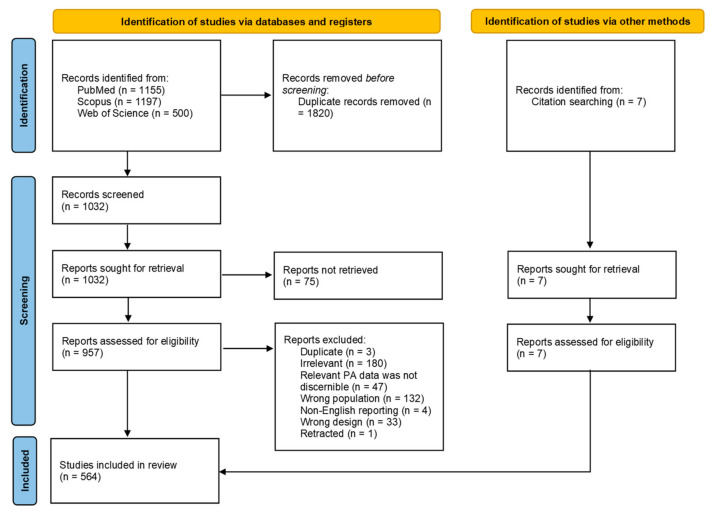
PRISMA [12] inclusion/exclusion process for the systematic review.

The 564 included articles measured PA using a variety of methodologies of research conducted on populations of the ASEAN member states, i.e., Brunei, Cambodia, Indonesia, Laos, Malaysia, Myanmar, Philippines, Singapore, Thailand, Vietnam, regardless of whether the research was conducted by institutes within the countries themselves or foreign bodies. These studies were published in the English language between 1978 and May 2020. There were initially 1155 articles found on PubMed, 1197 in Scopus and 500 in Web of Science. Rayyan QCRI was used to compile the citation lists of the three databases, whereby duplicates were flagged and manually resolved, resulting in a total of 1032 studies. 75 studies were not able to be retrieved, resulting in 957 studies. After assessing the studies for their eligibility, 400 studies were excluded and an additional seven were added after looking through references of other papers, resulting in 564 studies being included in the review. The 400 articles were excluded because they were duplicates undetected in the first round, were inaccessible, irrelevant, the PA data for the ASEAN countries were indiscernible from those of the non-ASEAN countries, the study population was wrong, was not written in English, had the wrong study design or were retracted. The PRISMA inclusion/exclusion process [12] is illustrated in Figure 1. For a full list of the 564 cited articles, please refer to Appendix A.

## 3. Overview of Publication Figures

### 3.1. Frequency of Publication, by Year

**Figure 2:**
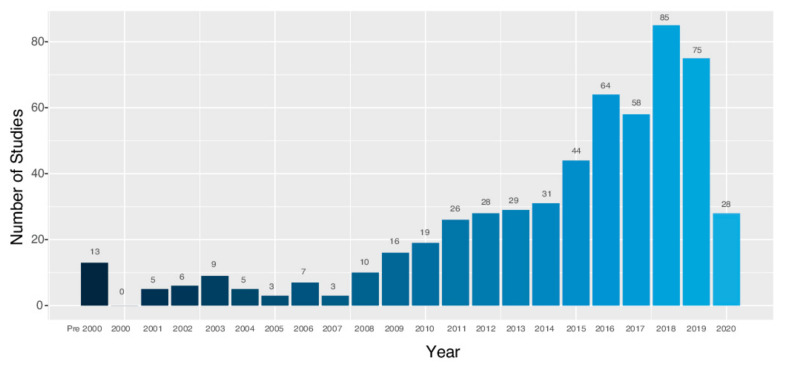
Number of studies published by year.

The distribution of the publications, by year, of the 564 articles included for review is represented graphically in Figure 2. All articles prior to 2000 were collated as a single time period as there was not at least one paper published per year from 1978 to 1999. The large decrease in the number of published papers in 2020 can be explained by the search for this literature review being completed in mid-2020. Otherwise, it is evident that there is an increasing trend in the number of papers being published on the subject matter.

### 3.2. Distribution of Studies, by Study Design

**Figure 3:**
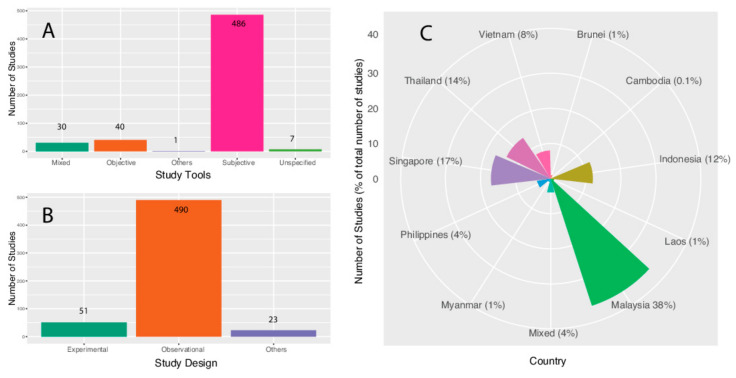
(**A**) Distribution of studies across all ASEAN nations, by study design, (**B**) by tool type and (**C**) by country.

The distribution of studies, by study design, is graphically illustrated in Figure 3A. Observational studies, including cross-sectional, case-control and longitudinal cohort studies, do not actively employ an intervention and are generally used to observe the effect of a risk factor, diagnostic test, treatment or other intervention without directly controlling for which individuals are exposed to it. Conversely, experimental studies, including quasi-experimental and randomised controlled trials (RCT), attempt to study the effects of an intervention by directly introducing an intervention [13]. The studies classified under “others” included a variety of studies such as validation studies and national surveys.

### 3.3. Distribution of Studies, by Tool Type

The distribution of studies, by tool type, is graphically illustrated in Figure 3B. Subjective measures include tools like self-report questionnaires and PA logs/diaries, while the former tends to rely more on retrospective recall across a period of time, the latter tends to utilise an approach that requires the individual to record more frequently as they perform PA. These tools are capable of monitoring a variety of PA data, i.e., intensity, frequency, duration, domain and mode. Objective measures include tools like pedometers, accelerometers, heart rate monitors and combined sensors. These tools are capable of monitoring certain types of PA data, such as intensity, frequency and duration [14].

### 3.4. Distribution of Studies, by Country

The ASEAN region features 10 member nations, i.e., Brunei, Cambodia, Indonesia, Laos, Malaysia, Myanmar, Philippines, Singapore, Thailand and Vietnam. A breakdown of the 564 studies produced by the respective ASEAN nations is graphically presented in Figure 3C. If a study contains PA data on more than one ASEAN nation, that study is categorised under “Mixed”. Table 1 presents a breakdown of the research produced by each ASEAN nation by study design, i.e., observational, experimental or “others” and tool type, i.e., subjective, objective, mixed, unspecified or “others”. 

## 4. Discussion

### 4.1. Study Design

As detailed above, the majority (87%) of studies that collected PA measures were observational in nature, while 9% were of experimental design. While experimental designs are often believed to be superior in terms of the strength of evidence produced compared to observational studies [10,15], both study designs present different sets of advantages and limitations, and thus results should be interpreted with different perspectives. Generally, interventions provide an opportunity to determine the efficacy of a protocol on affecting a given variable. RCTs are able to provide an additional layer of control through randomization by dividing participants into prognostically similar groups to better attribute an observed effect to a treatment [16]. However, experimental designs, specifically RCTs, tend to lack external validity and generalizability [17,18] due to the inclusion and exclusion criteria presented in such studies encouraging the study of a select type of population or individual. Further, experimental designs tend to preclude individuals who are less likely to adhere to or may have adverse reactions to a given intervention. This tends to result in a selection bias of individuals who may be highly motivated to engage in such an intervention, which may not necessarily be a representative sample of the population [16]. Interventions are also usually shorter in duration compared to longitudinal cohort studies. In the case of PA, approximately 50% of individuals who begin an exercise program stop within the first 6 months, a comparable statistic across many age groups [19,20]. Compare this to a recent meta-analysis [21] that demonstrated a 74% average adherence rate and 3–5% dropout rate across populations with chronic diseases in exercise intervention studies, presenting a much more optimistic outlook on exercise adherence. It is clear that the circumstances surrounding the nature of observational and experimental studies do not allow the simplistic and reductive perception of experimental studies being necessarily better than observational studies.

Observational studies, which include cross-sectional, case-control and longitudinal cohort studies, present a different set of advantages and limitations. Generally, observational studies are more easily conducted due to the lower amounts of resources required, potentially with the exception of longitudinal cohort studies, making them a cost-efficient way of identifying certain traits that may be present within certain populations before utilising experimental studies to attempt to affect the outcome. However, observational studies come with their own limitations such as left-censorship bias, right-censorship bias and confounding by indication [16]. In the case of the PA studies examined in this review, it is more likely that left-censorship bias would exist, especially in the case of longitudinal studies that examine the influence of PA on morbidity or mortality, individuals with severely debilitating conditions may not be included as they are unable to take part in such a study or have already passed on prior to their inclusion into the study due to the severity of their conditions.

As a whole, the pool of 564 studies includes both a variety of observational and experimental studies. However, Brunei, Cambodia, Laos and Myanmar lack any experimental studies. As mentioned above, both types of studies have their advantages and limitations, and thus the aforementioned countries have an incentive to develop such studies to better understand the impact of PA on individuals within their population. However, it is also worth noting that the aforementioned countries each accounted for 0.4–1.1% of the total research produced across the ASEAN nations and that the lack of research in general is an area for improvement. As mentioned earlier, observational studies are a cost-efficient way of identifying potential trends in the population before implementing experimental studies to assess the efficacy of affecting certain outcomes, and thus the lack of observational studies could perhaps also explain the lack of experimental studies. Furthermore, three of the four aforementioned nations are in the lower half of the GDP per capita ranking for ASEAN nations, and producing research in this area may not yet be a priority (Table 2). Moreover, given the relationship between increasing physical inactivity and deaths due to NCDs, it is also possible that these are not really issues that are perceived as requiring attention, especially in the case of Cambodia, Laos and Myanmar, possessing most of the lowest physical inactivity values across the ASEAN nations.

### 4.2. Tool Type

As detailed prior, the majority of studies (86.2%) utilised only subjective measures of PA, including questionnaires and activity diaries/logs, while 7.1% utilised only objective measures of PA, including accelerometers, pedometers and heart rate monitors, with 5.3% having used a mix of both types of measurement tools in their studies. As with study designs, each measurement tool type has its own advantages and limitations.

Subjective measures of PA tend to be relatively cheap and easy to administer, with the capacity to measure many different outcomes, such as frequency, intensity, duration, mode and context of PA performed [23]. Naturally, questionnaires and activity diaries/logs possess differences in what they offer to researchers, e.g., questionnaires present a lower participant burden compared to activity diaries/logs but are prone to recall bias. Subjective measures also tend to be prone to social desirability bias due to higher PA participation being viewed as more positive [24]. Nevertheless, subjective measures of PA are usually used in large-scale observational studies with many participants due to the practicality and ease of administration.

Objective measures of PA tend to be more accurate in measurement of movement but also more restricted in the information that can be gleaned. For example, while both accelerometers and pedometers can measure movement objectively, the latter is limited to the measurement of steps with no indication of intensity, duration and context of the PA. Whereas with the former, while there is a measurement of intensity and duration, there is no mode and context of the PA being carried out [23]. It is also important to note that the quality of the data gleaned from accelerometers are limited by their measurement settings, i.e., epochs and inclusion criteria for valid data. 

While most of the ASEAN nations have produced research using both subjective and objective tools, there are a few countries that have produced studies that have only utilised subjective tool types, i.e., Brunei, Cambodia and Laos. Similarly, this observation could potentially be explained by the same factors mentioned earlier regarding study design. Additionally, tool types are typically determined by the study designs [24], with surveys and observational cohort studies usually utilising subjective measures of PA and experimental designs typically utilising objective measures of PA. As such, it is of little surprise that the countries that do not have research utilising objective measures are the same countries that do not have experimental studies.

When considering the most appropriate PA measurement tool to use for assessment, it may be useful to consider a decision matrix framework [25] to arrive at a ‘best-country-culture-context’ decision. In essence, the eventual choice of the measurement tool used to measure PA is dependent upon the interaction of a variety of factors, i.e., the specific research question, feasibility and practicality, resources available and administrative considerations. It is important to be cognizant of the idea that there is no perfect instrument for every situation, especially when employed across different countries in the ASEAN region.

## 5. Future Directions

### 5.1. Consistency of Questionnaires Used across Studies

Across the 564 studies, a total of 517 questionnaires were utilised to measure PA. Of these, 252 (48.7%) were either the GPAQ, IPAQ or some population-appropriate derivative of the two. While there is little issue with the use of non-GPAQ/IPAQ questionnaires to measure PA, provided they are valid and reliable, there is a need for some form of tool use consistency if comparison across multiple countries and time periods is desired.

The use of the GPAQ/IPAQ or population-appropriate derivatives is thus especially useful for this purpose as they allow for the measurement of a diverse set of variables, including intensity, frequency, duration, context and mode. These variables can then be extensively and reliably compared across multiple countries in the region to determine PA prevalence. As such, if a common questionnaire were to be used for the aforementioned purposes of inter-nation comparison and tracking changes over time, it should be based on the parameters laid out by the GPAQ/IPAQ.

### 5.2. Population Profiles and Possible Amendments to Questionnaires

The existing WHO guidelines on PA differ across population profiles. For instance, the recommendations for adults include 150–300 min of moderate-intensity, 75–150 min of vigorous-intensity or a combined equivalent of the two intensities, of aerobic PA. It is also strongly recommended that adults perform some form of muscle strengthening activities on at least 2 days of the week. Contrast this with older adults, who are recommended to perform at least an additional 3 days per week of varied, multicomponent PA with an emphasis on functional balance and strength, on top of the aforementioned recommendations for adults [3].

As such, the GPAQ has potential room for improvement pertaining to how PA data is collected. For example, while the WHO has certain recommendations on the duration of PA that should come from muscle-strengthening exercises and varied, multicomponent PA with an emphasis on functional balance and strength in certain populations, it is not possible to capture this distinction in the current form of the GPAQ as activities are mostly categorised by intensity and domain and may not be appropriate to accurately capture the metabolic demands of, for instance, muscle-strengthening exercise. Specifically, aerobic training tends to be practiced in a continuous, cyclical form, e.g., long-distance running, making it much easier to accurately define the metabolic equivalent of work done. However, muscle-strengthening exercises are characterised by intervals of work and rest, e.g., high intensity interval training and resistance training, with the work done usually quantified by volume and not by the amount of time spent on the exercise, making the GPAQ’s current way of quantifying physical activity done based on time not as accurate in capturing the metabolic demands of said exercises. This presents an area for potential misclassification during data collection. Naturally, current iterations of the GPAQ/IPAQ would be less useful when trying to answer any questions relating to the frequency, duration and intensity of muscle-strengthening exercise an individual may be performing.

As such, it may be appropriate to develop a questionnaire that takes this into account as well.

### 5.3. Moving toward the Adoption of a Common Surveillance Framework for Physical Activity and Sedentary Behaviour

A report card on PA or inactivity, developed and promoted by Canadian researchers for children and adolescents [26], details 10 PA-enabling criteria (overall PA; organised sports and PA; active play; active transport; sedentary behaviours; family and peers; school; community and environment and government policies) that are used to assess the state of PA in countries and regions [27]. However, of note, when assessing the criterion of overall activity, only meeting age-appropriate guidelines for accumulating moderate-to-vigorous PA was considered, and not muscle or bone-strengthening exercises. Nonetheless, utilising these report cards in national surveillance studies in tandem with existing questionnaires, with the criteria jointly adapted and customised for ASEAN countries, may provide a more holistic framework for the surveillance of PA and sedentary behaviour among member nations, thus potentially strengthening research outcomes.

### 5.4. Joint Lifestyle and Time Use Research Including Physical Activity, Sedentary Behaviour, Sleep and Nutrition across ASEAN Countries

Currently, ASEAN country representatives meet at regular time horizons to discuss myriad multi-lateral issues, including topics on trade, security, and threats to health and well-being. Going forward, within the same vein of improving health and well-being across ASEAN countries, more developed countries within the bloc may perhaps consider taking the lead in working closely and co-curating the research questions with less resourced ASEAN countries for a PA health report card for ASEAN member nations. This could potentially be in the form of the provision of funding, equipment or expertise from the better resourced nations to the less resourced ones to enhance their research capabilities.

## 6. Conclusions

Results of the review of measurement methodologies used to assess PA in ASEAN show (i) the distribution of PA research is not uniform with countries such as Malaysia (37%), Singapore (17%), Thailand (14%) and Indonesia (12%) leading the pack of nations with more published research between 1978–2020. Cross-sectional and observational research using subjective methodologies to assess PA predominated. A decision-matrix to help researchers decide on the PA assessment methodologies to be used, either singly or in combination is available and should be considered. An ASEAN ‘report card’ for PA that is co-curated by member nations and administered collectively across member nations is a breakthrough recommendation. Countries outside of the ASEAN bloc may find relevance to the findings given the diverse spread of economic situations, physical inactivity levels and estimated NCD-related mortality risk of the countries within the bloc; other countries may find similarity in their circumstances and derive guidance on how to proceed from these findings.

## Figures and Tables

**Table 1:** Breakdown of type of research conducted by country.

Country	Type of Research	*n* (%)	Sub-Type of Research	*n* (%)	Tool Type	*n* (%)
Brunei	Observational	6 (100)	Cross-sectional	6 (100)	Subjective	6 (100)
			Case-control	-	Objective	-
			Longitudinal	-	Mixed	-
	Experimental	-	Quasi-experimental	-	Unspecified	-
			RCT	-	Other	-
	Other	-				
Cambodia	Observational	2 (100)	Cross-sectional	2 (100)	Subjective	2 (100)
			Case-control	-	Objective	-
			Longitudinal	-	Mixed	-
	Experimental	-	Quasi-experimental	-	Unspecified	-
			RCT	-	Other	-
	Other	-				
Indonesia	Observational	62 (88.6)	Cross-sectional	52 (74.3)	Subjective	62 (88.6)
			Case-control	4 (5.7)	Objective	3 (4.3)
			Longitudinal	6 (8.6)	Mixed	1 (1.4)
	Experimental	7 (10.0)	Quasi-experimental	3 (4.3)	Unspecified	3 (4.3)
			RCT	4 (5.7)	Other	1 (1.4)
	Other	1 (1.4)				
Laos	Observational	3 (100)	Cross-sectional	3 (100)	Subjective	3 (100)
			Case-control	-	Objective	-
			Longitudinal	-	Mixed	-
	Experimental	-	Quasi-experimental	-	Unspecified	-
			RCT	-	Other	-
	Other	-				
Malaysia	Observational	184 (86.8)	Cross-sectional	177 (83.5)	Subjective	190 (89.6)
			Case-control	6 (2.8)	Objective	10 (4.7)
			Longitudinal	1 (0.5)	Mixed	11 (5.2)
	Experimental	21 (9.9)	Quasi-experimental	9 (4.3)	Unspecified	1 (0.5)
			RCT	12 (5.7)	Other	-
	Other	7 (3.3)				
Myanmar	Observational	6 (100)	Cross-sectional	5 (83.3)	Subjective	5 (83.3)
			Case-control	-	Objective	-
			Longitudinal	1 (16.7)	Mixed	1 (16.7)
	Experimental	-	Quasi-experimental	-	Unspecified	-
			RCT	-	Other	-
	Other	-				
Philippines	Observational	21 (94.5)	Cross-sectional	17 (77.3)	Subjective	17 (77.3)
			Case-control	-	Objective	3 (13.6)
			Longitudinal	4 (18.2)	Mixed	2 (9.1)
	Experimental	1 (4.6)	Quasi-experimental	1 (4.6)	Unspecified	-
			RCT	-	Other	-
	Other	-				
Singapore	Observational	84 (85.7)	Cross-sectional	72 (73.5)	Subjective	76 (77.6)
			Case-control	1 (1.0)	Objective	16 (16.3)
			Longitudinal	11 (11.2)	Mixed	6 (6.1)
	Experimental	8 (8.2)	Quasi-experimental	3 (3.1)	Unspecified	-
			RCT	5 (5.1)	Other	-
	Other	6 (6.1)				
Thailand	Observational	67 (83.8)	Cross-sectional	61 (76.3)	Subjective	73 (91.3)
			Case-control	5 (6.3)	Objective	4 (5.0)
			Longitudinal	1 (1.3)	Mixed	2 (2.5)
	Experimental	10 (12.5)	Quasi-experimental	5 (6.3)	Unspecified	1 (1.3)
			RCT	5 (6.3)	Other	-
	Other	3 (3.8)				
Vietnam	Observational	34 (77.3)	Cross-sectional	29 (65.9)	Subjective	34 (77.3)
			Case-control	-	Objective	3 (6.8)
			Longitudinal	5 (11.4)	Mixed	5 (11.4)
	Experimental	4 (9.1)	Quasi-experimental	1 (2.3)	Unspecified	2 (4.6)
			RCT	3 (6.8)	Other	-
	Other	6 (13.6)				
Mixed	Observational	21 (100)	Cross-sectional	21 (100)	Subjective	19 (90.5)
			Case-control	-	Objective	1 (4.8)
			Longitudinal	-	Mixed	1 (4.8)
	Experimental	-	Quasi-experimental	-	Unspecified	-
			RCT	-	Other	-
	Other	-				

**Table 2:** GDP per capita, physical inactivity prevalence and estimated percentage of deaths due to noncommunicable diseases across ASEAN nations.

Country	GDP per Capita (Int$) ^1^	Physical Inactivity (%) ^2^	Estimated Percentage of Deaths Due to NCDs ^2,3^
Singapore	105,689	38	74
Brunei	85,011	26	85
Malaysia	34,567	38	74
Thailand	21,361	25	74
Indonesia	14,841	22	73
Philippines	10,094	38	67
Laos	8684	15	60
Vietnam	8677	25	77
Myanmar	7220	10	68
Cambodia	5004	10	64
ASEAN (overall)	30,115	24.7	71.6

^1^ Data taken from the International Monetary Fund [22]. ^2^ Data taken from the World Health Organisation [9]. ^3^ NCD = Noncommunicable disease.

## Data Availability

Data sharing not applicable.

## Appendix A. Full List of Reviewed Sources

1	Abdin: E., Chong, S. A., Peh, C. X., Vaingankar, J. A., Chua, B. Y., Verma, S., Jeyagurunathan, A., Shafie, S., & Subramaniam, M. (2017). The mediational role of physical activity, social contact and stroke on the association between age, education, employment and dementia in an Asian older adult population. *BMC Psychiatry*, *17*(1). https://doi.org/10.1186/s12888-017-1272-8
2	Abdul Rashid, N. A., Nurjono, M., & Lee, J. (2019). Clinical determinants of physical activity and sedentary behaviour in individuals with schizophrenia. *Asian Journal of Psychiatry*, *46*, 62–67. https://doi.org/10.1016/j.ajp.2019.10.004
3	Abdullah, M., Mohd Saat, N. Z., Muhamad Fauzi, N. F., Hui, C. Y., & Kamaralzaman, S. (2015). Association between walking and cardiovascular risk factors in university employees. *Journal of Medical Sciences (Faisalabad)*, *15*(2), 105–109. https://doi.org/10.3923/jms.2015.105.109
4	Abdullah, N. F., Teo, P. S., & Foo, L. H. (2016). Ethnic differences in the food intake patterns and its associated factors of adolescents in Kelantan, Malaysia. *Nutrients*, *8*(9). https://doi.org/10.3390/nu8090551
5	Abdullah, N., & Mohamad, N. (2016). University Recreational Facilities Service Quality and Students’ Physical Activity Level. *Procedia—Social and Behavioral Sciences*, *224*, 207–212. https://doi.org/10.1016/j.sbspro.2016.05.443
6	Abdullah, N., Kueh, Y. C., Hanafi, M. H., Morris, T., & Kuan, G. (2019). Motives for participation and amount of physical activity among Kelantan Chinese adolescents. *Malaysian Journal of Medical Sciences*, *26*(6), 101–110. https://doi.org/10.21315/mjms2019.26.6.10
7	Abidin, N. Z., Brown, W. J., Clark, B., Muhamed, A. M. C., & Singh, R. (2016). Physical activity measurement by accelerometry among older Malay adults living in semi-rural areas-a feasibility study. *Journal of Aging and Physical Activity*, *24*(4), 533–539. https://doi.org/10.1123/japa.2015-0157
8	Abu Saad, H., Kiang Soon, H., Nasir Mohd Taib, M., Abd Rahman, H., & Yoke Mun, C. (2013). Effects Of Combined Physical Activity And Dietary Intervention On Obesity And Metabolic Parameters In Adults With Abdominal Obesity. *Southeast Asian Journal of Tropical Medicine and Public Health, 44*(2).
9	Acampado, E., & Valenzuela, M. (2018). Physical activity and dietary habits of filipino college students: A cross-sectional study. *Kinesiology*, *50*(1), 57–67. https://doi.org/10.26582/k.50.1.11
10	Adair, L. S., Gultiano, S., & Suchindran, C. (2011). 20-Year trends in Filipino women’s weight reflect substantial secular and age effects. *Journal of Nutrition*, *141*(4), 667–673. https://doi.org/10.3945/jn.110.134387
11	Adair, L. S., Kuzawa, C. W., & Borja, J. (2001). Maternal energy stores and diet composition during pregnancy program adolescent blood pressure. *Circulation*, *104*(9), 1034–1039. https://doi.org/10.1161/hc3401.095037
12	Adriani, M., Diarry, V., Abdulah, R., & Wirjatmadi, B. (2015). Selenium Intake in Hypertensive and Normotensive Post-Menopausal Indonesian Women. *Journal of Nutritional Science and Vitaminology*, *61*(4), 322–325.
13	Aguila, D. V., Gironella, G. M., Capanzana, M. V. (2018). Food intake, nutritional and health status of Filipino adults according to occupations based on the 8th National Nutrition Survey 2013. *Malaysian Journal of Nutrition, 24*(3), 333-348. https://www.researchgate.net/publication/330214858
14	Ahmad, M. H., Salleh, R., Mohamad Nor, N. S., Baharuddin, A., Rodzlan Hasani, W. S., Omar, A., Jamil, A. T., Appukutty, M., Wan Muda, W. A. M., & Aris, T. (2018). Comparison between self-reported physical activity (IPAQ-SF) and pedometer among overweight and obese women in the MyBFF@home study. *BMC Women’s Health*, *18*. https://doi.org/10.1186/s12905-018-0599-8
15	Ahmad, N. S., Hamid, M. S., Cheong, J. P. G., & Hamzah, S. H. (2017). Diet and bone status in eumenorrheic female athletes. *IFMBE Proceedings*, *58*, 144–147. https://doi.org/10.1007/978-981-10-3737-5_30
16	Aji, A. S., Yerizel, E., Desmawati, & Lipoeto, N. I. (2018). The association between lifestyle and maternal vitamin D during pregnancy in West Sumatra, Indonesia. *Asia Pacific Journal of Clinical Nutrition*, *27*(6), 1286–1293. https://doi.org/10.6133/apjcn.201811_27(6).0016
17	Alagappan, M., Lekhraj, R., & Zalilah, M. S. (2019). Prevalence of overweight/obesity and its associated factors among secondary school students in semi urban area in Malaysia. *The Medical Journal of Malaysia*, *74*(6), 513–520.
18	Alias, H., Mohd Nazi, N. A., & Lau Sie Chong, D. (2019). Participation in physical activity and physical education in school among children with acute lymphoblastic leukemia after intensive chemotherapy. *Frontiers in Pediatrics*, *7*(MAR). https://doi.org/10.3389/fped.2019.00073
19	Al-Naggar, R. A., Al-Kubaisy, W., Yap, B. W., Bobryshev, Y. V., & Osman, M. T. (2015). Attitudes towards colorectal cancer (CRC) and CRC screening tests among elderly Malay patients. *Asian Pacific Journal of Cancer Prevention*, *16*(2), 667–674. https://doi.org/10.7314/APJCP.2015.16.2.667
20	Al-Sadat, N., Abdul Majid, H., Sim, P. Y., Su, T. T., Dahlui, M., Abu Bakar, M. F., Dzaki, N., Norbaya, S., Murray, L., Marie M Cantwell, M. M., & Jalaludin, M. Y. (2016). Vitamin D deficiency in Malaysian adolescents aged 13 years: findings from the Malaysian Health and Adolescents Longitudinal Research Team study (MyHeARTs). *BMJ Open*, *6*(8).
21	Amelia, R., & Harahap, J. (2019). The Role of Nutritional Status, Age, Genetic Factors, and Lifestyle on the Hypertension Prevalence among Community in Indonesian Coastal Area. *International Journal on Advanced Science, Engineering and Information Technology*, *9*(4).
22	Amini, M., Alavi-Naini, A., Azam Doustmohammadian, A. ’, Karajibani, M., Khalilian, A., Nouri-Saeedloo, S., Salimi, M., & Shafaghi, K. (2009). Relationship between childhood Obesity and physical Activity Patterns, Childhood obesity and physical activity Patterns in an urban primary school in Thailand. *Rawal Medical Journal*, *34*. https://www.researchgate.net/publication/235724460
23	Amornsriwatanakul, A., Lester, L., Bull, F. C., & Rosenberg, M. (2017). “Are Thai children and youth sufficiently active? Prevalence and correlates of physical activity from a nationally representative cross-sectional study.” *International Journal of Behavioral Nutrition and Physical Activity*, *14*(1). https://doi.org/10.1186/s12966-017-0529-4
24	Amornsriwatanakul, A., Nakornkhet, K., Katewongsa, P., Choosakul, C., Kaewmanee, T., Konharn, K., Purakom, A., Santiworakul, A., Sitilertpisan, P., Sriramatr, S., Yankai, A., Rosenberg, M., & Bull, F. C. (2016). Results from Thailand’s 2016 report card on physical activity for children and youth. *Journal of Physical Activity and Health*, *13*(11), S291–S298. https://doi.org/10.1123/jpah.2016-0316
25	Ang, B. W., Yee Tan, M., Goh, C. M., Rahardja, S., Lim, B. Y., Chiew, W., Heng, T. Y., Ian Tan, K., Foo, J. H., Tham, S. Z., Chng, J. K., Jie Seow, W., & Luo, N. (2019). Impact of Knowledge and Attitudes on Lifestyle Practices in Preventing Type 2 Diabetes Mellitus. *Annals of the Academy of Medicine, Singapore*, *48*(8), 247–263.
26	Ang, L. W., Cutter, J., James, L., & Goh, K. T. (2017). Factors associated with influenza vaccine uptake in older adults living in the community in Singapore. *Epidemiology and Infection*, *145*(4), 775–786. https://doi.org/10.1017/S0950268816002491
27	Ang, M. S., Nurjono, M., & Lee, J. (2019). The effects of clinical illness severity and physical activity on health-related quality of life in schizophrenia. *Quality of Life Research*, *28*(6), 1509–1520. https://doi.org/10.1007/s11136-019-02126-8
28	Angeles-Agdeppa, I., Lana MStat, R. D., & Barba, C. V. (2003). A case study on dual forms of malnutrition among selected households in District 1, Tondo, Manila. *Asia Pacific Journal of Clinical Nutrition, 12*(4).
29	Aniza, I., & Fairuz, M. R. (2009). Factors influencing physical activity level among secondary school adolescents in Petaling District, Selangor. *The Medical Journal of Malaysia*, *64*(3), 228–232.
30	Anuradha, S., Healy, G. N., Dunstan, D. W., Shyong Tai, E., Van Dam, R. M., Lee, J., Khaing Nang, E. E., Owen, N., & Wong, T. Y. (2011). Associations of physical activity and television viewing time with retinal vascular caliber in a multiethnic asian population. *Investigative Ophthalmology and Visual Science*, *52*(9), 6522–6528. https://doi.org/10.1167/iovs.11-7324
31	Apidechkul, T. (2018). Prevalence and factors associated with type 2 diabetes mellitus and hypertension among the hill tribe elderly populations in northern Thailand. *BMC Public Health*, *18*(1). https://doi.org/10.1186/s12889-018-5607-2
32	Apriana, W., Sofiyya, I., Julia, M., & Huriyati, E. (2016). Relationship Between Physical Activity and Concentration of Study of Teenagers in Yogyakarta, Indonesia. *Pakistan Journal of Nutrition*, *15*(3), 211–216.
33	Ariffin, F. D., Ismail, A., Tan, V. P. S., Yusoff, Z., Awang, S. A., Rani, W. R. W. A., & Rasool, A. H. G. (2014). Improved insulin sensitivity, central systolic pressure and inflammatory indicators achieved with minor weight reduction in overweight and obese subjects given education on lifestyle modification. *Asian Biomedicine*, *8*(2), 185–194. https://doi.org/10.5372/1905-7415.0802.278
34	Arifwidodo, S. D., & Chandrasiri, O. (2020). Association between park characteristics and park-based physical activity using systematic observation: Insights from Bangkok, Thailand. *Sustainability (Switzerland)*, *12*(6). https://doi.org/10.3390/su12062559
35	Arma, N., Rosnah, I., & Noor Hassim, I. (2017). Psychometric properties of the Malay-translated General Practice Physical Activity Questionnaire among shipyard workers. *The Medical Journal of Malaysia*, *72*(6), 350–355.
36	Arundhana, A. I. (2007). Analysis of the Cost Effectiveness of Improving Nutrition Intake and nutritional status in Patients of Reproductive Age Undergoing Haemodialysis therapy in Makassar. *Malaysian Journal of Nutrition*. https://www.researchgate.net/publication/329282230
37	Aryani, I., Rachma, U. P., Rokhayati, E., & Moelyo, A. G. (2018). Menstrual cycle patterns of Indonesian adolescents. *Paediatrica Indonesiana*, *58*(3), 101–105. https://doi.org/10.14238/pi58.3.2018.101-5
38	Ar-yuwat, S., Clark, Hunter, & James. (2013). Determinants of physical activity in primary school students using the health belief model. *Journal of Multidisciplinary Healthcare*, 119. https://doi.org/10.2147/jmdh.s40876
39	Awal, M., Amiruddin, R., Palutturi, S., & Mallongi, A. (2017). Relationships between lifestyle models with stroke occurrence in South Sulawesi, Indonesia. *Asian Journal of Epidemiology*, *10*(2), 83–88. https://doi.org/10.3923/aje.2017.83.88
40	Baharudin, A., Zainuddin, A. A., Manickam, M. A., Ambak, R., Ahmad, M. H., Naidu, B. M., Cheong, S. M., Ying, C. Y., Saad, H. A., & Ahmad, N. A. (2014). Factors associated with physical inactivity among school-going adolescents: data from the Malaysian School-Based Nutrition Survey 2012. *Asia-Pacific Journal of Public Health / Asia-Pacific Academic Consortium for Public Health*, *26*(5 Suppl). https://doi.org/10.1177/1010539514543682
41	Banks, E., Lim, L., Seubsman, S. A., Bain, C., & Sleigh, A. (2011). Relationship of obesity to physical activity, domestic activities, and sedentary behaviours: Cross-sectional findings from a national cohort of over 70,000 Thai adults. *BMC Public Health*, *11*. https://doi.org/10.1186/1471-2458-11-762
42	Bauman, A., Ma, G., Cuevas, F., Omar, Z., Waqanivalu, T., Phongsavan, P., Keke, K., & Bhushan, A. (2011). Differences in the prevalence of leisure-time and occupational physical activity, and active commuting in six Asia-Pacific countries. *Journal of Epidemiology and Community Health*, *65*(1), 35–43. https://doi.org/10.1136/jech.2008.086710
43	Belakang, S., Risikonya, F., Prasiswazah, K., Kesihatan, S., Nordin, M., Kaur, D., Singh, A., & Kanglun, L. (2014). Low Back Pain and Associated Risk Factors among Health Science Undergraduates. *Sains Malaysiana, 43*(3).
44	Belmonte Gonzalez-Suarez, C., & Grimmer-Somers, K. (2011). The Association of Physical Activity and Physical Fitness With Pre-Adolescent Obesity: An Observational Study in Metromanila, Philippines. *Journal of Physical Activity and Health, 8*.
45	Bernard, J. Y., Ng, S., Natarajan, P., Loy, S. L., Aris, I. M., Tint, M. T., Chong, Y. S., Shek, L., Chan, J., Godfrey, K. M., Khoo, C. M., Leow, M. K. S., Müller-Riemenschneider, F., & Chan, S. Y. (2019). Associations of physical activity levels and screen time with oral glucose tolerance test profiles in Singaporean women of reproductive age actively trying to conceive: the S-PRESTO study. *Diabetic Medicine*, *36*(7), 888–897. https://doi.org/10.1111/dme.13948
46	Beshir, S. A., & Bt Hamzah, N. H. (2014). Health promotion and health education: Perception, barriers and standard of practices of community pharmacists. *International Journal of Health Promotion and Education*, *52*(4), 174–180. https://doi.org/10.1080/14635240.2014.888809
47	Bharu, K., Kelantan, M., Putri, W., Dali, E. W., Jan, H., Mohamed, J., & Yusoff, H. (2018). Nutrient Intakes Status and Physical Inactivity among Overweight and Obese School Children in Kota Bharu, Kelantan, Malaysia. *Iranian Journal of Public Health, 47*(8). http://ijph.tums.ac.ir
48	Bich Thuy, A., Blizzard, L., Schmidt, M., Magnussen, C., Hansen, E., & Dwyer, T. (2011). Using Pedometers to Estimate Ambulatory Physical Activity in Vietnam. *Journal of Physical Activity and Health*, *8*, 52–61.
49	Binh, T. Q., Phuong, P. T., & Nhung, B. T. (2015). Knowledge and associated factors towards type 2 diabetes among a rural population in the Red River Delta region, Vietnam. *Rural and Remote Health*, *15*(3). http://www.rrh.org.au
50	Bishop, G. D., Pek, J., & Ngau, F. (2006). Blunted cardiovascular responses to daytime activities as related to reduced nocturnal blood pressure decline. *Annals of Behavioral Medicine*, *31*(3), 248–253. https://doi.org/10.1207/s15324796abm3103_6
51	Bjertness, M. B., Htet, A. S., Meyer, H. E., Htike, M. M. T., Zaw, K. K., Oo, W. M., Latt, T. S., Sherpa, L. Y., & Bjertness, E. (2016). Prevalence and determinants of hypertension in Myanmar—A nationwide cross-sectional study. *BMC Public Health*, *16*(1). https://doi.org/10.1186/s12889-016-3275-7
52	Boonyaratavej, N., Suriyawongpaisal, P., Takkinsatien, A., Wanvarie, S., Rajatanavin, R., & Apiyasawat, P. (2001). Physical activity and risk factors for hip fractures in Thai women. *Osteoporosis International*, *12*(3), 244–248. https://doi.org/10.1007/s001980170136
53	Botabara-Yap, M. J., Estrada, M. R., & Balila, E. (2019). Lifestyle Predictors Of Overweight Among Malaysians. *Malaysian Journal of Public Health Medicine, 19*(1).
54	Bui Van, N., Pham Van, Q., Vo Hoang, L., Bui Van, T., Nguyen Hoang, N., Do Nam, K., & Chu, D. T. (2018). Prevalence and Risk Factors of Hypertension in Two Communes in the Vietnam Northern Mountainous, 2017. *BioMed Research International*, *2018*. https://doi.org/10.1155/2018/7814195
55	Bui, T. Van, Blizzard, C. L., Luong, K. N., Truong, N. L. Van, Tran, B. Q., Otahal, P., Gall, S., Nelson, M. R., Au, T. B., Ha, S. T., Phung, H. N., Tran, M. H., Callisaya, M., & Srikanth, V. (2016). National survey of risk factors for non-communicable disease in Vietnam: Prevalence estimates and an assessment of their validity. *BMC Public Health, 16*(1). https://doi.org/10.1186/s12889-016-3160-4
56	Chai, S., Kueh, Y. C., Yaacob, N. M., & Kuan, G. (2019). Psychometric properties of the Malay version of the goal content for exercise questionnaire among undergraduate students at the Health Campus, Universiti Sains Malaysia. *Malaysian Journal of Medical Sciences*, *26*(1), 115–124. https://doi.org/10.21315/mjms2019.26.1.11
57	Chai, Z. F., Gan, W. Y., Chin, Y. S., Ching, Y. K., & Appukutty, M. (2019). Factors associated with anemia among female adult vegetarians in Malaysia. *Nutrition Research and Practice*, *13*(1), 23–31. https://doi.org/10.4162/nrp.2019.13.1.23
58	Chaising, S., & Temdee, P. (2020). Determining Significant Risk Factors for Preventing Elderly People with Hypertension from Cardiovascular Disease Complication Using Maximum Objective Distance. *Wireless Personal Communications*, *115*(4), 3099–3122. https://doi.org/10.1007/s11277-020-07195-4
59	Chan, C. Y., Subramaniam, S., Chin, K. Y., Ima-Nirwana, S., Muhammad, N., Fairus, A., Rizal, A. M. M., Ng, P. Y., Aini, J. N., Aziz, N. A., & Mohamed, N. (2019). Knowledge, beliefs, dietary, and lifestyle practices related to bone health among middle-aged and elderly Chinese in Klang Valley, Malaysia. *International Journal of Environmental Research and Public Health*, *16*(10). https://doi.org/10.3390/ijerph16101787
60	Chan, D. K. C., Stenling, A., Yusainy, C., Hikmiah, Z., Ivarsson, A., Hagger, M. S., Rhodes, R. E., & Beauchamp, M. R. (2020). Editor’s Choice: Consistency tendency and the theory of planned behavior: a randomized controlled crossover trial in a physical activity context. *Psychology and Health*, *35*(6), 665–684. https://doi.org/10.1080/08870446.2019.1677904
61	Chan, W. K., Tan, A. T. B., Vethakkan, S. R., Tah, P. C., Vijayananthan, A., & Goh, K. L. (2013). Non-alcoholic fatty liver disease in diabetics—prevalence and predictive factors in a multiracial hospital clinic population in Malaysia. *Journal of Gastroenterology and Hepatology (Australia)*, *28*(8), 1375–1383. https://doi.org/10.1111/jgh.12204
62	Chan, W. K., Tan, A. T. B., Vethakkan, S. R., Tah, P. C., Vijayananthan, A., & Goh, K. L. (2014). Ultrasonography-diagnosed non-alcoholic fatty liver disease is not associated with prevalent ischemic heart disease among diabetics in a multiracial Asian hospital clinic population. *Clinics and Research in Hepatology and Gastroenterology*, *38*(3), 284–291. https://doi.org/10.1016/j.clinre.2014.02.009
63	Chan, W. K., Tan, A. T. B., Vethakkan, S. R., Tah, P. C., Vijayananthan, A., & Goh, K. L. (2015). Low physical activity and energy dense Malaysian foods are associated with non-alcoholic fatty liver disease in centrally obese but not in non-centrally obese patients with diabetes mellitus. *Asia Pacific Journal of Clinical Nutrition*, *24*(2), 289–298. https://doi.org/10.6133/apjcn.2015.24.2.15
64	Chan, Y. Y., Lim, K. K., Lim, K. H., Teh, C. H., Kee, C. C., Cheong, S. M., Khoo, Y. Y., Baharudin, A., Ling, M. Y., Omar, M. A., & Ahmad, N. A. (2017). Physical activity and overweight/obesity among Malaysian adults: Findings from the 2015 National Health and morbidity survey (NHMS). *BMC Public Health, 17*(1). https://doi.org/10.1186/s12889-017-4772-z
65	Chan, Y. Y., Sooryanarayana, R., Mohamad Kasim, N., Lim, K. K., Cheong, S. M., Kee, C. C., Lim, K. H., Omar, M. A., Ahmad, N. A., & Mohd Hairi, N. N. (2019). Prevalence and correlates of physical inactivity among older adults in Malaysia: Findings from the National Health and Morbidity Survey (NHMS) 2015. *Archives of Gerontology and Geriatrics*, *81*, 74–83. https://doi.org/10.1016/j.archger.2018.11.012
66	Chandrasiri, O., & Arifwidodo, S. (2017). Inequality in Active Public Park: A Case Study of Benjakitti Park in Bangkok, Thailand. *Procedia Engineering*, *198*, 193–199. https://doi.org/10.1016/j.proeng.2017.07.083
67	Chang, C. T., Cheah, W. L., Hazmi, H., & Wan Muda, W. A. M. (2016). Domain-specific physical activity among indigenous overweight and obese communities in Sarawak. *Baltic Journal of Health and Physical Activity*, *8*(3), 40–48. https://doi.org/10.29359/bjhpa.08.3.05
68	Chavasit, V., Kriengsinyos, W., Photi, J., & Tontisirin, K. (2017). Trends of increases in potential risk factors and prevalence rates of diabetes mellitus in Thailand. *European Journal of Clinical Nutrition, 71*(7), 839-843. https://doi.org/10.1038/ejcn.2017.52
69	Chawla, N., Panza, A., Sirikulchayanonta, C., & Kumar, R. (2017). Effectiveness Of A School-Based Multicomponent Intervention On Nutritional Status Among Primary School Children In Bangkok, Thailand. *Journal of Ayub Medical College Abbottabad, 29*(1). http://www.jamc.ayubmed.edu.pk13
70	Cheah, W. L., Chang, C. T., & Saimon, R. (2012). Environment factors associated with adolescents’ body mass index, physical activity and physical fitness in Kuching south city, Sarawak: A cross-sectional study. *International Journal of Adolescent Medicine and Health*, *24*(4), 331–337. https://doi.org/10.1515/ijamh.2012.048
71	Cheah, W. L., Chang, C. T., Hazmi, H., & Wan Muda, W. M. (2016). Gender and Racial Differences in the Cardiovascular Risk Factors among Overweight and Obese Rural Adults, Kuching and Samarahan Division, Sarawak, Malaysia. *Journal of Nutrition and Metabolism*, *2016*. https://doi.org/10.1155/2016/4536753
72	Cheah, W. L., Helmy, H., & Chang, C. T. (2014). Factors associated with physical inactivity among female and male rural adolescents in Borneo—A cross-sectional study. *International Journal of Adolescent Medicine and Health*, *26*(3), 447–453. https://doi.org/10.1515/ijamh-2013-0319
73	Cheah, W., Hazmi, H., & Chang, C. (2015). Predictors of physical activity for weekdays and weekends among adolescent—a cross-sectional study in Sarawak, Malaysia. *Baltic Journal of Health and Physical Activity*, *7*(1), 42–50.
74	Cheah, Y. (2011). Influence of Socio-demographic Factors on Physical Activity Participation of Adults in Penang Influence of Socio-demographic Factors on Physical Activity Participation in a Sample of Adults in Penang, Malaysia. *Malaysian Journal of Nutrition*, *17*(3), 385–391.
75	Cheah, Y. K. (2012). An Exploratory Study on Self-rated Health Status: The Case of Penang. *Malaysia Malaysian Journal of Economic Studies*, *49*(2), 141–155.
76	Cheah, Y. K., & Poh, B. K. (2014). The Determinants of Participation in Physical Activity in Malaysia. *Osong Public Health and Research Perspectives*, *5*(1), 20–27. https://doi.org/10.1016/j.phrp.2013.12.002
77	Cheah, Y. K., Azahadi, M., Phang, S. N., & Abd Manaf, N. H. (2019). Vigorous and moderate physical activity among overweight and obese adults in Malaysia: Sociodemographic correlates. *Obesity Medicine*, *15*. https://doi.org/10.1016/j.obmed.2019.100114
78	Cheah, Y. K., Azahadi, M., Phang, S. N., & Manaf, N. H. A. (2019). Association of Suicidal Ideation with Demographic, Lifestyle and Health Factors in Malaysians. *East Asian Archives of Psychiatry*. https://doi.org/10.12809/eaap1731
79	Cheah, Y. K., Lim, H. K., & Kee, C. C. (2018). Demographic and lifestyle determinants of time spent in physical activity among Malaysian adolescents. *International Journal of Pediatrics and Adolescent Medicine*, *5*(2), 49–54. https://doi.org/10.1016/j.ijpam.2018.02.001
80	Cheah, Y. K., Lim, H. K., & Kee, C. C. (2018). Factors associated with underage sex in Malaysia: A secondary data analysis of Malaysia Global School-Based Student Health Survey 2012. *Journal of Population and Social Studies*, *26*(4), 305–320. https://doi.org/10.25133/JPSSv26n4.021
81	Cheah, Y. K., Lim, H. K., Kee, C. C., & Ghazali, S. M. (2015). Factors associated with participation in physical activity among adolescents in Malaysia. *International Journal of Adolescent Medicine and Health*, *2015*. https://doi.org/10.1515/ijamh-2015-0030
82	Chen, B., Bernard, J. Y., Padmapriya, N., Ning, Y., Cai, S., Lança, C., Tan, K. H., Yap, F., Chong, Y. S., Shek, L., Godfrey, K. M., Saw, S. M., Chan, S. Y., Eriksson, J. G., Tan, C. S., & Müller-Riemenschneider, F. (2020). Associations between early-life screen viewing and 24 h movement behaviours: findings from a longitudinal birth cohort study. *The Lancet Child and Adolescent Health*, *4*(3), 201–209. https://doi.org/10.1016/S2352-4642(19)30424-9
83	Chen, B., Bernard, J. Y., Padmapriya, N., Yao, J., Goh, C., Tan, K. H., Yap, F., Chong, Y. S., Shek, L., Godfrey, K. M., Chan, S. Y., Eriksson, J. G., & Müller-Riemenschneider, F. (2019). Socio-demographic and maternal predictors of adherence to 24-h movement guidelines in Singaporean children. *International Journal of Behavioral Nutrition and Physical Activity*, *16*(1). https://doi.org/10.1186/s12966-019-0834-1
84	Chen, B., Waters, C. N., Compier, T., Uijtdewilligen, L., Petrunoff, N. A., Lim, Y. W., Van Dam, R., & Müller-Riemenschneider, F. (2020). Understanding physical activity and sedentary behaviour among preschool-aged children in Singapore: A mixed-methods approach. *BMJ Open*, *10*(4). https://doi.org/10.1136/bmjopen-2019-030606
85	Chen, Z. Y., Faride, S., Ong, H. S., Koshy, S., & Low, B. S. (2019). Influences of genetics, lifestyle and environment on obese and non-obese university students in Malaysia. *Journal of Public Health (Germany)*. https://doi.org/10.1007/s10389-019-01111-2
86	Chew, J., Tay, L., Lim, J. P., Leung, B. P., Yeo, A., Yew, S., Ding, Y. Y., & Lim, W. S. (2019). Serum Myostatin And Igf-1 As Gender-Specific Biomarkers Of Frailty And Low Muscle Mass. *The Journal of Nutrition, Health and Aging*, *23*(10), 979–986.
87	Chew, W. F., Masyita, M., Leong, P. P., Boo, N. Y., Zin, T., Choo, K. B., & Yap, S. F. (2014). Prevalence of obesity and its associated risk factors among Chinese adults in a Malaysian suburban village. *Singapore Medical Journal*, *55*(2), 84–91. https://doi.org/10.11622/smedj.2014020
88	Chew, W. F., Yap, S. F., Yasmin, A. M., Choo, K. B., Low, G. K. K., & Boo, N. Y. (2018). Risk factors associated with abdominal obesity in suburban adolescents from a Malaysian district. *Singapore Medical Journal*, *59*(2), 104–111. https://doi.org/10.11622/smedj.2017013
89	Chhoun, P., Ngin, C., Tuot, S., Pal, K., Steel, M., Dionisio, J., Pearson, H., Mburu, G., Brody, C., & Yi, S. (2017). Non-communicable diseases and related risk behaviors among men and women living with HIV in Cambodia: Findings from a cross-sectional study. *International Journal for Equity in Health*, *16*(1). https://doi.org/10.1186/s12939-017-0622-y
90	Chia, M. (2010). Pedometer-assessed physical activity of Singaporean youths. *Preventive Medicine*, *50*(5–6), 262–264. https://doi.org/10.1016/j.ypmed.2010.03.004
91	Chin, K. H., Sathyasurya, D. R., Saad, H. A., & Mohamed, H. J. B. J. (2013). Effect of ethnicity, dietary intake and physical activity on plasma adiponectin concentrations among Malaysian patients with type 2 diabetes mellitus. *International Journal of Endocrinology and Metabolism*, *11*(3), 167–174. https://doi.org/10.5812/ijem.8298
92	Chin, K. Y., Ima-Nirwana, S., Ibrahim, S., Mohamed, I. N., & Ngah, W. Z. W. (2014). Vitamin D status in Malaysian men and its associated factors. *Nutrients*, *6*(12), 5419–5433. https://doi.org/10.3390/nu6125419
93	Chin, K. Y., Low, N. Y., Dewiputri, W. I., & Ima-Nirwanaa, S. (2017). Factors associated with bone health in Malaysian middle-aged and elderly women assessed via quantitative ultrasound. *International Journal of Environmental Research and Public Health*, *14*(7). https://doi.org/10.3390/ijerph14070736
94	Chin, K. Y., Soelaiman, I. N., Mohamed, I. N., Ibrahim, S., & Wan Ngah, W. Z. (2012). The effects of age, physical activity level, and body anthropometry on calcaneal speed of sound value in men. *Archives of Osteoporosis*, *7*(1–2), 135–145. https://doi.org/10.1007/s11657-012-0091-2
95	Chirdkiatisak, M., Sranacharoenpong, K., Churak, P., & Praditsorn, P. (2019). Thai diabetes prevention education program: development and validation of the Thai physical activity questionnaire for at-risk people. *Journal of Public Health (Germany)*, *27*(5), 659–667. https://doi.org/10.1007/s10389-018-0989-2
96	Chong Seong, N. T., Yaakub, A., Jalil, R. A., Tirmandas VN, K., A/P Sandragasu, T., Noor, J. B. M., Husain, N. B., Mustari, Z. B., Hamid, S. A. A., Mt Saad, A. B., & AT, L.-S. (2019). Effect of physical activity on severity of primary angle closure glaucoma. *Therapeutic Advances in Ophthalmology*, *11*, 251584141986485. https://doi.org/10.1177/2515841419864855
97	Chongwatpol, P., & Gates, G. E. (2016). Differences in body dissatisfaction, weight-management practices and food choices of high-school students in the Bangkok metropolitan region by gender and school type. *Public Health Nutrition*, *19*(7), 1222–1232. https://doi.org/10.1017/S1368980016000100
98	Chu, A. H. Y., & Moy, F. M. (2013). Associations of occupational, transportation, household and leisure-time physical activity patterns with metabolic risk factors among middle-aged adults in a middle-income country. *Preventive Medicine*, *57*(SUPPL). https://doi.org/10.1016/j.ypmed.2012.12.011
99	Chu, A. H. Y., & Moy, F. M. (2014). Association between physical activity and metabolic syndrome among Malay adults in a developing country, Malaysia. *Journal of Science and Medicine in Sport*, *17*(2), 195–200. https://doi.org/10.1016/j.jsams.2013.04.003
100	Chu, A. H. Y., & Moy, F. M. (2015). Reliability and validity of the malay international physical activity questionnaire (IPAQ-M) among a Malay population in Malaysia. *Asia-Pacific Journal of Public Health*, *27*(2), NP2381–NP2389. https://doi.org/10.1177/1010539512444120
101	Chu, A. H. Y., Bernard, J. Y., Koh, D., & Müller-Riemenschneider, F. (2020). Accelerometer Profile of Physical Activity and Sedentary Behavior in a Multi-Ethnic Urban Asian Population. *Research Quarterly for Exercise and Sport*. https://doi.org/10.1080/02701367.2020.1734520
102	Chu, A. H. Y., Ng, S. H. X., Koh, D., Müller-Riemenschneider, F., & Brucki, S. (2015). Reliability and validity of the self- and interviewer-administered versions of the Global Physical Activity Questionnaire (GPAQ). *PLoS ONE*, *10*(9). https://doi.org/10.1371/journal.pone.0136944
103	Chu, A. H. Y., van Dam, R. M., Biddle, S. J. H., Tan, C. S., Koh, D., & Müller-Riemenschneider, F. (2018). Self-reported domain-specific and accelerometer-based physical activity and sedentary behaviour in relation to psychological distress among an urban Asian population. *International Journal of Behavioral Nutrition and Physical Activity*, *15*(1). https://doi.org/10.1186/s12966-018-0669-1
104	Chung, Q. E., Abdulrahman, S. A., Jamal Khan, M. K., Jahubar Sathik, H. B., & Rashid, A. (2018). The relationship between levels of physical activity and academic achievement among medical and health sciences students at cyberjaya university college of medical sciences. *Malaysian Journal of Medical Sciences*, *25*(5), 88–102. https://doi.org/10.21315/mjms2018.25.5.9
105	Chupanit, P., Muktabhant, B., & Schelp, F. P. (2018). Dietary patterns and their association with the components of metabolic syndrome: A cross-sectional study of adults from northeast Thailand. *F1000Research*, *7*, 905. https://doi.org/10.12688/f1000research.15075.1
106	Churangsarit, S., & Chongsuvivatwong, V. (2011). Spatial and social factors Associated with transportation and recreational physical activity among adults in Hat Yai city, Songkhla, Thailand. *Journal of Physical Activity and Health*, *8*(6), 758–765. https://doi.org/10.1123/jpah.8.6.758
107	Chusak, N. (2010). An eLearning Model in Prevention of Nutrition-related Diseases for Senior Working Adults. *2010 4th International Conference on Distance Learning and Education (ICDLE)*, 42–45.
108	Collins, A. E., Pakiz, B., & Rock, C. L. (2008). Factors associated with obesity in Indonesian adolescents. *International Journal of Pediatric Obesity*, *3*(1), 58–64. https://doi.org/10.1080/17477160701520132
109	Dang, A. K., Nguyen, L. H., Nguyen, A. Q., Tran, B. X., Tran, T. T., Latkin, C. A., Zhang, M. W. B., & Ho, R. C. M. (2018). Physical activity among HIV-positive patients receiving antiretroviral therapy in Hanoi and Nam Dinh, Vietnam: A cross-sectional study. *BMJ Open*, *8*(5). https://doi.org/10.1136/bmjopen-2017-020688
110	Dao, H. H., Do, Q. T., & Sakamoto, J. (2010). Increased frequency of metabolic syndrome among Vietnamese women with early rheumatoid arthritis: A cross-sectional study. *Arthritis Research and Therapy*, *12*(6). https://doi.org/10.1186/ar3203
111	Dao, H. H., Do, Q. T., & Sakamoto, J. (2011). Abnormal body composition phenotypes in Vietnamese women with early rheumatoid arthritis. *Rheumatology*, *50*(7), 1250–1258. https://doi.org/10.1093/rheumatology/ker004
112	Dao-Tran, T. H., & Seib, C. (2018). Prevalence and correlates of sleep disturbance among older women in Vietnam. *Journal of Clinical Nursing*, *27*(17–18), 3307–3313. https://doi.org/10.1111/jocn.14080
113	Dao-Tran, T. H., Anderson, D., & Seib, C. (2017). How life stressors influence modifiable lifestyle factors, depressive symptoms, and physical and mental health among Vietnamese older women? *BMC Psychiatry*, *17*(1). https://doi.org/10.1186/s12888-017-1395-y
114	Daud, N., Ongsang, S., Tengah, A., Abdul Rahman, H., & Abdul-Mumin, K. (2019). Physical activities among medical–surgical nurses: a descriptive cross-sectional study. *Journal of Public Health (Germany)*. https://doi.org/10.1007/s10389-019-01125-w
115	de Guzman, A. B., Lacampuenga, P. E. U., & Lagunsad, A. P. V. (2015). Examining the Structural Relationship of Physical Activity, Cognition, Fear of Falling, and Mobility Limitation of Filipino in Nursing Homes. *Educational Gerontology*, *41*(7), 527–542. https://doi.org/10.1080/03601277.2014.986398
116	de Guzman, A., Lago, B., Nieves, R., Parungao Ma, N., & Urquiza, K. (2017). Nutritional Quality of Meals, Food Intake, Physical Activity and Length of Stay Predict Nutritional Status of Institutionalised Elderly Filipinos. *Malaysian Journal of Nutrition*, *23*(3), 319–327.
117	Deurenberg-Yap, M., Chew, S., Lin, V., Tan, B., van Staveren, W., & Deurenberg, P. (2001). PAPER Relationships between indices of obesity and its co-morbidities in multi-ethnic Singapore. *International Journal of Obesity, 25*.
118	Deurenberg-Yap, M., Li, T., Tan, W. L., Staveren, W. A. va., Chew, S. K., & Deurenberg, P. (2001). Can dietary factors explain differences in serum cholesterol profiles among different ethnic groups (Chinese, Malays and Indians) in Singapore? *Asia Pacific Journal of Clinical Nutrition*, *10*(1), 39–45. https://doi.org/10.1046/j.1440-6047.2001.00202.x
119	Dharmastuti, D. P., Agni, A. N., Widyaputri, F., Pawiroranu, S., Sofro, Z. M., Wardhana, F. S., Haryanto, S., Widayanti, T. W., Kotha, S., Gupta, P., & Sasongko, M. B. (2018). Associations of Physical Activity and Sedentary Behaviour with Vision-Threatening Diabetic Retinopathy in Indonesian Population with Type 2 Diabetes Mellitus: Jogjakarta Eye Diabetic Study in the Community (JOGED.COM). *Ophthalmic Epidemiology*, *25*(2), 113–119. https://doi.org/10.1080/09286586.2017.1367410
120	Diana, D., Basuki, B., & Kurniarobbi, J. (2009). Low physical activity work-related and other risk factors increased the risk of poor physical fitness in cement workers. *Medical Journal of Indonesia*, *18*(3), 203–207. https://doi.org/10.13181/mji.v18i3.362
121	Dogara Andrew, V., Johari Mohd Yusof, M., & Abu Kasim, J. (2020). Relationship Between Physical Activity in Urban Green Space and Dietary Patterns among Obese Children in Kuala Lumpur, Malaysia. *Pertanika Journal of Social Sciences & Humanities*, *28*(1), 633–645.
122	Dominic, N. A., & Kean Heng, L. (2018). Perceived Barriers to Exercise in Women with Gestational Diabetes Mellitus. *International Medical Journal Malaysia*, *17*(3). https://www.researchgate.net/publication/329371064
123	Duc Son, L. E. N. T., Kusama, K., Hung, N. T. K., Loan, T. T. H., Van Chuyen, N., Kunii, D., Sakai, T., & Yamamoto, S. (2004). Prevalence and risk factors for diabetes in Ho Chi Minh City, Vietnam. *Diabetic Medicine*, *21*(4), 371–376. https://doi.org/10.1111/j.1464-5491.2004.01159.x
124	Eaglehouse, Y. L., Koh, W. P., Wang, R., Aizhen, J., Yuan, J. M., & Butler, L. M. (2017). Physical activity, sedentary time, and risk of colorectal cancer: The Singapore Chinese Health Study. *European Journal of Cancer Prevention*, *26*(6), 469–475. https://doi.org/10.1097/CEJ.0000000000000369
125	Emi, N. A., Gan, W. Y., Mohd Shariff, Z., Anuar Zaini, A., Shamsuddin, N. H., Appukutty, M., & Appannah, G. (2020). Associations of an empirical dietary pattern with cardiometabolic risk factors in Malaysian adolescents. *Nutrition and Metabolism*, *17*(1). https://doi.org/10.1186/s12986-020-00447-x
126	Ernawati, M. G., Kusdhany, L. S., Agustin, D., Iskandar, H. B., & Rahardjo, T. B. (2017). A Mandibular Bone Density Index for Prediction of Jaw Bone Osteoporosis in Men. *Journal of International Dental and Medical Research, 10*. http://www.ektodermaldisplazi.com/journal.htm
127	Esfahani, M., Musa, G., & Khoo, S. (2017). The Influence of Spirituality and Physical Activity Level on Responsible Behaviour and Mountaineering Satisfaction on Mount Kinabalu, Borneo. *Current Issues in Asian Tourism*, *20*(11), 1162–1185. https://www.researchgate.net/publication/269144682
128	Ethisan, P., Chapman, R., & Kumar, R. (2015). Effectiveness Of Group-Mediated Lifestyle Physical Activity (Glpa) Program For Health Benefit In Physical Activity Among Elderly People At Rural Thailand. In *Journal of Ayub Medical College Abbottabad, 27*(2). http://www.jamc.ayubmed.edu.pk
129	Ethisan, P., Somrongthong, R., Ahmed, J., Kumar, R., & Chapman, R. S. (2017). Factors related to physical activity among the elderly population in rural Thailand. *Journal of Primary Care and Community Health*, *8*(2), 71–76. https://doi.org/10.1177/2150131916675899
130	Fadhilah, A., & Allenidekania, A. (2019). The Relationship between Activity Level and Fatigue in Indonesian Children with Acute Lymphocytic Leukemia in the Home Setting. *Comprehensive Child and Adolescent Nursing*, *42*(sup1), 47–55. https://doi.org/10.1080/24694193.2019.1577925
131	Fadhli, M., Fauzi, M., & Yusoff, H. M. (2019). Prevalence and Determinants of Pre-Hypertension among Undergraduate Preclinical Medical Students in Central Malaysia. *International Medical Journal Malaysia*. https://www.researchgate.net/publication/337783343
132	Fajarini, I. A., & Sartika, R. A. D. (2019). Obesity as a common type-2 diabetes comorbidity: Eating behaviors and other determinants in Jakarta, Indonesia. *Kesmas*, *13*(4), 157–163. https://doi.org/10.21109/kesmas.v13i4.2483
133	Farooqui, M. A., Tan, Y. T., Bilger, M., & Finkelstein, E. A. (2014). Effects of financial incentives on motivating physical activity among older adults: Results from a discrete choice experiment. *BMC Public Health*, *14*(1). https://doi.org/10.1186/1471-2458-14-141
134	Fatin, N., Rivan, M., Shahar, S., Haron, H., Ambak, R., & Othman, F. (2018). Association between intake of soy isoflavones and blood pressure among urban and rural Malaysian adults. *Malaysian Journal of Nutrition,* 24(3).
135	Febriani, D., & Febriani, B. (2018). The Effect of Lifestyle on Hypercholesterolemia. *The Open Public Health Journal*, *11*(1), 526–532. https://doi.org/10.2174/1874944501811010526
136	Febriani, D., & Sudarti, T. (2019). Fast food as drivers for overweight and obesity among urban school children at Jakarta, Indonesia. *Jurnal Gizi Dan Pangan*, *14*(2), 99–106. https://doi.org/10.25182/jgp.2019.14.2.99-106
137	Finkelstein, E. A., Sahasranaman, A., John, G., Haaland, B. A., Bilger, M., Sloan, R. A., Nang, E. E. K., & Evenson, K. R. (2015). Design and baseline characteristics of participants in the TRial of Economic Incentives to Promote Physical Activity (TRIPPA): A randomized controlled trial of a six month pedometer program with financial incentives. *Contemporary Clinical Trials*, *41*, 238–247. https://doi.org/10.1016/j.cct.2015.01.020
138	Finkelstein, E. A., Tan, Y. T., Malhotra, R., Lee, C. F., Goh, S. S., & Saw, S. M. (2013). A cluster randomized controlled trial of an incentive-based outdoor physical activity program. *Journal of Pediatrics*, *163*(1). https://doi.org/10.1016/j.jpeds.2013.01.009
139	Firouzi, S., Nisak, B., Yusof, M., Yusof Barakatun-Nisak, M., & Azmi, K. N. (2015). Nutritional status, glycemic control and its associated risk factors among a sample of type 2 diabetic individuals, a pilot study. *Journal of research in medical sciences*. https://www.researchgate.net/publication/273464034
140	Firouzi, S., Poh, B. K., Ismail, M. N., & Sadeghilar, A. (2014). Sleep habits, food intake, and physical activity levels in normal and overweight and obese Malaysian children. *Obesity Research and Clinical Practice*, *8*(1). https://doi.org/10.1016/j.orcp.2012.12.001
141	Florentino, R. F., Villavieja, G. M., & Lana, R. D. (2002). Dietary and physical activity patterns of 8- to 10- year-old urban schoolchildren in Manila, Philippines. *Food and Nutrition Bulletin*, *23*(3), 267–273. https://doi.org/10.1177/156482650202300306
142	Fong, C. Y., Kong, A. N., Poh, B. K., Mohamed, A. R., Khoo, T. B., Ng, R. L., Noordin, M., Nadarajaw, T., & Ong, L. C. (2016). Vitamin D deficiency and its risk factors in Malaysian children with epilepsy. *Epilepsia*, *57*(8), 1271–1279. https://doi.org/10.1111/epi.13443
143	Foo, L. H., Khor, G. L., Tee, E. S., & Dhanaraj, P. (2004). Determinants of iron status in Malaysian adolescents from a rural community. *International Journal of Food Sciences and Nutrition*, *55*(6), 517–525. https://doi.org/10.1080/09637480400015786
144	Fung, F. Y., Koh, Y. L. E., Malhotra, R., Ostbye, T., Lee, P. Y., Shariff Ghazali, S., & Tan, N. C. (2019). Prevalence of and factors associated with sarcopenia among multi-ethnic ambulatory older Asians with type 2 diabetes mellitus in a primary care setting. *BMC Geriatrics*, *19*(1). https://doi.org/10.1186/s12877-019-1137-8
145	Goh, Z. Y., Lee, P. Y., & Lai, Y. F. (2019). Patterns of fruit and vegetable intake and physical activity among community-ambulant patients in Singapore: A cross-sectional study. *Proceedings of Singapore Healthcare*, *28*(1), 10–18. https://doi.org/10.1177/2010105818769682
146	Gonzalez-Suarez, C. B., & Grimmer-Somers, K. (2009). Physical activity pattern of prepubescent filipino school children during school days: Research article. *Journal of School Health*, *79*(7), 304–311. https://doi.org/10.1111/j.1746-1561.2009.00414.x
147	Grover, S., Sinha, D. N., Gupta, S., Gupta, P. C., & Mehrotra, R. (2019). The changing face of risk factors for non-communicable disease in Myanmar: Findings from the 2009 and 2014 WHO STEP Surveys. *Journal of Public Health (United Kingdom)*, *41*(4), 750–756. https://doi.org/10.1093/pubmed/fdy176
148	Guthold, R., Cowan, M. J., Autenrieth, C. S., Kann, L., & Riley, L. M. (2010). Physical Activity and Sedentary Behavior Among Schoolchildren: A 34-Country Comparison. *Journal of Pediatrics*, *157*(1). https://doi.org/10.1016/j.jpeds.2010.01.019
149	Guthold, R., Stevens, G. A., Riley, L. M., & Bull, F. C. (2018). Worldwide trends in insufficient physical activity from 2001 to 2016: a pooled analysis of 358 population-based surveys with 1·9 million participants. *The Lancet Global Health*, *6*(10), e1077–e1086. https://doi.org/10.1016/S2214-109X(18)30357-7
150	Hadju, V., Stephenson, L., Mohammed, H., Bowman, D., & Parker, R. (1998). Improvements of growth, appetite, and physical activity in helminth-infected schoolboys 6 months after single dose of albendazole. *Asia Pacific Journal of Clinical Nutrition, 7*(2).
151	Handayani, O. W. K., Wiranti, I., Raharjo, B. B., & Nugroho, E. (2019). The Reproduction Health Behavior of High School Teenagers in Semarang, Indonesia. *The Open Public Health Journal*, *12*(1), 309–314. https://doi.org/10.2174/1874944501912010309
152	Harahap, H., Sandjaja, S., Soekatri, M., Khouw, I., & Deurenberg, P. (2018). Association of energy intake and physical activity with overweight among Indonesian children 6-12 years of age. *Asia Pacific Journal of Clinical Nutrition*, *27*(1), 211–216. https://doi.org/10.6133/apjcn.032017.05
153	Harnirattisai, T., & Johnson, R. A. (2005). Effectiveness of a behavioral change intervention in Thai elders after knee replacement. *Nursing Research*, *54*(2), 97–107. https://doi.org/10.1097/00006199-200503000-00004
154	Hartono, R. K., Hamid, S. A., & Hafizurrachman, M. (2019). Do the number of cigarettes smokes per day contribute to the incident of malignant cancer? *Asian Pacific Journal of Cancer Prevention*, *20*(5), 1403–1408. https://doi.org/10.31557/APJCP.2019.20.5.1403
155	Hasan, N., Hadju, V., Jafar, N., & Thaha, R. M. (2019). Prevalence of Metabolic Syndrome (MetS) and Determinants Among Obese Teachers in Makassar, Indonesia. *Iium Medical Journal Malaysia*, *18*(2).
156	Hashim, H. A., Golok, F., & Ali, R. (2011). Profiles of exercise motivation, physical activity, exercise habit, and academic performance in Malaysian adolescents: A cluster analysis. *International Journal of Collaborative Research on Internal Medicine & Public Health*, *3*(6), 416–428. http://www.iomcworld.com/ijcrimph/ArticleURL:http://iomcworld.com/ijcrimph/ijcrimph-v03-n06-01.htm
157	Haya, M., Mexitalia, M., & Margawati, A. (2016). Effect of maternal health education on physical activity and body mass index of overweight children. *Paediatrica Indonesiana, 56*(2).
158	Hidrus, A., Kueh, Y. C., Norsaádah, B., Chang, Y. K., Hung, T. M., Naing, N. N., & Kuan, G. (2020). Effects of brain breaks videos on the motives for the physical activity of Malaysians with type-2 diabetes mellitus. *International Journal of Environmental Research and Public Health*, *17*(7). https://doi.org/10.3390/ijerph17072507
159	Ho, A. H. Y., Ma, S. H. X., Ho, M. H. R., Pang, J. S. M., Ortega, E., & Bajpai, R. (2019). Arts for ageing well: A propensity score matching analysis of the effects of arts engagements on holistic well-being among older Asian adults above 50 years of age. *BMJ Open*, *9*(11). https://doi.org/10.1136/bmjopen-2019-029555
160	Ho, C. S., Jin, A., Nyunt, M. S. Z., Feng, L., & Ng, T. P. (2016). Mortality rates in major and subthreshold depression: 10-year follow-up of a Singaporean population cohort of older adults. *Postgraduate Medicine*, *128*(7), 642–647. https://doi.org/10.1080/00325481.2016.1221319
161	Ho, S. E., Ting, J. A. J., Lee, L. C., & Wong, P. F. (2016). Information Needs in Relation to Physical Activity among Angina Patients before Percutaneous Coronary Intervention (PCI) at a Private Hospital in Penang, Malaysia. *Journal of Krishna Institute of Medical Sciences University*, *5*(4), 18–23.
162	Hong, T. K., Trang, N. H. H. D., Dibley, M. J., Sibbritt, D. W., Binh, P. N. T., & Hanh, T. T. M. (2010). Factors associated with adolescent overweight/obesity in Ho Chi Minh city. *International Journal of Pediatric Obesity*, *5*(5), 396–403. https://doi.org/10.3109/17477160903540735
163	Hong, T. K., Trang, N. H. H. D., van der Ploeg, H. P., Hardy, L. L., & Dibley, M. J. (2012). Validity and reliability of a physical activity questionnaire for Vietnamese adolescents. *International Journal of Behavioral Nutrition and Physical Activity*, *9*. https://doi.org/10.1186/1479-5868-9-93
164	Htay, H., Po, L., & Mya-Tu, M. (1978). Habitual physical activity of rural burmese women. *Ergonomics*, *21*(4), 239–245. https://doi.org/10.1080/00140137808931720
165	Htet, A. S., Bjertness, M. B., Oo, W. M., Kjøllesdal, M. K., Sherpa, L. Y., Zaw, K. K., Ko, K., Stigum, H., Meyer, H. E., & Bjertness, E. (2017). Changes in prevalence, awareness, treatment and control of hypertension from 2004 to 2014 among 25-74-year-old citizens in the Yangon Region, Myanmar. *BMC Public Health*, *17*(1). https://doi.org/10.1186/s12889-017-4870-y
166	Huei Phing, C., Abu Saad, H., Barakatun Nisak, M. Y., & Mohd Nasir, M. T. (2017). Effectiveness of physical activity intervention among government employees with metabolic syndrome. *Journal of Exercise Science and Fitness*, *15*(2), 55–62. https://doi.org/10.1016/j.jesf.2017.07.003
167	Hughes, K., Leong, W. P., Sothy, S. P., Lun, K. C., & B Yeo, P. P. (1993). Relationships between Cigarette Smoking, Blood Pressure and Serum Lipids in the Singapore General Population. *International Journal of Epidemiology, 22*(4).
168	Hui Tee, J. Y., Gan, W. Y., Tan, K. A., & Chin, Y. S. (2018). Obesity and unhealthy lifestyle associated with poor executive function among Malaysian adolescents. *PLoS ONE*, *13*(4). https://doi.org/10.1371/journal.pone.0195934
169	Huynh, D. T. T., Estorninos, E., Capeding, R. Z., Oliver, J. S., Low, Y. L., & Rosales, F. J. (2015). Longitudinal growth and health outcomes in nutritionally at-risk children who received long-term nutritional intervention. *Journal of Human Nutrition and Dietetics*, *28*(6), 623–635. https://doi.org/10.1111/jhn.12306
170	Ibrahim, N., Moy, F. M., Awalludin, I. A. N., Ali, Z. M., & Ismail, I. S. (2016). Effects of a community-based healthy lifestyle intervention program (Co-HELP) among adults with prediabetes in a developing country: A quasi-experimental study. *PLoS ONE*, *11*(12). https://doi.org/10.1371/journal.pone.0167123
171	Ibrahim, N., Moy, F. M., Awalludin, I. A. N., Ali, Z., & Ismail, I. S. (2014). The health-related quality of life among pre-diabetics and its association with body mass index and physical activity in a semi-urban community in Malaysia—A cross sectional study. *BMC Public Health*, *14*(1), 298. https://doi.org/10.1186/1471-2458-14-298
172	Ibrahim, S., Karim, N. A., Oon, N. L., & Ngah, W. Z. W. (2013). Perceived physical activity barriers related to body weight status and sociodemographic factors among Malaysian men in Klang Valley. *BMC Public Health*, *13*(1), 275. https://doi.org/10.1186/1471-2458-13-275
173	Imanda, A., Martini, S., & Artanti, K. D. (2019). Post hypertension and stroke: A case control study. *Kesmas*, *13*(4), 164–168. https://doi.org/10.21109/kesmas.v13i4.2261
174	In-Iw Md, S., Manaboriboon, B., & Chomchai, C. (2010). A Comparison of Body-Image Perception, Health Outlook and Eating Behavior in Mildly Obese Versus Moderately-to-Severely Obese Adolescents. *Journal of the Medical Association of Thailand,* 93(4).
175	Insawang, T., Selmi, C., Cha’on, U., Pethlert, S., Yongvanit, P., Areejitranusorn, P., Boonsiri, P., Khampitak, T., Tangrassameeprasert, R., Pinitsoontorn, C., Prasongwattana, V., Gershwin, M. E., & Hammock, B. D. (2012). Monosodium glutamate (MSG) intake is associated with the prevalence of metabolic syndrome in a rural Thai population. *Nutrition and Metabolism*, *9*. https://doi.org/10.1186/1743-7075-9-50
176	Intana, J., Khamthana, P., Siramaneerat, I., & Chaowilai, C. (2019). The Effect of Physical Exercise Program in Patients with Type 2 Diabetes Mellitus in Ratchaburi Province, Thailand. *The Open Public Health Journal*, *12*(1), 369–373. https://doi.org/10.2174/1874944501912010369
177	Inthachai, T., Demekul, K., Phonsatsadee, N., Puttitommagool, P., & Boonyachart, N. (2019). Effects of physical activity and smoking on cardio-ankle vascular index, respiratory muscle strength, and exercise performance in early normal weight adulthood: A cross-sectional study. *Journal of Exercise Rehabilitation*, *15*(6), 804–810. https://doi.org/10.12965/jer.1938676.338
178	Iqbal, S. P., Ramadas, A., Fatt, Q. K., Shin, H. L., Onn, W. Y., & Kadir, K. A. (2020). Relationship of sociodemographic and lifestyle factors and diet habits with metabolic syndrome (MetS) among three ethnic groups of the Malaysian population. *PLoS ONE*, *15*(3). https://doi.org/10.1371/journal.pone.0224054
179	Isaura, E. R., Chen, Y. C., & Yang, S. H. (2018). Pathways from food consumption score to cardiovascular disease: A seven-year follow-up study of Indonesian adults. *International Journal of Environmental Research and Public Health*, *15*(8). https://doi.org/10.3390/ijerph15081567
180	Ismail, M. N., Chee, S. S., Nawawi, H., Yusoff, K., Lim, T. O., & T James, W. P. (2002). Obesity in Malaysia obesity reviews. *Obesity Reviews*, *3*(3), 203–208.
181	Ismail, N., Hairi, F., Choo, W. Y., Hairi, N. N., Peramalah, D., & Bulgiba, A. (2015). The Physical Activity Scale for the Elderly (PASE): Validity and reliability among community-dwelling older adults in Malaysia. *Asia-Pacific Journal of Public Health*, *27*, 62S-72S. https://doi.org/10.1177/1010539515590179
182	Iswarawanti, D., Schultink, W., Rumawas, J., & Lukito, W. (1996). Body composition and physical activity patterns of Indonesian elderly with low body mass index. *Asian Pacific Journal of Cancer Prevention*, *5*(4), 222–225.
183	Iwasaki, M., Kimura, Y., Sasiwongsaroj, K., Kettratad-Pruksapong, M., Suksudaj, S., Ishimoto, Y., Chang, N. Y., Sakamoto, R., Matsubayashi, K., Songpaisan, Y., & Miyazaki, H. (2018). Association between objectively measured chewing ability and frailty: A cross-sectional study in central Thailand. *Geriatrics and Gerontology International*, *18*(6), 860–866. https://doi.org/10.1111/ggi.13264
184	Izham, M., Ibrahim, M., Hassali, M. A., Shafie, A. A., & Othman, A. (2014). Outcomes Of Cardiovascular Risk Factors Screening Programme Among Employees Of A Malaysian Public University. *Journal of Clinical and Diagnostic Research*, *4*(2), 2208–2216. https://www.researchgate.net/publication/289299688
185	Jafar, T. H., Jin, A., Koh, W. P., Yuan, J. M., & Chow, K. Y. (2015). Physical activity and risk of end-stage kidney disease in the Singapore Chinese Health Study. *Nephrology*, *20*(2), 61–67. https://doi.org/10.1111/nep.12355
186	Jafar, T. H., Tan, N. C., Allen, J. C., Pradhan, S. S., Goh, P., Tavajoh, S., Keng, F. M., & Chan, J. (2016). Management of hypertension and multiple risk factors to enhance cardiovascular health—A feasibility study in Singapore polyclinics. *BMC Health Services Research*, *16*(1). https://doi.org/10.1186/s12913-016-1491-6
187	Jajat, Sultoni, K., & Suherman, A. (2017). Barriers to Physical Activity on University Student. *IOP Conference Series: Materials Science and Engineering*, *180*(1). https://doi.org/10.1088/1757-899X/180/1/012210
188	Jalayondeja, C., Jalayondeja, W., Suttiwong, J., Sullivan, P., & Nilanthi, D. (2016). Physical Activity, Self-Esteem, And Quality Of Life Among People With Physical Disability. *The Southeast Asian Journal of Tropical Medicine and Public Health*, *47*(3), 546–558. https://www.researchgate.net/publication/306979961
189	Jamil, A. T., & Singh, R. (2015). Non-leisure time physical activity for adult Malaysian and determinant factors. *Malaysian Journal of Public Health Medicine, 15*(2), 84-93. https://www.researchgate.net/publication/281490169
190	Jamil, A. T., Rosli, N. M., Ismail, A., Idris, I. B., & Omar, A. (2016). Prevalence And Risk Factors For Sedentary Behavior Among Malaysian Adults. *Malaysian Journal of Public Health Medicine, 16*(3).
191	Jamil, N. A., Jia Ling, C., Md Ibrahim, H. I., Hamzaid, N. H., & Kok Yong, C. (2020). Nutritional and bone health status in young men with mild-to-moderate intellectual disability and without intellectual disability residing in community setting in Malaysia. *Journal of Applied Research in Intellectual Disabilities*, *33*(3), 632–639. https://doi.org/10.1111/jar.12708
192	Jia Jun, N., Shazmin Kamarudin, K., Ali, A., & Salihah Zakaria, N. (2020). Motivators and Barriers of Physical Activity among Private Office Workers in Selangor. *Malaysian Journal of Medicine and Health Science, 16*(1).
193	Jiamjarasrangsi, W., Attavorrarat, S., Navicharern, R., Aekplakorn, W., & Keesukphan, P. (2014). Assessment of 5-year system-wide type 2 diabetes control measures in a Southeast Asian metropolis. *Asian Biomedicine*, *8*(1), 75–82. https://doi.org/10.5372/1905-7415.0801.264
194	Jiamjarasrangsi, W., Navicharern Rn, R., Bpharm, S. A., Manit Rn, A., Aekplakorn, W., & Keesukphan, P. (2017). Identification of Potential Leverage Points for Self-Management Support Intervention in Thais with Type 2 Diabetes. *Journal of the Medical Association of Thailand, 100*(3).
195	Jitnarin, N., & Haddock, C. K. (2008). The Relationship between Smoking, BMI, Physical Activity, and Dietary Intake among Thai Adults in Central Thailand Occupational health and epidemiology View project Kansas City Built Environment and Health Study View project. *Journal of the Medical Association of Thailand = Chotmaihet thangphaet*. https://www.researchgate.net/publication/23304302
196	Johari, S. M., Nordin, N. J., Sahar, M. A., Sulaiman, A. H., Shahar, S., Teng, N. I. M. F., Amin, N. A., & Saudi, A. S. M. (2018). High body fat percentage among adult women in Malaysia: the role of lifestyle. *Journal of Fundamental and Applied Sciences*, *9*(4S), 905. https://doi.org/10.4314/jfas.v9i4s.52
197	Jordan, S., Lim, L., Berecki-Gisolf, J., Bain, C., Seubsman, S. A., Sleigh, A., Banks, E., Chokhanapitak, J., Churewong, C., Hounthasarn, S., Khamman, S., Pandee, D., Pangsap, S., Prapamontol, T., Puengson, J., Sangrattanakul, Y., Somboonsook, B., Sripaiboonkij, N., Somsamai, P., … Yiengprugsawan, V. (2013). Body mass index, physical activity, and fracture among young adults: Longitudinal results from the thai cohort study. *Journal of Epidemiology*, *23*(6), 435–442. https://doi.org/10.2188/jea.JE20120215
198	Juliana, N., Shahar, S., Sahar, M. A., Ghazali, A. R., Manaf, Z. A., & Noah, R. M. (2017). “Her shape” intervention programme for obese women with high breast adiposity. *Asia Pacific Journal of Clinical Nutrition*, *26*(2), 278–286. https://doi.org/10.6133/apjcn.122015.05
199	Kamso, S. (2007). Body mass index, total cholesterol, and ratio total to HDL cholesterol were determinants of metabolic syndrome in the Indonesian elderly. *Medical Journal of Indonesia*, *16*(3), 195–200. https://doi.org/10.13181/mji.v16i3.276
200	Kandiah, N., Lekhraj, R., Paranjothy, S., & Kaur Gill, A. (1980). A community based study on the epidemiology of hypertension in Selangor. *The Medical Journal of Malaysia*, *34*(3). https://www.researchgate.net/publication/15785762
201	Kang Cheah, Y., Azahadi, M., Nooi Phang, S. C., & Hazilah Abd Manaf, N. (2017). The Income Elasticity of Participation in Physical Activity: Evidence from Malaysia. *International Journal of Economics and Management, 11*(2).
202	Karintrakul, S., & Angkatavanich, J. (2017). A randomized controlled trial of an individualized nutrition counseling program matched with a transtheoretical model for overweight and obese females in Thailand. *Nutrition Research and Practice*, *11*(4), 319–326. https://doi.org/10.4162/nrp.2017.11.4.319
203	Kaur, G., Tee, G. H., Ariaratnam, S., Krishnapillai, A. S., & China, K. (2013). Depression, anxiety and stress symptoms among diabetics in Malaysia: A cross sectional study in an urban primary care setting. *BMC Family Practice*, *14*. https://doi.org/10.1186/1471-2296-14-69
204	Kaur, J., Kaur, G., Ho, B. K., Yao, W. K., Salleh, M., & Lim, K. H. (2015). Predictors of physical inactivity among elderly Malaysians: Recommendations for policy planning. *Asia-Pacific Journal of Public Health*, *27*(3), 314–322. https://doi.org/10.1177/1010539513517257
205	Kaur, S., & Singh, N. (2014). Prevalence of metabolic syndrome and its relation to body composition in Chinese elderly. *Asian Journal of Gerontology and Geriatrics, 9*(1). https://www.researchgate.net/publication/301635355
206	Kaur, S., Ng, C. M., Badon, S. E., Jalil, R. A., Maykanathan, D., Yim, H. S., & Jan Mohamed, H. J. (2019). Risk factors for low birth weight among rural and urban Malaysian women. *BMC Public Health*, *19*. https://doi.org/10.1186/s12889-019-6864-4
207	Khaing Nang, E. E., Khoo, E. Y. H., Salim, A., Tai, E. S., Lee, J., & Van Dam, R. M. (2010). Patterns of physical activity in different domains and implications for intervention in a multi-ethnic Asian population: A cross-sectional study. *BMC Public Health*, *10*. https://doi.org/10.1186/1471-2458-10-644
208	Kho, W. F. G., Cheah, W. L., & Hazmi, H. (2018). Elevated blood pressure and its predictors among secondary school students in Sarawak: A cross-sectional study. *Central European Journal of Public Health*, *26*(1), 16–21. https://doi.org/10.21101/cejph.a5186
209	Khruakhorn, S., Sritipsukho, P., Siripakarn, Y., & Vachalathiti, R. (2010). Prevalence and Risk Factors of Low Back Pain among the University Staff. *Article in Journal of the Medical Association of Thailand = Chotmaihet thangphaet, 93*(Suppl. 7), S142-148.
210	Kidokoro, T., Suzuki, K., Naito, H., Balasekaran, G., Song, J. K., Park, S. Y., Liou, Y. M., Lu, D., Poh, B. K., Kijboonchoo, K., & Hui, S. S. chuen. (2019). Moderate-to-vigorous physical activity attenuates the detrimental effects of television viewing on the cardiorespiratory fitness in Asian adolescents: The Asia-fit study. *BMC Public Health*, *19*(1). https://doi.org/10.1186/s12889-019-8079-0
211	Kjøllesdal, M., Htet, A. S., Stigum, H., Hla, N. Y., Hlaing, H. H., Khaine, E. K., Khaing, W., Khant, A. K., Khin, N. O. K., Mauk, K. K. A., Moe, E. E., Moe, H., Mon, K. K., Mya, K. S., Myint, C. K., Myint, C. Y., Myint, M. M., Myint, O., New, A. A., … Bjertness, E. (2016). Consumption of fruits and vegetables and associations with risk factors for non-communicable diseases in the Yangon region of Myanmar: a cross-sectional study. *BMJ Open*, *6*(8), e011649. https://doi.org/10.1136/bmjopen-2016-011649
212	Klongthalay, S., & Suriyaprom, K. (2020). Increased Uric Acid and Life Style Factors Associated with Metabolic Syndrome in Thais. *Ethiopian Journal of Health Sciences*, *30*(2), 199–208. https://doi.org/10.4314/ejhs.v30i2.7
213	Koh, A. S., Gao, F., Tan, R. S., Zhong, L., Leng, S., Zhao, X., Fridianto, K. T., Ching, J., Lee, S. Y., Keng, B. M. H., Yeo, T. J., Tan, S. Y., Tan, H. C., Lim, C. T., Koh, W. P., & Kovalik, J. P. (2018). Metabolomic correlates of aerobic capacity among elderly adults. *Clinical Cardiology*, *41*(10), 1300–1307. https://doi.org/10.1002/clc.23016
214	Kok, W. H. M., Poh, B. K., Wee, L. H., Devanthini, G., & Ruzita, A. T. (2018). Juara Sihat: assessing the sustained impact of a school-based obesity intervention. *The Medical Journal of Malaysia*, *73*(2), 100–105.
215	Ko-Ko-Zaw, Tint-Swe-Latt, Phyu-Phyu-Aung, Thein-Gi-Thwin, & Tin-Khine-Myint. (2011). Prevalence of hypertension and its associated factors in the adult population in Yangon Division, Myanmar. *Asia-Pacific Journal of Public Health*, *23*(4), 496–506. https://doi.org/10.1177/1010539509349147
216	Konharn, K., Santos, M. P., & Ribeiro, J. C. (2014). Socioeconomic status and objectively measured physical activity in thai adolescents. *Journal of Physical Activity and Health*, *11*(4), 712–720. https://doi.org/10.1123/jpah.2011-0424
217	Krishnan, A., Ekowati, R., Baridalyne, N., Kusumawardani, N., Suhardi, Kapoor, S. K., & Leowski, J. (2011). Evaluation of community-based interventions for non-communicable diseases: Experiences from India and Indonesia. *Health Promotion International*, *26*(3), 276–289. https://doi.org/10.1093/heapro/daq067
218	Kristina, S. A., Endarti, D., Widayanti, A. W., & Widiastuti, M. (2015). Health-related Quality of Life Among Smokers in Yogyakarta Province, Indonesia. *International Journal of Pharmaceutical and Clinical Research*, *8*(1), 95–99. www.ijpcr.com
219	Kruavit, A., Chailurkit, L. O., Thakkinstian, A., Sriphrapradang, C., & Rajatanavin, R. (2012). Prevalence of Vitamin D insufficiency and low bone mineral density in elderly Thai nursing home residents. *BMC Geriatrics*, *12*. https://doi.org/10.1186/1471-2318-12-49
220	Kueh, Y. C., Abdullah, N., Chin, N.-S., Morris, T., & Kuan, G. (2019). Motivation for physical activity among preadolescent Malay students in Kelantan, Malaysia. *Pertanika Journal of Social Science and Humanities*, *1*(27), 675–683. https://www.researchgate.net/publication/331988542
221	Kueh, Y. C., Abdullah, N., Kuan, G., Morris, T., & Naing, N. N. (2018). Testing measurement and factor structure invariance of the Physical Activity and Leisure Motivation scale for youth across gender. *Frontiers in Psychology*, *9*(JUL). https://doi.org/10.3389/fpsyg.2018.01096
222	Kuramasuwan, B., Howteerakul, N., Suwannapong, N., & Rawdaree, P. (2013). Diabetes, impaired fasting glucose, daily life activities, food and beverage consumption among Buddhist monks in Chanthaburi Province, Thailand. *International Journal of Diabetes in Developing Countries*, *33*(1), 23–28. https://doi.org/10.1007/s13410-012-0094-y
223	Kusmana, D. (2001). The profile of physical activity and coronary risk factors in monica jakarta survey. *Medical Journal of Indonesia*, *10*(1), 34–41. https://doi.org/10.13181/mji.v10i1.7
224	Kusmana, D. (2002). The influence of smoking cessation, regular physical exercise and/or physical activity on survival: a 13 years cohort study of the Indonesian population in Jakarta. *Medical Journal of Indonesia, 11*.
225	Kuzawa, C. W., Adair, L. S., Avila, J. L., Cadungog, J. H. C., & Le, N. A. (2003). Atherogenic lipid profiles in Filipino adolescents with low body mass index and low dietary fat intake. *American Journal of Human Biology*, *15*(5), 688–696. https://doi.org/10.1002/ajhb.10200
226	Kwanbunjan, K., Chumpathat, N., Phosat, C., Uttamachai, C., & Panprathip, P. (2018). Association between waist circumference at two measurement sites and indicators of metabolic syndrome and cardiovascular disease among Thai adults. *Malaysian Journal of Nutrition, 24*(3).
227	L, H. S., H, K. W., & Munira, ida J. (2011). Influence of Food Intake and Eating Habits on Hypertension Control among Outpatients Influence of Food Intake and Eating Habits on Hypertension Control among Outpatients at a Government Health Clinic in the Klang Valley, Malaysia. *Malaysian Journal of Nutrition, 17*(2).
228	Lachat, C. K., Verstraeten, R., Khanh, L. N. B., Hagströmer, M., Khan, N. C., Van, N. D. A., Dung, N. Q., & Kolsteren, P. W. (2008). Validity of two physical activity questionnaires (IPAQ and PAQA) for Vietnamese adolescents in rural and urban areas. *International Journal of Behavioral Nutrition and Physical Activity*, *5*. https://doi.org/10.1186/1479-5868-5-37
229	Law, L. S., Sulaiman, N., Gan, W. Y., Adznam, S. N., & Taib, M. N. M. (2020). Predictors of overweight and obesity and its consequences among senoi orang asli (Indigenous people) women in Perak, Malaysia. *International Journal of Environmental Research and Public Health*, *17*(7). https://doi.org/10.3390/ijerph17072354
230	Lee, A. M. H., Ng, C. G., Koh, O. H., Gill, J. S., & Aziz, S. A. (2018). Metabolic syndrome in first episode schizophrenia, based on the national mental health registry of schizophrenia (NMHR) in a general hospital in Malaysia: A 10-year retrospective cohort study. *International Journal of Environmental Research and Public Health*, *15*(5). https://doi.org/10.3390/ijerph15050933
231	Lee, C. Y., Yusof, H. M., & Zakaria, N. S. (2019). Knowledge, attitude and behaviours related to weight control and body-image perceptions among Chinese high school students. *Malaysian Journal of Medical Sciences*, *26*(5), 122–131. https://doi.org/10.21315/mjms2019.26.5.11
232	Lee, K. S., Loprinzi, P. D., & Trost, S. G. (2010). Determinants of physical activity in Singaporean adolescents. *International Journal of Behavioral Medicine*, *17*(4), 279–286. https://doi.org/10.1007/s12529-009-9060-6
233	Lee, S. C., Hairi, N. N., & Moy, F. M. (2017). Metabolic syndrome among non-obese adults in the teaching profession in Melaka, Malaysia. *Journal of Epidemiology*, *27*(3), 130–134. https://doi.org/10.1016/j.je.2016.10.006
234	Lee, S. T., Wong, J. E., Ong, W. W., Ismail, M. N., Deurenberg, P., & Poh, B. K. (2016). Physical Activity Pattern of Malaysian Preschoolers: Environment, Barriers, and Motivators for Active Play. *Asia-Pacific Journal of Public Health*, *28*, 21S-34S. https://doi.org/10.1177/1010539516638155
235	Lee, S. T., Wong, J. E., Shanita, S. N., Ismail, M. N., Deurenberg, P., & Poh, B. K. (2015). Daily physical activity and screen time, but not other sedentary activities, are associated with measures of obesity during childhood. *International Journal of Environmental Research and Public Health*, *12*(1), 146–161. https://doi.org/10.3390/ijerph120100146
236	Lee, T., Khor, W., Tan, N., Cheng, C., Seow, A., & Foo, S. C. (1999). A cross-sectional survey of physical activity among middle aged women in Singapore. *Singapore Medical Journal*, *40*(7), 468–476. https://www.researchgate.net/publication/12741440
237	Lee, Y. S., Kek, B. L. K., Poh, L. K. S., Saw, S. M., & Loke, K. Y. (2008). Association of raised liver transaminases with physical inactivity, increased waist hip ratio, and other metabolic morbidities in severely obese children. *Journal of Pediatric Gastroenterology and Nutrition*, *47*(2), 172–178. https://doi.org/10.1097/MPG.0b013e318162a0e5
238	Lee, Y. Y., & Manan, W. (2015). Anthropometric Characteristics and Physical Activity of College-Age Adults in Selected Institutions of Higher Learning in Kelantan. *Malaysian Journal of Nutrition*. https://www.researchgate.net/publication/278684724
239	Lee, Y. Y., & Manan, W. (2017). Accelerometer-Measured Physical Activity and its Relationship with Body Mass Index (BMI) and Waist Circumference (WC) Measurements: A Cross-Sectional Study on Malaysian Adults. *Malaysian Journal of Nutrition*, *23*(3), 397–408. https://www.researchgate.net/publication/321715149
240	Lee, Y. Y., Kamarudin, K. S., & Wan Muda, W. A. M. (2019). Associations between self-reported and objectively measured physical activity and overweight/obesity among adults in Kota Bharu and Penang, Malaysia. *BMC Public Health*, *19*(1). https://doi.org/10.1186/s12889-019-6971-2
241	Lee, Y. Y., Khoo, S., Morris, T., Hanlon, C., Wee, L. H., Teo, E. W., & Adnan, Y. (2016). A mixed-method study of the efficacy of physical activity consultation as an adjunct to standard smoking cessation treatment among male smokers in Malaysia. *SpringerPlus*, *5*(1). https://doi.org/10.1186/s40064-016-3675-2
242	Lely Suratri, M. A., N, I. T., & Setiawaty, V. (2018). Correlation between dental health maintenance behavior with Dental Caries Status (DMF-T). *Bali Medical Journal*, *7*(1), 56. https://doi.org/10.15562/bmj.v7i1.836
243	Lerssrimongkol, C., Wisetborisut, A., Angkurawaranon, C., Jiraporncharoen, W., & Lam, K. B. H. (2016). Active commuting and cardiovascular risk among health care workers. *Occupational Medicine*, *66*(6), 483–487. https://doi.org/10.1093/occmed/kqw029
244	Leung, Y. Y., Ang, L. W., Thumboo, J., Wang, R., Yuan, J. M., & Koh, W. P. (2014). Cigarette smoking and risk of total knee replacement for severe osteoarthritis among Chinese in Singapore—the Singapore Chinese health study. *Osteoarthritis and Cartilage*, *22*(6), 764–770. https://doi.org/10.1016/j.joca.2014.03.013
245	Leung, Y. Y., Razak, H. R. B. A., Talaei, M., Ang, L. W., Yuan, J. M., & Koh, W. P. (2018). Duration of physical activity, sitting, sleep and the risk of total knee replacement among Chinese in Singapore, the Singapore Chinese health study. *PLoS ONE*, *13*(9). https://doi.org/10.1371/journal.pone.0202554
246	Liau, A. K., Neihart, M., Teo, C. T., Goh, L. S., & Chew, P. (2018). A Quasi-Experimental Study of a Fitbit-Based Self-Regulation Intervention to Improve Physical Activity, Well-Being, and Mental Health. *Cyberpsychology, Behavior, and Social Networking*, *21*(11), 727–734. https://doi.org/10.1089/cyber.2016.0502
247	Liau, S. Y., Shafie, A. A., Ibrahim, M. I. M., Hassali, M. A., Othman, A. T., Mohamed, M. H. N., & Hamdi, M. A. (2013). Stages of change and health-related quality of life among employees of an institution. *Health Expectations*, *16*(2), 199–210. https://doi.org/10.1111/j.1369-7625.2011.00702.x
248	Liew, C. F., Seah, E. S., Yeo, K. P., Lee, K. O., & Wise, S. D. (2003). Lean, nondiabetic Asian Indians have decreased insulin sensitivity and insulin clearance, and raised leptin compared to Caucasians and Chinese subjects. *International Journal of Obesity*, *27*(7), 784–789. https://doi.org/10.1038/sj.ijo.0802307
249	Liew, S. F., Chuah, H. S., Lau, C. L., Lee, C. H., & Say, Y. H. (2009). Prevalence of the leptin and leptin receptor gene variants and obesity risk factors among Malaysian university students of Setapak, Kuala Lumpur. *Asian Journal of Epidemiology*, *2*(3), 49–58. https://doi.org/10.3923/aje.2009.49.58
250	Lim, R. B. T., Wee, W. K., For, W. C., Ananthanarayanan, J. A., Soh, Y. H., Goh, L. M. L., Tham, D. K. T., & Wong, M. L. (2020). Correlates, facilitators and barriers of physical activity among primary care patients with prediabetes in Singapore—A mixed methods approach. *BMC Public Health*, *20*(1). https://doi.org/10.1186/s12889-019-7969-5
251	Lim, S.-C., Tan, B.-Y., Chew, S.-K., & Tan, C.-E. (2002). The relationship between insulin resistance and cardiovascular risk factors in overweight=obese non-diabetic Asian adults: the 1992 Singapore National Health Survey. *International Journal of Obesity*, *26*, 1511–1516. https://doi.org/10.1038=sj.ijo.0802140
252	Lim, T. O., Rugayah, B., & Maimunah, A. H. (2004). Familial Aggregation and Determinants of Post Challenge Blood Glucose in Four Ethnic Populations. *The Medical Journal of Malaysia*, *59*(3), 357–371.
253	Lin Khor, G., & Mohd Sharif, Z. (2003). Dual forms of malnutrition in the same households in Malaysia-a case study among Malay rural households. *Asia Pacific Journal of Clinical Nutrition, 12*(4).
254	Ling, O., Leh, H., Shaharom, N. H., Marzukhi, M. A., Zannierah, S., & Marzuki, S. (2018). Healthy Lifestyle Of Urban Residents. Case Study: Sri Pahang Public Housing, Bangsar, Kuala Lumpur, Malaysia. *Journal of the Malaysian Institute of Planners*, *16*(3), 1–12.
255	Liu, Y., Shu, X. O., Wen, W., Saito, E., Shafiur Rahman, M., Tsugane, S., Tamakoshi, A., Xiang, Y. B., Yuan, J. M., Gao, Y. T., Tsuji, I., Kanemura, S., Nagata, C., Shin, M. H., Pan, W. H., Koh, W. P., Sawada, N., Cai, H., Li, H. L., … Zheng, W. (2018). Association of leisure-time physical activity with total and cause-specific mortality: A pooled analysis of nearly a half million adults in the Asia Cohort Consortium. *International Journal of Epidemiology*, *47*(3), 771–779. https://doi.org/10.1093/ije/dyy024
256	Liyanage, E., Krasilshchikov, O., Hashim, H. A., & Jawis, N. M. (2020). Prevalence of hamstring tightness and hamstring flexibility of 9-11 years old children of different obesity and physical activity levels in Malaysia and Sri Lanka. *Journal of Physical Education and Sport*, *20*, 338–343. https://doi.org/10.7752/jpes.2020.s1047
257	Loh, D. A., Moy, F. M., Zaharan, N. L., Jalaludin, M. Y., & Mohamed, Z. (2017). Sugar-sweetened beverage intake and its associations with cardiometabolic risks among adolescents. *Pediatric Obesity*, *12*(1), e1–e5. https://doi.org/10.1111/ijpo.12108
258	Loh, K. W., Rani, F., Chan, T. C., Loh, H. Y., Ng, C. W., & Moy, F. M. (2013). The Association Between Risk Factors and Hypertension in Perak, Malaysia. *The Medical Journal of Malaysia*, *68*(4), 291–296.
259	Loke, S. C., Lim, W. S., Someya, Y., Hamid, T. A., & Nudin, S. S. H. (2016). Examining the Disability Model from the International Classification of Functioning, Disability, and Health Using a Large Data Set of Community-Dwelling Malaysian Older Adults. *Journal of Aging and Health*, *28*(4), 704–725. https://doi.org/10.1177/0898264315609907
260	Long, N. H., Thanasilp, S., & Thato, R. (2016). A causal model for fatigue in lung cancer patients receiving chemotherapy. *European Journal of Oncology Nursing*, *21*, 242–247. https://doi.org/10.1016/j.ejon.2015.10.010
261	Low, J. M., Tan, M. Y., See, K. C., & Aw, M. M. (2018). Sleep, activity and fatigue reported by postgraduate year 1 residents: A prospective cohort study comparing the effects of night float versus the traditional overnight on-call system. *Singapore Medical Journal*, *59*(12), 652–655. https://doi.org/10.11622/smedj.2018036
262	Low, S., & Balaraman, T. (2017). Physical activity level and fall risk among community-dwelling older adults. *Journal of Physical Therapy Science*, *29*(7), 1121–1124. http://www.who.int/gho/ncd/risk_factors/physical_activity_text/en/
263	Lua, P. L., & Razif, S. M. (2012). Nutritional status and health-related quality of life of breast cancer patients on chemotherapy. *Malaysian Journal of Nutrition*, *18*(2), 173–184. https://www.researchgate.net/publication/261873782
264	Luglio, H. F., & Huriyati, E. (2018). The role of genetic variation in TCF7L2 and KCNJ11, dietary intake, and physical activity on fasting plasma glucagon-like peptide-1 in male adolescents. *Paediatrica Indonesiana*, *57*(5), 239. https://doi.org/10.14238/pi57.5.2017.239-45
265	Luglio, H. F., Sulistyoningrum, D. C., Huriyati, E., Lee, Y. Y., & Wan Muda, W. A. M. (2017). The gene-lifestyle interaction on leptin sensitivity and lipid metabolism in adults: A population based study. *Nutrients*, *9*(7). https://doi.org/10.3390/nu9070716
266	Maasakkers, C. M., Claassen, J. A. H. R., Gardiner, P. A., Olde Rikkert, M. G. M., Lipnicki, D. M., Scarmeas, N., Dardiotis, E., Yannakoulia, M., Anstey, K. J., Cherbuin, N., Haan, M. N., Kumagai, S., Narazaki, K., Chen, T., Ng, T. P., Gao, Q., Nyunt, M. S. Z., Crawford, J. D., Kochan, N. A., … Melis, R. J. F. (2020). The Association of Sedentary Behaviour and Cognitive Function in People Without Dementia: A Coordinated Analysis Across Five Cohort Studies from COSMIC. *Sports Medicine*, *50*(2), 403–413. https://doi.org/10.1007/s40279-019-01186-7
267	Maehara, M., Rah, J. H., Roshita, A., Suryantan, J., Rachmadewi, A., & Izwardy, D. (2019). Patterns and risk factors of double burden of malnutrition among adolescent girls and boys in Indonesia. *PLoS ONE*, *14*(8). https://doi.org/10.1371/journal.pone.0221273
268	Mahdi, B., Al-Zurfi, N., Aziz, A. A., Abdullah, M. R., Noor, N. M., Norhayati, D., & Noor, M. (2012). Waist Height Ratio Compared to Body Mass Index and Waist Circumference in Relation to Glycemic Control in Malay Type 2 Diabetes Mellitus Patients, Hospital Universiti Sains Malaysia. *International Journal of Collaborative Research on Internal Medicine & Public Health, 4*(4).
269	Mahmudiono, T., Al Mamun, A., Nindya, T. S., Andrias, D. R., Megatsari, H., & Rosenkranz, R. R. (2018). The effectiveness of nutrition education for overweight/obese mother with stunted children (NEO-MOM) in reducing the double burden of malnutrition. *Nutrients*, *10*(12). https://doi.org/10.3390/nu10121910
270	Majid, H. A., Amiri, M., Mohd Azmi, N., Su, T. T., Jalaludin, M. Y., & Al-Sadat, N. (2016). Physical activity, body composition and lipids changes in adolescents: Analysis from the MyHeART Study. *Scientific Reports*, *6*. https://doi.org/10.1038/srep30544
271	Manan, W. W., Whye Lian, C., Ching Thon, C., Hazmi, H., Sains Malaysia, U., & Kerian, K. (2019). An intervention based on the stages of change, health profiles and physical activity levels of overweight and obese adults in Sarawak, Malaysia-a feasibility study Wan Manan Wan Muda. *Malaysian Family Physician, 14*(3).
272	Mardiah, A. B., Nasir, M. M., & Jan, H. J. (2012). Accelerometer-Determined Physical Activity Level among Government Employees in Penang, Malaysia. *Malaysian Journal of Nutrition, 18*(1).
273	Marques, A., Henriques-Neto, D., Peralta, M., Martins, J., Demetriou, Y., Schönbach, D. M. I., & de Matos, M. G. (2020). Prevalence of physical activity among adolescents from 105 low, middle, and high-income countries. *International Journal of Environmental Research and Public Health*, *17*(9). https://doi.org/10.3390/ijerph17093145
274	Masood, M., & Reidpath, D. D. (2016). Intraclass correlation and design effect in BMI, physical activity and diet: a cross-sectional study of 56 countries. *BMJ Open*, *6*(1). https://doi.org/10.1136/bmjopen-2015
275	Mat Rosly, M., Halaki, M., Hasnan, N., Mat Rosly, H., Davis, G. M., & Husain, R. (2018). Leisure time physical activity participation in individuals with spinal cord injury in Malaysia: barriers to exercise. *Spinal Cord*, *56*(8), 806–818. https://doi.org/10.1038/s41393-018-0068-0
276	Mat Rosly, M., Halaki, M., Mat Rosly, H., Davis, G. M., Hasnan, N., & Husain, R. (2020). Malaysian adaptation of the physical activity scale for individuals with physical disabilities in individuals with spinal cord injury. *Disability and Rehabilitation*, *42*(14), 2067–2075. https://doi.org/10.1080/09638288.2018.1544294
277	Mathialagan, A., Nallasamy, N., & Razali, S. N. (2018). Physical activity and media environment as antecedents of childhood obesity in malaysia. *Asian Journal of Pharmaceutical and Clinical Research*, *11*(9), 287–292. https://doi.org/10.22159/ajpcr.2018.v11i9.17095
278	Mee Lian, W., Lee Gan, G., Hwee Pin, C., Wee, S., Ching Ye, H., & Lian, M. (1999). Correlates of Leisure-Time Physical Activity in an Elderly Population in Singapore. *American Journal of Public Health*, *89*(10), 1578–1580.
279	Mena, M. M., & Lee, N. H. (2013). Stool Patterns of Malaysian Adults with Functional Constipation: Association with Diet and Physical Activity. *Malaysian Journal of Nutrition, 19*(1).
280	Mitra, M., & Wulandari, W. (2019). Factors affecting uncontrolled blood pressure among elderly hypertensive patients in Pekanbaru City, Indonesia. *Open Access Macedonian Journal of Medical Sciences*, *7*(7), 1209–1213. https://doi.org/10.3889/oamjms.2019.255
281	Mohammadi, S., Sulaiman, S., Koon, P. B., Amani, R., & Hosseini, S. M. (2013). Impact of healthy eating practices and physical activity on quality of life among breast cancer survivors. *Asian Pacific Journal of Cancer Prevention*, *14*(1), 481–487. https://doi.org/10.7314/APJCP.2013.14.1.481
282	Mokhtari, T., Jamaluddin, R., & Saad, H. A. (2015). Lifestyle and psychological factors associated with body weight status among university students in Malaysia. *Pakistan Journal of Nutrition*, *14*(1), 18–28. https://doi.org/10.3923/pjn.2015.18.28
283	Montakarn, C., & Nuttika, N. (2016). Physical activity levels and prevalence of low back pain in Thai call-center operators. *Indian Journal of Occupational and Environmental Medicine*, *20*(3), 125–128. www.ijoem.com/printarticle.asp?issn=0973-2284;year=2016;volume=20;issue=3;spage=125;epage=128;aulast=Montakarn
284	Morinaka, T., Limtrakul, P. ngarm, Makonkawkeyoon, L., & Sone, Y. (2012). Comparison of variations between percentage of body fat, body mass index and daily physical activity among young Japanese and Thai female students. *Journal of Physiological Anthropology*, *31*, 21. https://doi.org/10.1186/1880-6805-31-21
285	Moy, F. M., & Loh, D. A. (2015). Cardiometabolic risks profile of normal weight obese and multi-ethnic women in a developing country. *Maturitas*, *81*(3), 389–393. https://doi.org/10.1016/j.maturitas.2015.04.011
286	Mph, K. L., Sota Phd, C., & Laopaiboon Phd, M. (2008). Factors Affecting Physical Activity of Rural Thai Midlife Women. *Journal of the Medical Association of Thailand, 91*(8). http://www.medassocthai.org/journal
287	Muda, S. (2006). The effectiveness of physical activity counseling at primary care clinic Hospital Universiti Sains Malaysia.
288	Mueller, N. T., Mueller, N. J., Odegaard, A. O., Gross, M. D., Koh, W. P., Yuan, J. M., & Pereira, M. A. (2013). Higher parity is associated with an increased risk of type-II diabetes in Chinese women: The Singapore Chinese Health Study. *BJOG: An International Journal of Obstetrics and Gynaecology*, *120*(12), 1483–1489. https://doi.org/10.1111/1471-0528.12364
289	Muhammad, H. F. L., Latifah, F. N., & Susilowati, R. (2018). The yo-yo effect of Ramadan fasting on overweight/obese individuals in Indonesian: A prospective study. *Mediterranean Journal of Nutrition and Metabolism*, *11*(2), 127–133. https://doi.org/10.3233/MNM-17188
290	Muhsin, A., & Ibrahim, S. S. (2018). Physical activity, screen time, serum lipid and body mass index among malay female university students. *International Journal of Research in Pharmaceutical Sciences*, *9*(Special Issue 2), 78–82. https://doi.org/10.26452/ijrps.v9iSPL2.1745
291	Müller, A. M., Khoo, S., & Morris, T. (2016). Text Messaging for Exercise Promotion in Older Adults From an Upper-Middle-Income Country: Randomized Controlled Trial. *Journal of Medical Internet Research*, *18*(1), e5. https://doi.org/10.2196/jmir.5235
292	Müller, A. M., Tan, C. S., Chu, A. H. Y., van Dam, R. M., & Müller-Riemenschneider, F. (2019). Associations between psychological factors and accelerometer-measured physical activity in urban Asian adults. *International Journal of Public Health*, *64*(5), 659–668. https://doi.org/10.1007/s00038-019-01203-6
293	Müller-Riemenschneider, F., Ng, S. H. X., Koh, D., & Chu, A. H. Y. (2016). Objectively measured patterns of activities of different intensity categories and steps taken among working adults in a multi-ethnic asian population. *Journal of Occupational and Environmental Medicine*, *58*(6), e206–e211. https://doi.org/10.1097/JOM.0000000000000745
294	Munir, A. C., & Singh, R. (2016). Assessing Physical Activity Levels of Elderly Malays Living in Semi-Rural Areas Using Tri-Axial Accelerometer. *Malaysian Journal of Nutrition*, *22*(3), 363–374.
295	Murang, Z. R., Tuah, N. A. A., & Naing, L. (2020). Knowledge, attitude and practice towards eating and physical activity among primary school children in Brunei: A cross-sectional study. *International Journal of Adolescent Medicine and Health*, *32*(2). https://doi.org/10.1515/ijamh-2017-0118
296	Murayama, N., & Ohtsuka, R. (1999). Heart Rate Indicators for Assessing Physical Activity Level in the Field. *American Journal of Human Biology, 11*(5), 647-657.
297	Musa, N. P., Frederick, S., & Chong, V. H. (2013). Correlation of bowel habits with physical activity and fibre intake. *Brunei International Medical Journal*, *9*(6), 358–365. http://www.ipaq.ki.se/ipaq.htm
298	Naing, C., Lai, P. K., & Mak, J. W. (2017). Immediately modifiable risk factors attributable to colorectal cancer in Malaysia. *BMC Public Health*, *17*(1). https://doi.org/10.1186/s12889-017-4650-8
299	Nang, E. E. K., Gitau Ngunjiri, S. A., Wu, Y., Salim, A., Tai, E. S., Lee, J., & Van Dam, R. M. (2011). Validity of the international physical activity questionnaire and the Singapore prospective study program physical activity questionnaire in a multiethnic urban Asian population. *BMC Medical Research Methodology*, *11*. https://doi.org/10.1186/1471-2288-11-141
300	Nang, E. E. K., Salim, A., Wu, Y., Tai, E. S., Lee, J., & Van Dam, R. M. (2013). Television screen time, but not computer use and reading time, is associated with cardio-metabolic biomarkers in a multiethnic Asian population: A cross-sectional study. *International Journal of Behavioral Nutrition and Physical Activity*, *10*. https://doi.org/10.1186/1479-5868-10-70
301	Nang, E. E. K., Van Dam, R. M., Tan, C. S., Mueller-Riemenschneider, F., Lim, Y. T., Ong, K. Z., Ee, S., Lee, J., & Tai, E. S. (2015). Association of television viewing time with body composition and calcified subclinical atherosclerosis in Singapore Chinese. *PLoS ONE*, *10*(7). https://doi.org/10.1371/journal.pone.0132161
302	Nawi, A. M., Ilyani, F., & Jamaludin, C. (2015). Effect of Internet-based Intervention on Obesity among Adolescents in Kuala Lumpur: A School-based Cluster Randomised Trial. *Malaysian Journal of Medical Sciences*, *22*(4), 47–56. www.mjms.usm.my
303	Nelson, K., Lohsoonthorn, V., & Williams, M. A. (2009). Preterm Delivery Risk in Relation to Maternal Occupational and Leisure Time Physical Activity Among Thai Women. *Asian Biomedicine: Research, Reviews and News*, *3*(3), 267–277.
304	Neo, J. E., Salleh, S. B. M., Toh, Y. X., How, K. Y. L., Tee, M., Mann, K., Hopkins, S., Thielecke, F., Seal, C. J., & Brownlee, I. A. (2016). Whole-grain food consumption in Singaporean children aged 6–12 years. *Journal of Nutritional Science*, *5*. https://doi.org/10.1017/jns.2016.25
305	Ng, A. K., Hairi, N. N., Jalaludin, M. Y., & Majid, H. A. (2019). Dietary intake, physical activity and muscle strength among adolescents: The Malaysian Health and Adolescents Longitudinal Research Team (MyHeART) study. *BMJ Open*, *9*(6). https://doi.org/10.1136/bmjopen-2018-026275
306	Ng, L. P., Koh, Y. L. E., & Tan, N. C. (2020). Physical activity and sedentary behaviour of ambulatory older adults in a developed Asian community: A cross-sectional study. *American Journal of Physiology*—*Regulatory Integrative and Comparative Physiology*, *133*(1515), 266–271. https://doi.org/10.11622/smedj.2020022
307	Ng, T. P., Feng, L., Nyunt, M. S. Z., Feng, L., Niti, M., Tan, B. Y., Chan, G., Khoo, S. A., Chan, S. M., Yap, P., & Yap, K. B. (2015). Nutritional, Physical, Cognitive, and Combination Interventions and Frailty Reversal among Older Adults: A Randomized Controlled Trial. *American Journal of Medicine*, *128*(11), 1225-1236.e1. https://doi.org/10.1016/j.amjmed.2015.06.017
308	Ng, T. P., Nyunt, M. S. Z., Shuvo, F. K., Eng, J. Y., Yap, K. B., Hee, L. M., Chan, S. P., & Scherer, S. (2018). The Neighborhood Built Environment and Cognitive Function of Older Persons: Results from the Singapore Longitudinal Ageing Study. *Gerontology*, *64*(2), 149–156. https://doi.org/10.1159/000480080
309	Ngan, H. T. D., Tuyen, L. D., Van Phu, P., & Nambiar, S. (2018). Childhood overweight and obesity amongst primary school children in Hai Phong City, Vietnam. *Asia Pacific Journal of Clinical Nutrition*, *27*(2), 399–405. https://doi.org/10.6133/apjcn.062017.08
310	Ngo, C. S., Pan, C. W., Finkelstein, E. A., Lee, C. F., Wong, I. B., Ong, J., Ang, M., Wong, T. Y., & Saw, S. M. (2014). A cluster randomised controlled trial evaluating an incentive-based outdoor physical activity programme to increase outdoor time and prevent myopia in children. *Ophthalmic and Physiological Optics*, *34*(3), 362–368. https://doi.org/10.1111/opo.12112
311	Nguyen, C. L., Pham, N. M., Lee, A. H., Nguyen, P. T. H., Chu, T. K., Ha, A. V. V., Duong, D. V., Duong, T. H., & Binns, C. W. (2018). Physical activity during pregnancy is associated with a lower prevalence of gestational diabetes mellitus in Vietnam. *Acta Diabetologica*, *55*(9), 955–962. https://doi.org/10.1007/s00592-018-1174-3
312	Nguyen, H. L., Ha, D. A., Goldberg, R. J., Kiefe, C. I., Chiriboga, G., Ly, H. N., Nguyen, C. K., Phan, N. T., Vu, N. C., Nguyen, Q. P., & Allison, J. J. (2018). Culturally adaptive storytelling intervention versus didactic intervention to improve hypertension control in Vietnam- 12 month follow up results: A cluster randomized controlled feasibility trial. *PLoS ONE*, *13*(12). https://doi.org/10.1371/journal.pone.0209912
313	Nguyen, Q. N., Pham, S. T., Nguyen, V. L., Weinehall, L., Wall, S., Bonita, R., & Byass, P. (2012). Effectiveness of community-based comprehensive healthy lifestyle promotion on cardiovascular disease risk factors in a rural Vietnamese population: A quasi-experimental study. *BMC Cardiovascular Disorders*, *12*. https://doi.org/10.1186/1471-2261-12-56
314	Nguyen, T. H., Tang, H. K., Kelly, P., Van Der Ploeg, H. P., & Dibley, M. J. (2010). Association between physical activity and metabolic syndrome: A cross sectional survey in adolescents in Ho Chi Minh City, Vietnam. *BMC Public Health*, *10*. https://doi.org/10.1186/1471-2458-10-141
315	Nguyen, T. T., & Hoang, M. V. (2018). Non-communicable diseases, food and nutrition in Vietnam from 1975 to 2015: The burden and national response. *Asia Pacific Journal of Clinical Nutrition, 27*(1), 19-28. https://doi.org/10.6133/apjcn.032017.13
316	Nhung, B. T., Danh Tuyen, L., Linh, V. A., Do, N., Anh, V., Nga, T. T., Thi, V., Thuc, M., Yui, K., Ito, Y., Nakashima, Y., & Yamamoto, S. (2016). Rice Bran Extract Reduces the Risk of Atherosclerosis in Post-Menopausal Vietnamese Women. *Journal of Nutritional Science and Vitaminology, 62*.
317	Nicolosi, A., Moreira, E. D., Shirai, M., Ismail Bin Mohd Tambi, M., & Glasser, D. B. (2003). Epidemiology Of Erectile Dysfunction In Four Countries: Cross-National Study Of The Prevalence And Correlates Of Erectile Dysfunction. In *Urology, 61*.
318	Nik Yahya, N. S. R., Jamaludin, F. I. C., Firdaus, M. K. Z. H., & Che Hasan, M. K. (2019). A feasibility of simulation-based exercise programme for overweight adult in higher learning institutions. *Enfermeria Clinica*, *29*, 521–527. https://doi.org/10.1016/j.enfcli.2019.04.079
319	Noor Hassim, I., Norazman, M. R., Diana, M., Khairul Hazdi, Y., & Rosnah, I. (2016). Cardiovascular risk assessment between urban and rural population in Malaysia. *The Medical Journal of Malaysia*, *71*(6), 331–337.
320	Nor Aini, J., Poh, B. K., & Chee, W. S. S. (2013). Validity of a children’s physical activity questionnaire (cPAQ) for the study of bone health. *Pediatrics International*, *55*(2), 223–228. https://doi.org/10.1111/ped.12035
321	Nor, N. K., Ghozali, A. H., & Ismail, J. (2019). Prevalence of overweight and obesity among children and adolescents with autism spectrum disorder and associated risk factors. *Frontiers in Pediatrics*, *7*(FEB). https://doi.org/10.3389/fped.2019.00038
322	Notohartojo, I. T., Suratri, M. L., & Setiawaty, V. (2019). The association between hypertension, physical activity, and brushing technique with periodontal disease. *Bali Medical Journal*, *8*(1), 216. https://doi.org/10.15562/bmj.v8i1.1324
323	Nur Emilia, A., Yaaco, L. H., & Azidah, A. K. (2018). Pedometer-based walking intervention with and without group support among sedentary adults in primary care patients in north-east Malaysia: A randomized controlled trial. *Bangladesh Journal of Medical Science*, *17*(1), 52–57. https://doi.org/10.3329/bjms.v17i1.35280
324	Nur Kalmi, Z., Saad, H. A., Taib, M. N. M., Yassin, Z., & Tabata, I. (2012). Objective Assessment of Physical Activity in the Workplace Setting. *Pakistan Journal of Nutrition*, *11*(6). https://doi.org/10.3923/pjn.2012.523.528
325	Nur, Z., & Nasir, M. (2014). Comparison of Physical Activity Prevalence among International Physical Activity Questionnaire (IPAQ), Steps/Day, and Accelerometer in a Sample of Government Employees in Kangar, Perlis, Malaysia. *Pertanika Journal of Science & Technology*, *22*(2), 401–417.
326	Nurwanti, E., Uddin, M., Chang, J. S., Hadi, H., Syed-Abdul, S., Su, E. C. Y., Nursetyo, A. A., Masud, J. H. B., & Bai, C. H. (2018). Roles of sedentary behaviors and unhealthy foods in increasing the obesity risk in adult men and women: A cross-sectional national study. *Nutrients*, *10*(6). https://doi.org/10.3390/nu10060704
327	Nyunt, M. S. Z., Shuvo, F. K., Eng, J. Y., Yap, K. B., Scherer, S., Hee, L. M., Chan, S. P., & Ng, T. P. (2015). Objective and subjective measures of neighborhood environment (NE): Relationships with transportation physical activity among older persons. *International Journal of Behavioral Nutrition and Physical Activity*, *12*(1). https://doi.org/10.1186/s12966-015-0276-3
328	Nyunt, M. S. Z., Soh, C. Y., Gao, Q., Gwee, X., Ling, A. S. L., Lim, W. S., Lee, T. S., Yap, P. L. K., Yap, K. B., & Ng, T. P. (2017). Characterisation of physical frailty and associated physical and functional impairments in mild cognitive impairment. *Frontiers in Medicine*, *4*(DEC). https://doi.org/10.3389/fmed.2017.00230
329	Oddo, V. M., Maehara, M., & Rah, J. H. (2019). Overweight in Indonesia: An observational study of trends and risk factors among adults and children. *BMJ Open*, *9*(9). https://doi.org/10.1136/bmjopen-2019-031198
330	Oddo, V. M., Maehara, M., Izwardy, D., Sugihantono, A., Ali, P. B., & Rah, J. H. (2019). Risk factors for nutrition-related chronic disease among adults in Indonesia. *PLoS ONE*, *14*(8). https://doi.org/10.1371/journal.pone.0221927
331	Odegaard, A. O., Koh, W. P., Gross, M. D., Yuan, J. M., & Pereira, M. A. (2011). Combined lifestyle factors and cardiovascular disease mortality in chinese men and women: The singapore chinese health study. *Circulation*, *124*(25), 2847–2854. https://doi.org/10.1161/CIRCULATIONAHA.111.048843
332	Omar, A., Husain, M. N., Jamil, A. T., Nor, N. S. M., Ambak, R., Fazliana, M., Zamri, N. L. A., & Aris, T. (2018). Effect of physical activity on fasting blood glucose and lipid profile among low income housewives in the MyBFF@home study. *BMC Women’s Health*, *18*. https://doi.org/10.1186/s12905-018-0598-9
333	Onunkwor, O. F., Al-Dubai, S. A. R., George, P. P., Arokiasamy, J., Yadav, H., Barua, A., & Shuaibu, H. O. (2016). A cross-sectional study on quality of life among the elderly in non-governmental organizations’ elderly homes in Kuala Lumpur. *Health and Quality of Life Outcomes*, *14*(1). https://doi.org/10.1186/s12955-016-0408-8
334	Ota, E., Haruna, M., Yanai, H., Suzuki, M., Duc Anh, D., Matsuzaki, M., Huu Tho, L., Ariyoshi, K., Yeo, S., & Murashima, S. (2008). Reliability and validity of the Vietnamese version of the Pregnancy Physical Activity Questionnaire (PPAQ). *Southeast Asian Journal of Tropical Medicine and Public Health*, *39*(3), 562–570.
335	Padmapriya, N., Bernard, J. Y., Liang, S., Loy, S. L., Cai, S., Zhe, I. S., Kwek, K., Godfrey, K. M., Gluckman, P. D., Saw, S. M., Chong, Y. S., Chan, J. K. Y., Müller-Riemenschneider, F., Sheppard, A., Chinnadurai, A., Neo Goh, A. E., Rifkin-Graboi, A., Qiu, A., Biswas, A., … Lee, Y. S. (2017). Associations of physical activity and sedentary behavior during pregnancy with gestational diabetes mellitus among Asian women in Singapore. *BMC Pregnancy and Childbirth*, *17*(1). https://doi.org/10.1186/s12884-017-1537-8
336	Padmapriya, N., Bernard, J. Y., Liang, S., Loy, S. L., Shen, Z., Kwek, K., Godfrey, K. M., Gluckman, P. D., Chong, Y. S., Saw, S. M., Meaney, M. J., Chen, H., Müller-Riemenschneider, F., Agarwal, P., Biswas, A., Bong, C. L., Broekman, B. F. P., Cai, S., Chan, J. K. Y., … Yeo, G. S. H. (2016). Association of physical activity and sedentary behavior with depression and anxiety symptoms during pregnancy in a multiethnic cohort of Asian women. *Archives of Women’s Mental Health*, *19*(6), 1119–1128. https://doi.org/10.1007/s00737-016-0664-y
337	Padmapriya, N., Shen, L., Soh, S. E., Shen, Z., Kwek, K., Godfrey, K. M., Gluckman, P. D., Chong, Y. S., Saw, S. M., & Müller-Riemenschneider, F. (2015). Physical Activity and Sedentary Behavior Patterns Before and During Pregnancy in a Multi-ethnic Sample of Asian Women in Singapore. *Maternal and Child Health Journal*, *19*(11), 2523–2535. https://doi.org/10.1007/s10995-015-1773-3
338	Page, R. M., & Suwanteerangkul, J. (2009). Self-rated health, psychosocial functioning, and health-related behavior among Thai adolescents. *Pediatrics International*, *51*(1), 120–125. https://doi.org/10.1111/j.1442-200X.2008.02660.x
339	Page, R. M., West, J. H., & Hall, P. C. (2011). Psychosocial distress and suicide ideation in Chinese and Philippine adolescents. *Asia-Pacific Journal of Public Health, 23*(5), 774-791. https://doi.org/10.1177/1010539509353113
340	Palermo, M., & Sandoval, M. A. (2016). Assessment of physical activity level among patients with type 2 diabetes mellitus at the UP—Philippine general hospital diabetes clinic. *Journal of the ASEAN Federation of Endocrine Societies*, *31*(2), 144–149. https://doi.org/10.15605/jafes.031.02.10
341	Patalen, C. F., Guinto, S. E., Atrero, C. T., Joy Ducay, A. D., Duante, C. A., Capanzana, M. V, & Journal, P. (2018). Characteristics and Risk Factors for High Fasting Blood Glucose among Managers and Government Officials in the Philippines. *Science, 147*(4).
342	Pell, C., Allotey, P., Evans, N., Hardon, A., Imelda, J. D., Soyiri, I., & Reidpath, D. D. (2016). Coming of age, becoming obese: A cross-sectional analysis of obesity among adolescents and young adults in Malaysia. *BMC Public Health*, *16*(1). https://doi.org/10.1186/s12889-016-3746-x
343	Peltzer, K., & Pengpid, S. (2016). Leisure time physical inactivity and sedentary behaviour and lifestyle correlates among students aged 13–15 in the association of southeast asian nations (ASEAN) member states, 2007–2013. *International Journal of Environmental Research and Public Health*, *13*(2). https://doi.org/10.3390/ijerph13020217
344	Peltzer, K., & Pengpid, S. (2017). Dental health status and oral health behavior among university students from five ASEAN countries. *Nagoya Journal of Medical Science*, *79*(2), 123–133. https://doi.org/10.18999/nagjms.79.2.123
345	Peltzer, K., & Pengpid, S. (2017). Dietary behaviors, psychologicalwell-being, and mental distress among university students in ASEAN. *Iranian Journal of Psychiatry and Behavioral Sciences*, *11*(2). https://doi.org/10.5812/ijpbs.10118
346	Peltzer, K., & Pengpid, S. (2017). The Association of Dietary Behaviors and Physical Activity Levels with General and Central Obesity among ASEAN University Students. *AIMS Public Health*, *4*(3), 301–303. https://doi.org/10.3934/publichealth.2017.3.301
347	Peltzer, K., & Pengpid, S. (2018). Prevalence, risk awareness and health beliefs of behavioural risk factors for cardiovascular disease among university students in nine ASEAN countries. *BMC Public Health*, *18*(1). https://doi.org/10.1186/s12889-018-5142-1
348	Peltzer, K., & Pengpid, S. (2018). The Prevalence and Social Determinants of Hypertension among Adults in Indonesia: A Cross-Sectional Population-Based National Survey. *International Journal of Hypertension*, *2018*. https://doi.org/10.1155/2018/5610725
349	Peltzer, K., & Pengpid, S. (2019). Prevalence, social and health correlates of insomnia among persons 15 years and older in Indonesia. *Psychology, Health and Medicine*, *24*(6), 757–768. https://doi.org/10.1080/13548506.2019.1566621
350	Penglee, N., Christiana, R. W., Battista, R. A., & Rosenberg, E. (2019). Smartphone use and physical activity among college students in health science-related majors in the United States and Thailand. *International Journal of Environmental Research and Public Health*, *16*(8). https://doi.org/10.3390/ijerph16081315
351	Pengpid, S., & Peltzer, K. (2015). Prevalence of overweight and underweight and its associated factors among male and female university students in Thailand. *HOMO- Journal of Comparative Human Biology*, *66*(2), 176–186. https://doi.org/10.1016/j.jchb.2014.11.002
352	Pengpid, S., & Peltzer, K. (2016). Parental Involvement, Health Behaviors and Mental Health among School-going Adolescents in Six Asian Countries. *ASR: Chiang Mai University Journal of Social Sciences and Humanities*, *3*(2). https://doi.org/10.12982/CMUJASR.2016.0007
353	Pengpid, S., & Peltzer, K. (2017). Multimorbidity in chronic conditions: Public primary care patients in four greater mekong countries. *International Journal of Environmental Research and Public Health*, *14*(9). https://doi.org/10.3390/ijerph14091019
354	Pengpid, S., & Peltzer, K. (2019). Behavioral risk factors of non-communicable diseases among a nationally representative sample of school-going adolescents in Indonesia. *International Journal of General Medicine*, *12*, 387–394. https://doi.org/10.2147/IJGM.S226633
355	Pengpid, S., & Peltzer, K. (2019). High sedentary behaviour and low physical activity are associated with anxiety and depression in Myanmar and Vietnam. *International Journal of Environmental Research and Public Health*, *16*(7). https://doi.org/10.3390/ijerph16071251
356	Pengpid, S., & Peltzer, K. (2020). Overweight or obesity and related lifestyle and psychosocial factors among adolescents in Brunei Darussalam. *International Journal of Adolescent Medicine and Health*, *32*(6). https://doi.org/10.1515/ijamh-2018-0019
357	Pengpid, S., Peltzer, K., Jayasvasti, I., Aekplakorn, W., Puckpinyo, A., Nanthananate, P., & Mansin, A. (2019). Two-year results of a community-based randomized controlled lifestyle intervention trial to control prehypertension and/or prediabetes in Thailand: A brief report. *International Journal of General Medicine*, *12*, 131–135. https://doi.org/10.2147/IJGM.S200086
358	Pengpid, S., Vonglokham, M., Kounnavong, S., Sychareun, V., & Peltzer, K. (2019). Concurrent binge drinking and current tobacco use and its social and health correlates among adults in Laos. *Journal of Human Behavior in the Social Environment*, *29*(3), 403–414. https://doi.org/10.1080/10911359.2018.1541037
359	Pengpid, S., Vonglokham, M., Kounnavong, S., Sychareun, V., & Peltzer, K. (2020). The prevalence of underweight and overweight/obesity and its correlates among adults in Laos: a cross-sectional national population-based survey, 2013. *Eating and Weight Disorders*, *25*(2), 265–273. https://doi.org/10.1007/s40519-018-0571-5
360	Pham, L. H., Au, T. B., Blizzard, L., Truong, N. B., Schmidt, M. D., Granger, R. H., & Dwyer, T. (2009). Prevalence of risk factors for non-communicable diseases in the Mekong Delta, Vietnam: Results from a steps survey. *BMC Public Health*, *9*. https://doi.org/10.1186/1471-2458-9-291
361	Pham, T. A. V., & Nguyen, P. A. (2019). Factors related to dental caries in 10-year-old Vietnamese schoolchildren. *International Dental Journal*, *69*(3), 214–222. https://doi.org/10.1111/idj.12452
362	Pham, T., Bui, L., Nguyen, A., Nguyen, B., Tran, P., Vu, P., & Dang, L. (2019). The prevalence of depression and associated risk factors among medical students: An untold story in Vietnam. *PLoS ONE*, *14*(8). https://doi.org/10.1371/journal.pone.0221432
363	Phing, C. H., Hoa, L. S., & Hua, T. X. (2017). Overweight and obesity in relation to cardiovascular risk factors among university students in Malaysia. In *Romanian Journal of Diabetes, Nutrition and Metabolic Diseases, 24*(3), 195-201. https://doi.org/10.1515/rjdnmd-2017-0025
364	Phosat, C., Panprathip, P., Chumpathat, N., Prangthip, P., Chantratita, N., Soonthornworasiri, N., Puduang, S., & Kwanbunjan, K. (2017). Elevated C-reactive protein, interleukin 6, tumor necrosis factor alpha and glycemic load associated with type 2 diabetes mellitus in rural Thais: A cross-sectional study. *BMC Endocrine Disorders*, *17*(1). https://doi.org/10.1186/s12902-017-0189-z
365	Pichayapinyo, P., Saslow, L. R., Aikens, J. E., Marinec, N., Sillabutra, J., Rattanapongsai, P., & Piette, J. D. (2019). Feasibility study of automated interactive voice response telephone calls with community health nurse follow-up to improve glycaemic control in patients with type 2 diabetes. *International Journal of Nursing Practice*, *25*(6). https://doi.org/10.1111/ijn.12781
366	Piya-Amornphan, N., Santiworakul, A., Cetthakrikul, S., & Srirug, P. (2020). Physical activity and creativity of children and youths. *BMC Pediatrics*, *20*(1). https://doi.org/10.1186/s12887-020-2017-2
367	Poh, B., Safiah, Y., Aris, T., Haslinda, S., Norimah, A., Wan Manan, W., Mirnalini, K., Zalilah, M., Azmi, M., & Fatimah, S. (2010). Physical Activity Pattern and Energy Expenditure of Malaysian Adults: Findings from the Malaysian Adult Nutrition Survey (MANS). *Malaysian Journal of Nutrition*, *16*(1), 13–37. https://www.researchgate.net/publication/225300363
368	Poh, H., Eastwood, P. R., Jenkins, S., Cecins, N. M., Ho, K. T., & Jenkins, S. C. (2006). Six-minute walk distance in healthy Singaporean adults cannot be predicted using reference equations derived from Caucasian populations. *Respirology, 11*.
369	Pon Msc, L. W., Noor-Aini Phd, M. Y., Frcog, N. A., Mog, S. S., Phd, S., Mohamed, A. L., Mrcpath, H., Mrcog, A. M., & Mog, W. (2006). Diet, nutritional knowledge and health status of urban middle-aged Malaysian women. *Asia Pacific Journal of Clinical Nutrition, 15*(3).
370	Pongchaiyakul, C., Nguyen, T. V., Kosulwat, V., Rojroongwasinkul, N., Charoenkiatkul, S., Eisman, J. A., & Rajatanavin, R. (2004). Effects of physical activity and dietary calcium intake on bone mineral density and osteoporosis risk in a rural Thai population. *Osteoporosis International*, *15*(10), 807–813. https://doi.org/10.1007/s00198-004-1613-6
371	Prachuntasen, K., Laohasiriwong, W., & Luenam, A. (2018). Social capital associated with quality of life among late adults and elderly population in the northeast of Thailand [version 1; peer review: 2 approved with reservations]. *F1000Research*, *7*. https://doi.org/10.12688/F1000RESEARCH.13954.1
372	Pradono, J., Kusumawardani, N., & Delima, D. (2015). Central obesity increases the risk of type 2 diabetes mellitus among urban adults. *Universa Medicina*, *34*(3). https://doi.org/10.18051/UnivMed.2016.v35.187-196
373	Promthet, S., Saranrittichai, K., Kamsa-ard, S., Senarak, W., Vatanasapt, P., Wiangnon, S., Wongphuthorn, P., & Moore, M. (2011). Situation Analysis of Risk Factors Related to Non-communicable Diseases in Khon Kaen Province, Thailand. *Asian Pacific Journal of Cancer Prevention*, *12*(5), 1337–1340.
374	Proverawati, A., & Wahyurin, I. S. (2019). Fruit and vegetables intakes, physical activity and nutritional status in adolescent: Study in Banyumas-Indonesia. *Annals of Tropical Medicine and Public Health*, *22*(11). https://doi.org/10.36295/ASRO.2019.22119
375	Purba, E. N., Santosa, H., & Siregar, F. A. (2019). The relationship of physical activity and obesity with the incidence of hypertension in adults aged 26-45 years in Medan. *Open Access Macedonian Journal of Medical Sciences*, *7*(20), 3464–3468. https://doi.org/10.3889/oamjms.2019.447
376	Quang Vo, T., Thanh Thi Nguyen, H., & Phuong Ngoc Ta, A. (2020). Effect of sociodemographic factors on quality of life of medical students in southern Vietnam: A survey using the WHOQOL-BREF assessment Quality of life of medical students in Vietnam. *Journal of Pharmacy & Pharmacognosy Research*, *8*(3), 211–224. http://jppres.com/jppreshttp://jppres.com/
377	Rachmah, Q., Setyaningtyas, S. W., Rifqi, M. A., Indriani, D., Nindya, T. S., Megatsari, H., Mahmudiono, T., & Kriengsinyos, W. (2019). Self-efficacy to engage in physical activity and overcome barriers, sedentary behavior, and their relation to body mass index among elderly Indonesians with diabetes. *Journal of Preventive Medicine and Public Health*, *52*(4), 242–249. https://doi.org/10.3961/jpmph.19.003
378	Rahim, F. F., Abdulrahman, S. A., Maideen, S. F. K., & Rashid, A. (2020). Prevalence and factors associated with prediabetes and diabetes in fishing communities in Penang, Malaysia: A cross-sectional study. *PLoS ONE*, *15*(2). https://doi.org/10.1371/journal.pone.0228570
379	Ramli, A., Henry, L. J., Fuan Liang, Y., & Beh, Y. (2013). Effects of a Worksite Health Programme on the Improvement of Physical Health among Overweight and Obese Civil Servants: A Pilot Study. *Malaysian Journal of Medical Sciences*, *20*(5), 54–60. www.mjms.usm.my
380	Ramli, A., Joseph, L., Vijaykumar, P., Suhaimy, R.M. (2013). Obesity and habitual physical activity level among staffs working in a Military Hospital in Malacca, Malaysia. *International Medical Journal Malaysia 12*(1), 53-58.
381	Rangsin, R. (2007). Prevalence of overweight and obesity in Royal Thai Army personnel. *Journal of the Medical Association of Thailand = Chotmaihet thangphaet*. https://www.researchgate.net/publication/6430943
382	Rasiah, R., Thangiah, G., Yusoff, K., Manikam, R., Chandrasekaran, S. K., Mustafa, R., & Bakar, N. B. A. (2015). The impact of physical activity on cumulative cardiovascular disease risk factors among Malaysian adults. *BMC Public Health*, *15*(1). https://doi.org/10.1186/s12889-015-2577-5
383	Ratna Noer, E., & Purnawian, R. (2018). Physical inactivity, high carbohydrate intake, and metabolic factors associated with abdominal obesity among Indonesian adolescents. *Pakistan Journal of Medical and Health Sciences, 12*(3). https://www.researchgate.net/publication/329865057
384	Rees, S., Silove, D., Chey, T., Steel, Z., Bauman, A., & Phan, T. (2012). Physical activity and psychological distress amongst Vietnamese living in the Mekong Delta. *Australian and New Zealand Journal of Psychiatry*, *46*(10), 966–971. https://doi.org/10.1177/0004867412459568
385	Rimpeekool, W., Yiengprugsawan, V., Kirk, M., Banwell, C., Seubsman, S. A., & Sleigh, A. (2017). Nutrition label experience, obesity, high blood pressure, and high blood lipids in a cohort of 42,750 Thai adults. *PLoS ONE*, *12*(12). https://doi.org/10.1371/journal.pone.0189574
386	Risonar, M. G. D., Rayco-Solon, P., Ribaya-Mercado, J. D., Solon, J. A. A., Cabalda, A. B., Tengco, L. W., & Solon, F. S. (2009). Physical activity, energy requirements, and adequacy of dietary intakes of older persons in a rural Filipino community. *Nutrition Journal*, *8*(1). https://doi.org/10.1186/1475-2891-8-19
387	Rizal, H., Hajar, M. S., Kueh, Y. C., Muhamad, A. S., & Kuan, G. (2019). Confirmatory factor analysis of the malay-language transtheoretical model of physical activity among malaysian primary school children. *Malaysian Journal of Medical Sciences*, *26*(2), 99–113. https://doi.org/10.21315/mjms2019.26.2.11
388	Rizal, H., Hajar, M. S., Muhamad, A. S., Kueh, Y. C., & Kuan, G. (2019). The effect of brain breaks on physical activity behaviour among primary school children: A transtheoretical perspective. *International Journal of Environmental Research and Public Health*, *16*(21). https://doi.org/10.3390/ijerph16214283
389	Rizidah, Z., Naing, L., Rizidah MURANG, Z., Tuah, N., & Anak Puteri Rashidah Sa, P. (2017). Parental perceptions of factors in-fluencing the development of child-hood obesity in Brunei Darussalam: A cross-sectional study. *Brunei International Medical Journal*. https://www.researchgate.net/publication/318108204
390	Rosli, H., Kee, Y., & Shahar, S. (2019). Dietary polyphenol intake associated with adiposity indices among adults from low to medium socioeconomic status in a suburban area of Kuala Lumpur: A preliminary findings. *Malaysian Journal of Medical Sciences*, *26*(6), 67–76. https://doi.org/10.21315/mjms2019.26.6.7
391	S, Z. M., L, K. G., K, N. A., Lecturer Khor L, S. G., Mirnalini, P. K., Professor Ang, A. M., Student, G., & Mohd Shariff, Z. (2006). Dietary intake, physical activity and energy expenditure of Malaysian adolescents. *Singapore Medical Journal, 47*(6).
392	Sagayadevan, V., Abdin, E., Binte Shafie, S., Jeyagurunathan, A., Sambasivam, R., Zhang, Y., Picco, L., Vaingankar, J., Chong, S. A., & Subramaniam, M. (2017). Prevalence and correlates of sleep problems among elderly Singaporeans. *Psychogeriatrics*, *17*(1), 43–51. https://doi.org/10.1111/psyg.12190
393	Samnieng, P., Ueno, M., Zaitsu, T., Shinada, K., Wright, F. A. C., & Kawaguchi, Y. (2013). The relationship between seven health practices and oral health status in community-dwelling elderly Thai. *Gerodontology*, *30*(4), 254–261. https://doi.org/10.1111/j.1741-2358.2012.00672.x
394	Sanee, A., Somrongthong, R., & Plianbangchang, S. (2017). The positive effects of a peer-led intervention system for individuals with a risk of metabolic syndrome. *Journal of Multidisciplinary Healthcare*, *10*, 293–300. https://doi.org/10.2147/JMDH.S142272
395	Sangeetha-Shyam, Fatimah, A. S., Rohana, A., Norasyikin, A., Karuthan, C., Shanita, S., Yusof, B., & Azmi, K. (2013). Lowering Dietary Glycaemic Index through Nutrition Education among Malaysian Women with a History of Gestational Diabetes Mellitus. *Malaysian Journal of Nutrition*, *19*(1), 9–23. https://www.researchgate.net/publication/237062295
396	Sangthong, R., Wichaidit, W., McNeil, E., Chongsuvivatwong, V., Chariyalertsak, S., Kessomboon, P., Taneepanichskul, S., Putwatana, P., & Aekplakorn, W. (2012). Health behaviors among short- and long- term ex-smokers: Results from the Thai National Health Examination Survey IV, 2009. *Preventive Medicine*, *55*(1), 56–60. https://doi.org/10.1016/j.ypmed.2012.04.022
397	Sankari, N., Halimatus, M., Vasudevan, R., & Mahalingam, K. (2020). University Putra Malaysia, Serdang, Selangor, Malaysia, *2 Faculty of Medicine and Health Sciences. *International Journal of Advanced Science and Technology*, *29*(8s), 971–980.
398	Santoso, N. K., Thongpat, S., & Wattanakul, B. (2015). Predictors Of Physical Activity In Older People With Hypertension, Bantul, Indonesia. *Journal of Health Research, 29*. https://doi.org/10.14456/jhr.2015.43
399	Saraswati, L. D., Udiyono, A., Sutrisni, D., & Fauzi, M. (2019). Sexual dysfunction among women with diabetes in a Primary Health Care at Semarang, Central Java Province, Indonesia. *Kesmas*, *14*(2), 95–102. https://doi.org/10.21109/kesmas.v14i2.2722
400	Sari, D. K., Tala, Z. Z., Lestari, S., Hutagalung, S. V., & Ganie, R. A. (2017). Vitamin D receptor gene polymorphism among Indonesian women in North Sumatera. *Asian Journal of Clinical Nutrition*, *9*(1), 44–50. https://doi.org/10.3923/ajcn.2017.44.50
401	Sari, D., Rasyid, H., Lipoeto, N., & Lubis, Z. (2014). Occurrence of Vitamin D Deficiency among Women in North Sumatera, Indonesia. *Malaysian Journal of Nutrition*, *20*(1), 63–70.
402	Sasongko, S. A., & Sevenhuysen, G. P. (1994). Body Mass and Physical Performance in Urban Indonesian Children 10-12 Years. *Medical Journal of Indonesia*, *3*(1), 4–18.
403	Say, Y. H., Ling, K. H., Duraisamy, G., Isaac, S., & Rosli, R. (2005). Angiotensinogen M235T gene variants and its association with essential hypertension and plasma renin activity in Malaysian subjects: A case control study. *BMC Cardiovascular Disorders*, *5*. https://doi.org/10.1186/1471-2261-5-7
404	Sazlina, S. G., Browning, C. J., & Yasin, S. (2015). Effectiveness of personalized feedback alone or combined with peer support to improve physical activity in sedentary older Malays with type 2 diabetes: A randomized controlled trial. *Frontiers in Public Health*, *3*(JUL). https://doi.org/10.3389/fpubh.2015.00178
405	Sazlina, S., Zaiton, A., Nor Afiah, M., & Hayati, K. (2012). Predictors of health related quality of life in older people with non-communicable diseases attending three primary care clinics in Malaysia. *The Journal of Nutrition, Health & Aging*, *16*(5), 498–502. https://doi.org/10.1007/s12603-012-0038-8
406	Schaafsma, A., Deurenberg, P., Calame, W., Van Den Heuvel, E. G. H. M., Van Beusekom, C., Hautvast, J., Sandjaja, S., Bee Koon, P., Rojroongwasinkul, N., Le Nguyen, B. K., Parikh, P., & Khouw, I. (2013). Design of the South East Asian Nutrition Survey (SEANUTS): A four-country multistage cluster design study. *British Journal of Nutrition*, *110*(SUPPL.3). https://doi.org/10.1017/S0007114513002067
407	Schmidt, G. J., Stensel, D. J., & Walkuski, J. J. (1997). Blood pressure, lipids, lipoproteins, body fat and physical activity of Singapore children. *Journal of Paediatrics and Child Health*, *33*, 484–490.
408	Schmidt, G. J., Walkuski, J. J., & Stensel, D. J. (1998). The Singapore youth coronary risk and physical activity study. *Medicine and Science in Sports and Exercise*, *30*(1), 105–113. https://doi.org/10.1097/00005768-199801000-00015
409	Seo, D. C., Torabi, M. R., Chin, M. K., Huang, S. F., Chen, C. K., Mok, M. M. C., Wong, P., Chia, M., Lee, C. G., & Wang, C. (2012). A comparison of factors associated with physical inactivity among East Asian college students. *International Journal of Behavioral Medicine*, *19*(3), 316–323. https://doi.org/10.1007/s12529-011-9167-4
410	Seo, D. C., Torabi, M. R., Chin, M. K., Lee, C. G., Kim, N., Huang, S. F., Chen, C. K., Mok, M. M. C., Wong, P., Chia, M., & Park, B. H. (2014). Physical activity, body mass index, alcohol consumption and cigarette smoking among East Asian college students. *Health Education Journal*, *73*(4), 453–465. https://doi.org/10.1177/0017896913485744
411	Seow, A., Quah, S. R., Nyam, D., Straughan, P. T., Chua, T., & Aw, T. C. (2002). Food groups and the risk of colorectal carcinoma in an Asian population. *Cancer*, *95*(11), 2390–2396. https://doi.org/10.1002/cncr.10971
412	Seow, D. Y. B., Haaland, B., & Jafar, T. H. (2015). The association of prehypertension with meals eaten away from home in young adults in Singapore. *American Journal of Hypertension*, *28*(10), 1197–1200. https://doi.org/10.1093/ajh/hpv027
413	Sh, L., Sh, F., & Yh, S. (2012). Plasma Total Antioxidant Capacity (TAC) in Obese Malaysian Subjects. *Malaysian Journal of Nutrition, 18*(3).
414	Shafinaz, I. S., & Moy, F. M. (2016). Vitamin D level and its association with adiposity among multi-ethnic adults in Kuala Lumpur, Malaysia: A cross sectional study. *BMC Public Health*, *16*(1). https://doi.org/10.1186/s12889-016-2924-1
415	Shahar, S., Mohd Salleh, R., Ghazali, A. R., Koon, P. B., & Wan Mohamud, W. N. (2010). Roles of Adiposity, Lifetime Physical Activity and Serum Adiponectin in Occurrence of Breast Cancer among Malaysian Women in Klang Valley. *Asian Pacific Journal of Cancer Prevention*, *11*(1), 61–66.
416	Shahar, S., Shafurah, S., Hasan Shaari, N., Rajikan, R., Rajab, N., Golkhalkhali, B., & Md Zainuddin, Z. (2011). Roles of Diet, Lifetime Physical Activity and Oxidative DNA Damage in the Occurrence of Prostate Cancer among Men in Klang Valley, Malaysia. *Asian Pacific Journal of Cancer Prevention*, *12*(3), 605–611.
417	Sharif, R., Chong, K. H., Zakaria, N. H., Ong, M. L., Reilly, J. J., Wong, J. E., Saad, H. A., & Poh, B. K. (2016). Results from Malaysia’s 2016 report card on physical activity for children and adolescents. *Journal of Physical Activity and Health*, *13*(11), S201–S205. https://doi.org/10.1123/jpah.2016-0404
418	Shariff, Z. M., & Khor, G. L. (2005). Obesity and household food insecurity: Evidence from a sample of rural households in Malaysia. *European Journal of Clinical Nutrition*, *59*(9), 1049–1058. https://doi.org/10.1038/sj.ejcn.1602210
419	Shazwani, N. M., Mastura, H. Y., & Fauzee, M. M. (2010). Assessment of Physical Activity Level among Individuals with Type 2 Diabetes Mellitus at Cheras Health Clinic, Kuala Lumpur. In *Malaysian Journal of Nutrition, 16*(1).
420	Shivappa, N., Wang, R., Hébert, J. R., Jin, A., Koh, W. P., & Yuan, J. M. (2019). Association between inflammatory potential of diet and risk of lung cancer among smokers in a prospective study in Singapore. *European Journal of Nutrition*, *58*(7), 2755–2766. https://doi.org/10.1007/s00394-018-1825-8
421	Shoko, M., Nakamori, M., Yagi, M., Saavedra, O. L., Ikemoto, S., & Yamamoto, S. (2009). Daily calcium intake and physical activity status in urban women living on low incomes in Davao, Philippines: a primary study for osteoporosis prevention. *The Journal of Medical Investigation*, *56*(3–4), 130–135. https://doi.org/10.2152/jmi.56.130.
422	Sirirassamee, T., Phoolsawat, S., & Limkhunthammo, S. (2018). Relationship between body weight perception and weight-related behaviours. *Journal of International Medical Research*, *46*(9), 3796–3808. https://doi.org/10.1177/0300060518780138
423	Sisson, S. B., & Katzmarzyk, P. T. (2008). International prevalence of physical activity in youth and adults. *Obesity Reviews, 9*(6), 606-614. https://doi.org/10.1111/j.1467-789X.2008.00506.x
424	Siti Affira, K., Mohd Nasir, M., Hazizi, A., & Kandiah, M. (2011). Socio-demographic and psychosocial factors associated with physical activity of working woman in Petaling Jaya, Malaysia. *Malaysian Journal of Nutrition*, *17*(3), 315–324. https://www.researchgate.net/publication/225096165
425	Sitthipornvorakul, E., Janwantanakul, P., & Van Der Beek, A. J. (2014). Correlation between pedometer and the Global Physical Activity Questionnaire on physical activity measurement in office workers. *BMC Research Notes*, *7*(1). https://doi.org/10.1186/1756-0500-7-280
426	Sloan, R. A., Kim, Y., Sawada, S. S., Asakawa, A., Blair, S. N., & Finkelstein, E. A. (2020). Is less sedentary behavior, more physical activity, or higher fitness associated with sleep quality? A cross-sectional study in Singapore. *International Journal of Environmental Research and Public Health*, *17*(4). https://doi.org/10.3390/ijerph17041337
427	Sloan, R. A., Sawada, S. S., Girdano, D., Liu, Y. T., Biddle, S. J., & Blair, S. N. (2013). Associations of sedentary behavior and physical activity with psychological distress: A cross-sectional study from Singapore. *BMC Public Health*, *13*(1), 1–8. https://doi.org/10.1186/1471-2458-13-885
428	Smagula, S. F., Koh, W. P., Wang, R., & Yuan, J. M. (2016). Chronic disease and lifestyle factors associated with change in sleep duration among older adults in the Singapore Chinese Health Study. *Journal of Sleep Research*, *25*(1), 57–61. https://doi.org/10.1111/jsr.12342
429	Sng, W. T., Yeo, S. N., Lin, B. X., & Lee, T. S. (2019). Impacts of apolipoprotein e disclosure on healthy Asian older adults: A cohort study. *International Psychogeriatrics*. https://doi.org/10.1017/S1041610218002089
430	Socorro Endrina-Ignacio, M. (2018). Behavioral Risk Factors for NCDs among School Children in the National Capital Region (NCR), Philippines. *Philippine Journal of Science, 147*(3).
431	Soh, W. H., Rajaram, N., Mariapun, S., Eriksson, M., Fadzli, F., Ho, W. K., Mohd Taib, N. A., Hall, P., & Teo, S. H. (2018). Physical activity and mammographic density in an Asian multi-ethnic cohort. *Cancer Causes and Control*, *29*(9), 883–894. https://doi.org/10.1007/s10552-018-1064-6
432	Song, L. L. T., Venkataraman, K., Gluckman, P., Chong, Y. S., Chee, M. W. L., Khoo, C. M., Leow, M. K. S., Lee, Y. S., Tai, E. S., & Khoo, E. Y. H. (2016). Smaller size of high metabolic rate organs explains lower resting energy expenditure in Asian-Indian Than Chinese men. *International Journal of Obesity*, *40*(4), 633–638. https://doi.org/10.1038/ijo.2015.233
433	Soo, K. L., Wan Abdul Manan, W. M., & Wan Suriati, W. N. (2015). The bahasa melayu version of the global physical activity questionnaire: Reliability and validity study in Malaysia. *Asia-Pacific Journal of Public Health*, *27*(2), NP184–NP193. https://doi.org/10.1177/1010539511433462
434	Sp, D., Nasir, M., & Zalilah, M. T. (2011). Factors Associated with Physical Activity Levels among Adolescents Attending School Determination of Factors Associated with Physical Activity Levels among Adolescents Attending School in Kuantan, Malaysia. *Malaysian Journal of Nutrition, 17*(2).
435	Sreeramareddy, C. T., Kutty, N. A. M., Jabbar, M. A. R., & Boo, N. Y. (2012). Physical activity and associated factors among young adults in Malaysia: An online exploratory survey. *BioScience Trends*, *6*(3), 103–109. https://doi.org/10.5582/bst.2012.v6.3.103
436	Srinonprasert, V., Chalermsri, C., Chailurkit, L. or, Ongphiphadhanakul, B., & Aekplakorn, W. (2018). Vitamin D insufficiency predicts mortality among older men, but not women: A nationwide retrospective cohort from Thailand. *Geriatrics and Gerontology International*, *18*(12), 1585–1590. https://doi.org/10.1111/ggi.13529
437	Sriramatr, S., Berry, T. R., & Spence, J. C. (2014). An internet-based intervention for promoting and maintaining physical activity: A randomized controlled trial. *American Journal of Health Behavior*, *38*(3), 430–439. https://doi.org/10.5993/AJHB.38.3.12
438	Sritara, C., Thakkinstian, A., Ongphiphadhanakul, B., Pornsuriyasak, P., Warodomwichit, D., Akrawichien, T., Vathesatogkit, P., & Sritara, P. (2015). Work- and travel-related physical activity and alcohol consumption: Relationship with bone mineral density and calcaneal quantitative ultrasonometry. *Journal of Clinical Densitometry*, *18*(1), 37–43. https://doi.org/10.1016/j.jocd.2014.04.117
439	Stubbs, B., Koyanagi, A., Schuch, F. B., Firth, J., Rosenbaum, S., Veronese, N., Solmi, M., Mugisha, J., & Vancampfort, D. (2016). Physical activity and depression: a large cross-sectional, population-based study across 36 low- and middle-income countries. *Acta Psychiatrica Scandinavica*, *134*(6), 546–556. https://doi.org/10.1111/acps.12654
440	Su, T. T., Azzani, M., Adewale, A. P., Thangiah, N., Zainol, R., & Majid, H. (2019). Physical activity and health-related quality of life among low-income adults in metropolitan kuala lumpur. *Journal of Epidemiology*, *29*(2), 43–49. https://doi.org/10.2188/jea.JE20170183
441	Su, T. T., Sim, P. Y., Nahar, A. M., Majid, H. A., Murray, L. J., Cantwell, M. M., Al-Sadat, N., & Jalaludin, M. Y. (2014). Association between self-reported physical activity and indicators of body composition in Malaysian adolescents. *Preventive Medicine*, *67*, 100–105. https://doi.org/10.1016/j.ypmed.2014.07.001
442	Subramaniam, M., Zhang, Y., Lau, J. H., Vaingankar, J. A., Abdin, E., Chong, S. A., & Lee, E. S. (2019). Patterns of physical activity and health-related quality of life amongst patients with multimorbidity in a multi-ethnic Asian population. *BMC Public Health*, *19*(1). https://doi.org/10.1186/s12889-019-7941-4
443	Suebsamran, P., Pimpak, T., Thani, P., & Chamnan, P. (2018). The Metabolic Syndrome and Health Behaviors in School Children Aged 13-16 Years in Ubon Ratchathani: UMeSIA Project. *Metabolic Syndrome and Related Disorders*, *16*(8), 425–432. https://doi.org/10.1089/met.2017.0150
444	Suhadi, R., Linawati, Y., Virginia, D. M., & Setiawan, C. H. (2015). Early Implementation of Universal Health Coverage Among Hypertension Subjects in Sleman District of Yogyakarta. *Acta Medica Indonesiana*, *47*(4), 311–319.
445	Suhaimy, R.M. (2013). Obesity and habitual physical activity level among staffs working in a Military Hospital in Malacca, Malaysia. *International Medical Journal Malaysia 12*(1), 53-58.
446	Sulaiman, S., & Razif, S. M. (2017). Physical activity and body composition among cancer patients at Universiti Kebangsaan Malaysia Medical Center. *Malaysian Journal of Public Health Medicine*. https://www.researchgate.net/publication/320566457
447	Sulaiman, S., Manaf, Z. A., & Shahril, R. (2016). Compliance to Dietary Counselling in Controlling Blood Lipid and its Barriers among Dyslipidemic Individuals. *International Journal on Advanced Science Engineering and Information Technology*, *6*(5), 697-undefined.
448	Sultoni, K., Jajat, & Fitri, M. (2017). Health-Related Fitness Knowledge and Its Relation to College Student Physical Activity. *IOP Conference Series: Materials Science and Engineering*, *180*(1). https://doi.org/10.1088/1757-899X/180/1/012212
449	Sumner, J., Uijtdewilligen, L., Chu, A. H., Ng, S. H., Barreira, T. V., Sloan, R. A., Van Dam, R. M., & Müller-Riemenschneider, F. (2018). Stepping volume and intensity patterns in a multi-ethnic urban Asian population. *BMC Public Health*, *18*(1). https://doi.org/10.1186/s12889-018-5457-y
450	Sun, D. X., Schmidt, G., & Teo-Koh, S. M. (2008). Validation of the RT3 Accelerometer for Measuring Physical Activity of Children in Simulated Free-Living Conditions. *Pediatric Exercise Science, 20*.
451	Suriawati, A. A., Majid, H. A., Al-Sadat, N., Mohamed, M. N. A., & Jalaludin, M. Y. (2016). Vitamin D and calcium intakes, physical activity, and calcaneus BMC among school-going 13-year old Malaysian adolescents. *Nutrients*, *8*(10). https://doi.org/10.3390/nu8100666
452	Suttanon, P., Piriyaprasarth, P., Krootnark, K., & Aranyavalai, T. (2018). Effectiveness of falls prevention intervention programme in community-dwelling older people in Thailand: Randomized controlled trial. *Hong Kong Physiotherapy Journal*, *38*(1), 1–11. https://doi.org/10.1142/S1013702518500014
453	Suwan, K., Hatthachote Phd, P., Panichkul, S., & Phromphetcharat, V. (2012). Comparision of Overweight and Obesity in Medical Cadets Before and After 6 Months Studying at Phramongkutklao College. *Journal of the Medical Association of Thailand*, *95*, 2012. http://jmat.mat.or.th
454	Suwanachaiy, S., Kulaputana, O., & Chaiwanichsiri, D. (2010). Walk performance in Thai men and women: physical activity dependence. *Asian Biomedicine, 4*(1).
455	Suwanpasu, S., Aungsuroch, Y., & Jitapanya, C. (2014). Post-surgical physical activity enhancing program for elderly patients after hip fracture: A randomized controlled trial. *Asian Biomedicine*, *8*(4), 525–532. https://doi.org/10.5372/1905-7415.0804.323
456	Suyoto, P. S. T., Huriyati, E., Susilowati, R., & Julia, M. (2016). Relative validity of administered indonesian version of the short-form international physical activity questionnaire (IPAQ-SF) among obese adolescent girl population. *Pakistan Journal of Nutrition*, *15*(9), 816–820. https://doi.org/10.3923/pjn.2016.816.820
457	Sw, W., Ym, C., & Ts, L. (2011). Correlates of Physical Activity Level among Hemodialysis Patients in Selangor, Malaysia. *Malaysian Journal of Nutrition*, *17*(3), 277–286.
458	Tajik, E., Abdlatiff, L., Adznam, S. N., Awang, H., Yitsiew, C., & Abubakar, A. S. (2017). A study on level of physical activity, depression, anxiety and stress symptoms among adolescents. *Journal of Sports Medicine and Physical Fitness*, *57*(10), 1382–1387. https://doi.org/10.23736/S0022-4707.16.06658-5
459	Tam, C. L., Bonn, G., Yeoh, S. H., & Wong, C. P. (2014). Investigating diet and physical activity in malaysia: Education and family history of diabetes relate to lower levels of physical activity. *Frontiers in Psychology*, *5*(DEC). https://doi.org/10.3389/fpsyg.2014.01328
460	Tan, A. K. G., Dunn, R. A., & Yen, S. T. (2011). Ethnic disparities in metabolic syndrome in Malaysia: An analysis by risk factors. *Metabolic Syndrome and Related Disorders*, *9*(6), 441–451. https://doi.org/10.1089/met.2011.0031
461	Tan, A. K. G., Yen, S. T., Fang, X., & Chiang, F. S. (2019). Body weight and physical activity of adolescents in Malaysia. *International Health*, *11*(2), 150–158. https://doi.org/10.1093/inthealth/ihy072
462	Tan, E., Chung, W., Lew, Y., Chan, M., Wong, T., & Koh, W. (2009). Characteristics, and Disease Control and Complications of Hypertensive Patients in Primary-care—A Community-based Study in Singapore. *Annals Academy of Medicine*, *38*(10), 850–856.
463	Tan, K. L. (2019). Factors influencing physical inactivity among adults in Negeri Sembilan, Peninsular Malaysia. *The Medical Journal of Malaysia*, *74*(5), 389–393.
464	Tan, M. C., Ng, O. C., Wong, T. W., Hejar, A. R., Anthony, J., & Sintonen, H. (2014). The association of cardiovascular disease with impaired health-related quality of life among patients with type 2 diabetes mellitus. *Singapore Medical Journal*, *55*(4), 209–216. https://doi.org/10.11622/smedj.2014054
465	Tan, M. E., Sagayadevan, V., Abdin, E., Picco, L., Vaingankar, J., Chong, S. A., & Subramaniam, M. (2017). Employment status among the Singapore elderly and its correlates. *Psychogeriatrics*, *17*(3), 155–163. https://doi.org/10.1111/psyg.12206
466	Tan, M. M., Ho, W. K., Yoon, S. Y., Mariapun, S., Hasan, S. N., Shin-Chi Lee, D., Hassan, T., Lee, S. Y., Phuah, S. Y., Sivanandan, K., Pei-Sze Ng, P., Rajaram, N., Jaganathan, M., Jamaris, S., Islam, T., Rahmat, K., Fadzli, F., Vijayananthan, A., Rajadurai, P., … Teo, S. H. (2018). A case-control study of breast cancer risk factors in 7,663 women in Malaysia. *PLoS ONE*, *13*(9). https://doi.org/10.1371/journal.pone.0203469
467	Tan, M. Y., & Magarey, J. (2008). Self-care practices of Malaysian adults with diabetes and sub-optimal glycaemic control. *Patient Education and Counseling*, *72*(2), 252–267. https://doi.org/10.1016/j.pec.2008.03.017
468	Tan, M. Y., Magarey, J. M., Chee, S. S., Lee, L. F., & Tan, M. H. (2011). A brief structured education programme enhances self-care practices and improves glycaemic control in Malaysians with poorly controlled diabetes. *Health Education Research*, *26*(5), 896–907. https://doi.org/10.1093/her/cyr047
469	Tan, N. C., Koh, E. Y. L., Goh, C. C., Goh, P. S. C., & Koh, K. H. (2017). A cross-sectional study of gender differences in lifestyle behavior and usage of medications among community-dwelling Asians towards achieving their LDL-Cholesterol treatment goals. *Proceedings of Singapore Healthcare*, *26*(3), 158–165. https://doi.org/10.1177/2010105817694906
470	Tarawan, V. M., Fatimah, S. N., Nurhayati, T., Akbar, M. R., Radhiyanti, P. T., Purba, A., Akbar, I. B., & Goenawan, H. (2018). Association between metabolic syndrome criteria and lifestyle category among university academic staff in West Java, Indonesia. *Pakistan Journal of Nutrition*, *17*(12), 709–714. https://doi.org/10.3923/pjn.2018.709.714
471	Tawe, L., Ng, Z. Y., & Kumar Veerapen, M. (2016). Association of FTO17817449 Variant with Obesity and the Energy Intake and Physical Activity Parameters in Malaysian Adolescents. *Malaysian Journal of Biochemistry and Molecular Biology*. https://www.researchgate.net/publication/306959917
472	Tee, E. S., Nurliyana, A. R., Karim Norimah, A., Jan Mohamed, H. J. B., Tan, S. Y., Appukutty, M., Hopkins, S., Thielecke, F., Ong, M. K., Ning, C., & Mohd Nasir, M. T. (2018). Breakfast consumption among Malaysian primary and secondary school children and relationship with body weight status—Findings from the MyBreakfast Study. *Asia Pacific Journal of Clinical Nutrition*, *27*(2), 421–432. https://doi.org/10.6133/apjcn.062017.12
473	Teh, C. H., Chan, Y. Y., Lim, K. H., Kee, C. C., Lim, K. K., Yeo, P. S., Azahadi, O., Fadhli, Y., Tahir, A., Lee, H. L., & Nazni, W. A. (2015). Association of physical activity with blood pressure and blood glucose among Malaysian adults: A population-based study Chronic Disease epidemiology. *BMC Public Health*, *15*(1). https://doi.org/10.1186/s12889-015-2528-1
474	Teh, C. H., Lim, K. K., Chan, Y. Y., Lim, K. H., Azahadi, O., Hamizatul Akmar, A. H., Ummi Nadiah, Y., Syafinaz, M. S., Kee, C. C., Yeo, P. S., & Fadhli, Y. (2014). The prevalence of physical activity and its associated factors among Malaysian adults: Findings from the National Health and Morbidity Survey 2011. *Public Health*, *128*(5), 416–423. https://doi.org/10.1016/j.puhe.2013.10.008
475	Teh, K. C., Ong, T. H., Teh, D., & Chuan, K. (2004). Physical activity patterns of Singaporeans in 2001. *Singapore Medical Journal, 45*(11).
476	Teo, P. S., Nurul-Fadhilah, A., & Foo, L. H. (2013). Development of a new computer-based physical activity questionnaire to estimate habitual physical activity level in Malaysian adolescents. *Journal of Science and Medicine in Sport*, *16*(4), 327–331. https://doi.org/10.1016/j.jsams.2012.06.012
477	Teo, P. S., Nurul-Fadhilah, A., Aziz, M. E., Hills, A. P., & Foo, L. H. (2014). Lifestyle practices and obesity in Malaysian adolescents. *International Journal of Environmental Research and Public Health*, *11*(6), 5828–5838. https://doi.org/10.3390/ijerph110605828
478	Thanamee, S., Pinyopornpanish, K., Wattanapisit, A., Suerungruang, S., Thaikla, K., Jiraporncharoen, W., & Angkurawaranon, C. (2017). A population-based survey on physical inactivity and leisure time physical activity among adults in Chiang Mai, Thailand, 2014. *Archives of Public Health*, *75*(1). https://doi.org/10.1186/s13690-017-0210-z
479	Thasanasuwan, W., Srichan, W., Kijboonchoo, K., Yamborisut, U., Wimonpeerapattana, W., Rojroongwasinkul, N., Khouw, I. T., & Deurenberg, P. (2016). Low Sleeping Time, High TV Viewing Time, and Physical Inactivity in School Are Risk Factors for Obesity in Pre-Adolescent Thai Children. *Journal of the Medical Association of Thailand*, *99*(3), 314–321. http://www.jmatonline.com
480	Thilarajah, S., Bower, K. J., Pua, Y. H., Tan, D., Williams, G., Larik, A., Bok, C. W., Koh, G., & Clark, R. A. (2020). Modifiable Factors Associated With Poststroke Physical Activity at Discharge From Rehabilitation: Prospective Cohort Study. *Physical Therapy*, *100*(5), 818–828. https://doi.org/10.1093/ptj/pzaa022
481	Thu Hien, V. T., Thi Lam, N., Cong Khan, N., Wakita, A., & Yamamoto, S. (2013). Monosodium glutamate is not associated with overweight in Vietnamese adults. *Public Health Nutrition*, *16*(5), 922–927. https://doi.org/10.1017/S1368980012003552
482	Thuy, A. B., Blizzard, L., Schmidt, M. D., Pham, L. H., Granger, R. H., & Dwyer, T. (2012). Physical activity and its association with cardiovascular risk factors in Vietnam. *Asia-Pacific Journal of Public Health*, *24*(2), 308–317. https://doi.org/10.1177/1010539510379394
483	Ting, J. L. C., Mukherjee, S., & Hwa, M. C. Y. (2015). Physical activity and sedentary behavior patterns of singaporean adolescents. *Journal of Physical Activity and Health*, *12*(9), 1213–1220. https://doi.org/10.1123/jpah.2014-0207
484	Tiong, X. T., Nursara Shahirah, A., Pun, V. C., Wong, K. Y., Fong, A. Y. Y., Sy, R. G., Castillo-Carandang, N. T., Nang, E. E. K., Woodward, M., van Dam, R. M., Tai, E. S., & Venkataraman, K. (2018). The association of the dietary approach to stop hypertension (DASH) diet with blood pressure, glucose and lipid profiles in Malaysian and Philippines populations. *Nutrition, Metabolism and Cardiovascular Diseases*, *28*(8), 856–863. https://doi.org/10.1016/j.numecd.2018.04.014
485	To, Q. G., Gallegos, D., Do, D. V., Tran, H. T. M., To, K. G., Wharton, L., & Trost, S. G. (2018). The level and pattern of physical activity among fifth-grade students in Ho Chi Minh City, Vietnam. *Public Health*, *160*, 18–25. https://doi.org/10.1016/j.puhe.2018.03.021
486	Tok, C. Y., Ahmad, S. R., & Koh, D. S. Q. (2018). Dietary habits and lifestyle practices among university students in universiti Brunei Darussalam. *Malaysian Journal of Medical Sciences*, *25*(3), 56–66. https://doi.org/10.21315/mjms2018.25.3.6
487	Tran, D. V., Lee, A. H., Au, T. B., Nguyen, C. T., & Hoang, D. V. (2013). Reliability and validity of the International Physical Activity Questionnaire-Short Form for older adults in Vietnam. *Health Promotion Journal of Australia*, *24*(2), 126–131. https://doi.org/10.1071/HE13012
488	Tran, N. T. T., Blizzard, C. L., Luong, K. N., Truong, N. L. V., Tran, B. Q., Veloudi, P., Otahal, P., Nelson, M., Magnussen, C., Gall, S., Bui, T. V., Srikanth, V., Au, T. B., Ha, S. T., Phung, H. N., Tran, M. H., Callisaya, M., & Sharman, J. (2018). Misclassification of blood pressure of Vietnamese adults when only a single measurement is used. *Journal of the American Society of Hypertension*, *12*(9), 671–680. https://doi.org/10.1016/j.jash.2018.06.015
489	Tran, V. D., Lee, A. H., Jancey, J., James, A. P., Howat, P., & Mai, L. T. P. (2017). Physical activity and nutrition behaviour outcomes of a cluster-randomized controlled trial for adults with metabolic syndrome in Vietnam. *Trials*, *18*(1). https://doi.org/10.1186/s13063-016-1771-9
490	Trang, N. H. H. D., Hong, T. K., & Dibley, M. J. (2012). Active commuting to school among adolescents in Ho Chi Minh City, Vietnam: Change and predictors in a longitudinal study, 2004 to 2009. *American Journal of Preventive Medicine*, *42*(2), 120–128. https://doi.org/10.1016/j.amepre.2011.10.006
491	Trang, N. H. H. D., Hong, T. K., & Dibley, M. J. (2012). Cohort profile: Ho Chi Minh City Youth cohort-changes in diet, physical activity, sedentary behaviour and relationship with overweight/obesity in adolescents. *BMJ Open*, *2*(1). https://doi.org/10.1136/bmjopen-2011-000362
492	Trang, N. H. H. D., Hong, T. K., Dibley, M. J., & Sibbritt, D. W. (2009). Factors associated with physical inactivity in adolescents in ho chi minh city, vietnam. *Medicine and Science in Sports and Exercise*, *41*(7), 1374–1383. https://doi.org/10.1249/MSS.0b013e31819c0dd3
493	Trang, N. H. H. D., Hong, T. K., Van Der Ploeg, H. P., Hardy, L. L., Kelly, P. J., & Dibley, M. J. (2012). Longitudinal physical activity changes in adolescents: Ho Chi Minh City Youth Cohort. *Medicine and Science in Sports and Exercise*, *44*(8), 1481–1489. https://doi.org/10.1249/MSS.0b013e31824e50dc
494	Tridiantari, D. K., Saraswati, L. D., & Udiyono, A. (2020). Epidemiology of erectile dysfunction in men with diabetes mellitus: A study in a primary health care center in Indonesia. *Medical Journal of Indonesia*, *29*(1), 82–87. https://doi.org/10.13181/mji.oa.192070
495	Trihandini, I. (2015). The Relationship between Smoking as a Modifiable Risk Factor and Chronic Complications on Elderly with Type 2 Diabetes Mellitus. *Makara Journal of Health Research*, *19*(1). https://doi.org/10.7454/msk.v19i1.4598
496	Trinh, O. T. H., Nguyen, N. D., Dibley, M. J., Phongsavan, P., & Bauman, A. E. (2008). The prevalence and correlates of physical inactivity among adults in Ho Chi Minh City. *BMC Public Health*, *8*. https://doi.org/10.1186/1471-2458-8-204
497	Trinh, O. T. H., Nguyen, N. D., Phongsavon, P., Dibley, M. J., & Bauman, A. E. (2010). Metabolic risk profiles and associated risk factors among vietnamese adults in Ho Chi Minh City. *Metabolic Syndrome and Related Disorders*, *8*(1), 69–78. https://doi.org/10.1089/met.2009.0018
498	Trinh, O. T. H., Nguyen, N. Do, Van Der Ploeg, H. P., Dibley, M. J., & Bauman, A. (2009). Test-retest repeatability and relative validity of the global physical activity questionnaire in a developing country context. *Journal of Physical Activity and Health*, *6*(SUPPL. 1). https://doi.org/10.1123/jpah.6.s1.s46
499	Tuazon, M. A. G. (1987). *Maternal energy requirements during pregnancy of rural Philippine women*. https://research.wur.nl/en/publications/maternal-energy-requirements-during-pregnancy-of-rural-philippine-2
500	Tudor-Locke, C., Ainsworth, B. E., Adair, L. S., & Popkin, B. M. (2003). Physical activity in Filipino youth: The Cebu Longitudinal Health and Nutrition Survey. *International Journal of Obesity*, *27*(2), 181–190. https://doi.org/10.1038/sj.ijo.802207
501	Tudor-Locke, C., Ainsworth, B. E., Adair, L. S., & Popkin, B. M. (2003). Objective physical activity of Filipino youth stratified for commuting mode to school. *Medicine and Science in Sports and Exercise*, *35*(3), 465–471. https://doi.org/10.1249/01.MSS.0000053701.30307.A6
502	Tudor-Locke, C., Ainsworth, B. E., Adair, L. S., Du, S., Lee, N., & Popkin, B. M. (2007). Cross-sectional comparison of physical activity and inactivity patterns in Chinese and Filipino youth. *Child: Care, Health and Development*, *33*(1), 59–66. https://doi.org/10.1111/j.1365-2214.2006.00612.x
503	Tung, S. E. H., Ng, X. H., Chin, Y. S., & Mohd Taib, M. N. (2016). Associations between parents’ perception of neighbourhood environments and safety with physical activity of primary school children in Klang, Selangor, Malaysia. *Child: Care, Health and Development*, *42*(4), 478–485. https://doi.org/10.1111/cch.12355
504	Uijtdewilligen, L., Waters, C. N. H., Aw, S., Wong, M. L., Sia, A., Ramiah, A., Wong, M., & Müller-Riemenschneider, F. (2019). The Park prescription study: Development of a community-based physical activity intervention for a multi-ethnic Asian population. *PLoS ONE*, *14*(6). https://doi.org/10.1371/journal.pone.0218247
505	Uijtdewilligen, L., Yin, J. D. C., van der Ploeg, H. P., & Müller-Riemenschneider, F. (2017). Correlates of occupational, leisure and total sitting time in working adults: Results from the Singapore multi-ethnic cohort. *International Journal of Behavioral Nutrition and Physical Activity*, *14*(1). https://doi.org/10.1186/s12966-017-0626-4
506	Utami, G. T., Woferst, R., & Lubis, S. L. (2019). An overview of physical activities among family members with risk of type 2 diabetes mellitus in Pekanbaru. *Enfermeria Clinica*, *29*, 26–29. https://doi.org/10.1016/j.enfcli.2018.11.012
507	Uy, Y., Jf, Y., & Wy, C. (2016). Prevalence of falls among community-dwelling elderly and its associated factors: A cross-sectional study in Perak, Malaysia. *Malaysian Family Physician, 11*(1).
508	Vaingankar, J. A., Chong, S. A., Abdin, E., Picco, L., Chua, B. Y., Shafie, S., Ong, H. L., Chang, S., Seow, E., Heng, D., Chiam, P. C., & Subramaniam, M. (2017). Prevalence of frailty and its association with sociodemographic and clinical characteristics, and resource utilization in a population of Singaporean older adults. *Geriatrics and Gerontology International*, *17*(10), 1444–1454. https://doi.org/10.1111/ggi.12891
509	Van Bui, T., Blizzard, C. L., Luong, K. N., Van Truong, N. Le, Tran, B. Q., Otahal, P., Srikanth, V., Nelson, M. R., Au, T. B., Ha, S. T., Phung, H. N., Tran, M. H., Callisaya, M., & Gall, S. (2015). Physical activity in Vietnam: Estimates and measurement issues. *PLoS ONE*, *10*(10). https://doi.org/10.1371/journal.pone.0140941
510	Van Do, V., Jancey, J., Pham, N. M., Nguyen, C. T., Van Hoang, M., & Lee, A. H. (2019). Objectively measured physical activity of Vietnamese adults with type 2 diabetes: Opportunities to intervene. *Journal of Preventive Medicine and Public Health*, *52*(2), 101–108. https://doi.org/10.3961/jpmph.18.213
511	Vasquez, K., Malhotra, R., Østbye, T., Chan, M. F., Amin, H., Khoo, G., Choo, L., Chew, L., & Thilagaratnam, S. (2015). Extent and correlates of change in anthropometric and fitness outcomes among participants in a corporate team-based weight loss challenge in Singapore: Lose to win 2009. *Asia-Pacific Journal of Public Health*, *27*(2), NP425–NP436. https://doi.org/10.1177/1010539512455044
512	Vathesatogkit, P., Sritara, P., Kimman, M., Hengprasith, B., E-Shyong, T., Wee, H. L., & Woodward, M. (2012). Associations of Lifestyle Factors, Disease History and Awareness with Health-Related Quality of Life in a Thai Population. *PLoS ONE*, *7*(11). https://doi.org/10.1371/journal.pone.0049921
513	Venkataraman, K., Wee, H. L., & Ng, S. (2016). Determinants of individuals’ participation in integrated chronic disease screening in Singapore. *Journal of Epidemiology and Community Health*, *70*, 1242–1250. https://doi.org/10.1136/jech
514	Vo Van Ha, A., Zhao, Y., Binns, C. W., Pham, N. M., Nguyen, P. T. H., Nguyen, C. L., Chu, T. K., & Lee, A. H. (2020). Postpartum physical activity and weight retention within one year: A prospective cohort study in Vietnam. *International Journal of Environmental Research and Public Health*, *17*(3). https://doi.org/10.3390/ijerph17031105
515	Vonglokham, M., Kounnavong, S., Sychareun, V., Pengpid, S., & Peltzer, K. (2019). Prevalence and social and health determinants of pre-diabetes and diabetes among adults in Laos: a cross-sectional national population-based survey, 2013. *Tropical Medicine and International Health*, *24*(1), 65–72. https://doi.org/10.1111/tmi.13164
516	Vu, T. H. L., Bui, T. T. Q., Nguyen, T. K. N., & Hoang, V. M. (2020). Adverse influence of multilevel socioeconomic status on physical activity: Results from a national survey in Vietnam. *BMC Public Health*, *20*(1). https://doi.org/10.1186/s12889-020-08695-5
517	Wafa, S. W. W. bte S. S. T., Shahril, M. R. bin, Ahmad, A. bte, Zainuddin, L. R. bte, Ismail, K. F. bte, Aung, M. M. T., & Mohd Yusoff, N. A. bte. (2016). Association between physical activity and health-related quality of life in children: A cross-sectional study. *Health and Quality of Life Outcomes*, *14*(1). https://doi.org/10.1186/s12955-016-0474-y
518	Wafa, S. W., Hamzaid, H., Talib, R. A., & Reilly, J. J. (2014). Objectively measured habitual physical activity and sedentary behaviour in obese and non-obese Malaysian children. *Journal of Tropical Pediatrics*, *60*(2), 161–163. https://doi.org/10.1093/tropej/fmt093
519	Wafa, S. W., Talib, R. A., Hamzaid, N. H., McColl, J. H., Rajikan, R., Ng, L. O., Ramli, A. H., & Reilly, J. J. (2011). Randomized controlled trial of a good practice approach to treatment of childhood obesity in Malaysia: Malaysian Childhood Obesity Treatment Trial (MASCOT). *International Journal of Pediatric Obesity*, *6*(2–2). https://doi.org/10.3109/17477166.2011.566340
520	Wahida, F., Nasir, M., & As, H. (2011). Eating Behaviour and Body Image Perception among Young Adolescents 325. *Physical Activity, 17*(3).
521	Wanaratna, K., Muangpaisan, W., Kuptniratsaikul, V., Chalermsri, C., & Nuttamonwarakul, A. (2019). Prevalence and Factors Associated with Frailty and Cognitive Frailty Among Community-Dwelling Elderly with Knee Osteoarthritis. *Journal of Community Health*, *44*(3), 587–595. https://doi.org/10.1007/s10900-018-00614-5
522	Wang, C. K. J., Biddle, S. J. H., Liu, W. C., & Lim, B. S. C. (2012). A latent profile analysis of sedentary and physical activity patterns. *Journal of Public Health (Germany)*, *20*(4), 367–373. https://doi.org/10.1007/s10389-011-0464-9
523	Wang, Y., & Chia, M. (2010). Validity and reliability of OMRON HJ-005 pedometer in quantifying field-based physical activity among Singaporean children. *Sport Science and Studies in Asia*—*Issues, Reflections and Emergent Solutions*. www.worldscientific.com
524	Wattanapisit, A., Fungthongcharoen, K., Saengow, U., & Vijitpongjinda, S. (2016). Physical activity among medical students in Southern Thailand: A mixed methods study. *BMJ Open*, *6*(9). https://doi.org/10.1136/bmjopen-2016-013479
525	Wattanapisit, A., Saengow, U., Ng, C. J., Thanamee, S., & Kaewruang, N. (2018). Gaming behaviour with pokémon GO and physical activity: A preliminary study with medical students in Thailand. *PLoS ONE*, *13*(6). https://doi.org/10.1371/journal.pone.0199813
526	Wattanapisit, A., Vijitpongjinda, S., Saengow, U., Amaek, W., Thanamee, S., & Petchuay, P. (2018). Results from the Medical School Physical Activity Report Card (MSPARC) for a Thai Medical School: A mixed methods study 11 Medical and Health Sciences 1117 Public Health and Health Services 13 Education 1303 Specialist Studies in Education. *BMC Medical Education*, *18*(1). https://doi.org/10.1186/s12909-018-1408-7
527	Wee, J., Sng, B. Y. J., Shen, L., Lim, C. T., Singh, G., & Das De, S. (2013). The relationship between body mass index and physical activity levels in relation to bone mineral density in premenopausal and postmenopausal women. *Archives of Osteoporosis*, *8*(1–2). https://doi.org/10.1007/s11657-013-0162-z
528	Weng Fong, C., & Bhalla, V. (2007). Educational inequalities associated with health-related behaviours in the adult population of Singapore. *Singapore Medical Journal, 48*(12). https://www.researchgate.net/publication/5803715
529	Whye Sook, L., Izzah Aini Sablihan, N., Ismail, S., Kumar Devaraj, N., & Siew Mooi, C. (2019). Factors Associated With the Level of Physical Activities Among Non-Academic Staffs in the. *Malaysian Journal of Medicine and Health Sciences, 15*(2).
530	Widajanti, N., Ichwani, J., Dharmanta, R. S., Firdausi, H., Haryono, Y., Yulianti, E., Kandinata, S. G., Wulandari, M., Widyasari, R., Adyanita, V. A., Hapsanti, N. I., Wardana, D. M., Kristiana, T., Wahyuda, H., Yunara, S., & Husna, K. (2020). Sarcopenia and Frailty Profile in the Elderly Community of Surabaya: A Descriptive Study. *Acta Medica Indonesiana, 52*.
531	Widjaja, F. F., Santoso, L. A., Barus, N. R. V., Pradana, G. A., & Estetika, C. (2013). Prehypertension and hypertension among young Indonesian adults at a primary health care in a rural area. *Medical Journal of Indonesia*, *22*(1), 39–45. https://doi.org/10.13181/mji.v22i1.519
532	Widyastuti, K., Makhabah, D. N., Setijadi, A. R., Sutanto, Y. S., Suradi, & Ambrosino, N. (2018). Benefits and costs of home pedometer assisted physical activity in patients with COPD. A preliminary randomized controlled trial. *Pulmonology*, *24*(4), 211–218. https://doi.org/10.1016/j.pulmoe.2018.01.006
533	Win, A. M., Yen, L. W., Tan, K. H., Lim, R. B. T., Chia, K. S., & Mueller-Riemenschneider, F. (2015). Patterns of physical activity and sedentary behavior in a representative sample of a multi-ethnic South-East Asian population: A cross-sectional study. *BMC Public Health*, *15*(1). https://doi.org/10.1186/s12889-015-1668-7
534	Winkvist, A., Persson, V., & Hartini, T. N. S. (2002). Underreporting of energy intake is less common among pregnant women in Indonesia. *Public Health Nutrition*, *5*(4), 523–529. https://doi.org/10.1079/phn2001317
535	Wong, C. H., Wong, S. F., Pang, W. S., Azizah, M. Y., & Dass, M. J. (2003). Habitual walking and its correlation to better physical function: Implications for prevention of physical disability in older persons. *Journals of Gerontology Series A Biological Sciences and Medical Sciences*, *58*(6), 555–560. https://doi.org/10.1093/gerona/58.6.m555
536	Wong, C. H., Wong, S., & Shen, L. (2003). Correlates of habitual walking and sports/leisure-time physical activity in older persons in Singapore: interaction effects between educational attainment and gender. *Annals of the Academy of Medicine, Singapore*, *32*(6), 801–806. https://www.researchgate.net/publication/8924059
537	Wong, J. E., Parikh, P., Poh, B. K., & Deurenberg, P. (2016). Physical Activity of Malaysian Primary School Children: Comparison by Sociodemographic Variables and Activity Domains. *Asia-Pacific Journal of Public Health*, *28*, 35S-46S. https://doi.org/10.1177/1010539516650726
538	Wong, M. L., Koh, D., & Lee, M. H. (1998). Assess workers’ needs and preferences first before planning a physical fitness programme: findings from a polytechnic institute in Singapore. *Occupational Medicine, 48*(1). http://occmed.oxfordjournals.org/
539	Wong, W. P., Zhao, Y., & Koh, W.-P. (2012). Gene Polymorphism in Angiotensin-I-Converting Enzyme and Physical Activity Among Normotensive Chinese. *International Journal of Sport Nutrition and Exercise Metabolism, 22*.
540	Woon, F. C., Chin, Y. S., & Mohd Nasir, M. T. (2015). Association between behavioural factors and BMI-for-age among early adolescents in Hulu Langat district, Selangor, Malaysia. *Obesity Research and Clinical Practice*, *9*(4), 346–356. https://doi.org/10.1016/j.orcp.2014.10.218
541	Woratanarat, P., Wajanavisit, W., Woratanarat, T., & Suriyawongpaisal, P. (2009). A comparative study of risk factors of femoral neck and intertrochanteric fracture in Thai men. *Journal of the Medical Association of Thailand = Chotmaihet thangphaet*. https://www.researchgate.net/publication/41396125
542	Woratanarat, P., Wajanavisit, W., Woratanarat, T., & Suriyawongpaisal, P. (2009). Different risk magnitudes of femoral neck and intertrochanteric fractures in Thai women. *Journal of the Medical Association of Thailand = Chotmaihet thangphaet*. https://www.researchgate.net/publication/41396126
543	Wss, C. (2015). Studying the Family Diet: An Investigation into Association Between Diet, Lifestyle and Weight Status in Malaysian Families. *Malaysian Journal of Nutrition, 21*(2).
544	Yamborisut, U., Kosulwat Phd, V., Chittchang Dsc, U., Wimonpeerapattana Msc, W., & Suthutvoravut, U. (2006). Factors Associated with Dual Form of Malnutrition in School Children in Nakhon Pathom and Bangkok. *Journal of the Medical Association of Thailand, 89*(7).
545	Yatim, H. M., Wong, Y. Y., Lim, S. H., Hassali, M. A., Hong, Y. H., Dali, A. F., & Neoh, C. F. (2018). Evaluation of a group-based hypertension self-management education programme among hypertensive community dwellers. *European Journal of Integrative Medicine*, *24*, 79–84. https://doi.org/10.1016/j.eujim.2018.10.016
546	Yaw, Y. H., Shariff, Z. M., Kandiah, M., Mun, C. Y., Yusof, R. M., Othman, Z., Saibul, N., Weay, Y. H., & Hashim, Z. (2011). Weight changes and lifestyle behaviors in women after breast cancer diagnosis: A cross-sectional study. *BMC Public Health*, *11*. https://doi.org/10.1186/1471-2458-11-309
547	Yaw, Y. H., Shariff, Z. M., Kandiah, M., Weay, Y. H., Saibul, N., Sariman, S., & Hashim, Z. (2014). Diet and physical activity in relation to weight change among breast cancer patients. *Asian Pacific Journal of Cancer Prevention*, *15*(1), 39–44. https://doi.org/10.7314/APJCP.2014.15.1.39
548	Yee, Y., Zaitun, Y., Chan, Y., & Norhaizan, M. (2013). Association between anthropometic status, dietary intake and physical activity with health bone status among premenopausal chinese women in Klang Valley, Malaysia. *Malaysian Journal of Nutrition*, *19*(3), 292–302.
549	Yen, S., Knight, A., Krishna, M., Muda, W., & Rufai, A. (2016). Lifetime Physical Activity and Breast Cancer: a Case-Control Study in Kelantan, Malaysia. *Asian Pacific Journal of Cancer Prevention*, *17*(8), 4083–4088.
550	Yen, Y., Shi, Y., Soeung, B., Seng, R., Dy, C., Suy, R., & Ngin, K. (2018). The associated risk factors for underweight and overweight high school students in Cambodia. *Diabetes and Metabolic Syndrome: Clinical Research and Reviews*, *12*(5), 737–742. https://doi.org/10.1016/j.dsx.2018.04.016
551	Yi, S., Ngin, C., Peltzer, K., & Pengpid, S. (2017). Health and behavioral factors associated with binge drinking among university students in nine ASEAN countries. *Substance Abuse: Treatment, Prevention, and Policy*, *12*(1). https://doi.org/10.1186/s13011-017-0117-2
552	Yim Loh, S. (2011). 1483 Barriers to Exercise: Perspectives from Multiethnic Cancer Survivors Asian Pacific. *Asian Pacific Journal of Cancer Prevention, 12*.
553	Ying, C. Y., Lim, K. K., Teh, C., Lim, K., Abd Hamid, H., Omar, M., Ahmad, N., & Kee, C. (2014). Prevalence and factors associated with physical inactivity among Malaysian adults. *The Southeast Asian Journal of Tropical Medicine and Public Health*, *45*(2), 467–480. https://www.researchgate.net/publication/331918744
554	Yk, A., Mirnalini K, & Zalilah. (2013). Email-linked Website Intervention for Modifying Cancer-related Dietary and Lifestyle Risk Factors A Workplace Email-linked Website Intervention for Modifying Cancer-related Dietary and Lifestyle Risk Factors: Rationale, Design and Baseline Findings. *Malaysian Journal of Nutrition, 19*(1).
555	Yong, H. Y., Mohd Shariff, Z., Koo, S. J., & Binti Sa’ari, N. S. (2016). Pre-pregnancy body mass index, height and physical activity are associated with rate of gestational weight gain among Malaysian mothers. *Journal of Obstetrics and Gynaecology Research*, *42*(9), 1094–1101. https://doi.org/10.1111/jog.13039
556	Yong, H. Y., Zalilah, M. S., Tan, C. W., & Koo, S. J. (2017). Pre-pregnancy BMI and intake of energy and calcium are associated with the vitamin D intake of pregnant Malaysian women. *Family Medicine and Primary Care Review*, *19*(4), 417–423. https://doi.org/10.5114/fmpcr.2017.70819
557	Yuenyongchaiwat, K., Pongpanit, K., & Hanmanop, S. (2018). Atividade física e depressão em adultos idosos com e sem comprometimento cognitive. *Dementia e Neuropsychologia*, *12*(1), 12–18. https://doi.org/10.1590/1980-57642018dn12-010002
558	Yusainy, C., Chan, D. K. C., Hikmiah, Z., & Anggono, C. O. (2019). Physical activity in Indonesian University students: the contradictory roles of dispositional mindfulness and self-control. *Psychology, Health and Medicine*, *24*(4), 446–455. https://doi.org/10.1080/13548506.2018.1546015
559	Zaharan, N. L., Muhamad, N. H., Jalaludin, M. Y., Su, T. T., Mohamed, Z., Mohamed, M. N. A., & Majid, H. A. (2018). Non-synonymous single-nucleotide polymorphisms and physical activity interactions on adiposity parameters in Malaysian adolescents. *Frontiers in Endocrinology*, *9*(APR). https://doi.org/10.3389/fendo.2018.00209
560	Zahtamal, Rochmah, W., Prabandari, Y. S., & Setyawati, L. K. (2017). Effects of multilevel intervention in workplace health promotion on workers’ metabolic syndrome components. *Kesmas*, *11*(4), 198–204. https://doi.org/10.21109/kesmas.v11i4.1279
561	Zaid, Z. A., & Kandiah, M. (2012). Relationship between nutritional status, physical activity and quality of life among gastrointestinal cancer survivors. *Malaysian Journal of Nutrition*. https://www.researchgate.net/publication/260428531
562	Zalbahar, N., Jan Mohamed, H. J. B., Loy, S. L., Najman, J., McIntyre, H. D., & Mamun, A. (2016). Association of parental body mass index before pregnancy on infant growth and body composition: Evidence from a pregnancy cohort study in Malaysia. *Obesity Research and Clinical Practice*, *10*, S35–S47. https://doi.org/10.1016/j.orcp.2015.08.002
563	Zulfarina, M. S., Sharif, R., Syarifah-Noratiqah, S. B., Sharkawi, A. M., Aqilah, Z. S., Mokhtar, S. A., Nazrun, S. A., & Naina-Mohamed, I. (2018). Modifiable factors associated with bone health in Malaysian adolescents utilising calcaneus quantitative ultrasound. *PLoS ONE*, *13*(8). https://doi.org/10.1371/journal.pone.0202321
564	Zulkifli, A. F. (2019). Student-centered approach and alternative assessments to improve students’ learning domains during health education sessions. *Biomedical Human Kinetics*, *11*(1), 80–86. https://doi.org/10.2478/bhk-2019-0010
